# A Multi-Strategy Secretary Bird Optimization Algorithm for Aesthetic Color and Layout Optimization in Visual Art Design

**DOI:** 10.3390/biomimetics11080533

**Published:** 2026-08-01

**Authors:** Lin Zhou, Xinyu Cai

**Affiliations:** 1 College of Design, Jiaxing University, Jiaxing 314001, China; zhoulin@zjxu.edu.cn; 2 College of Business, Jiaxing University, Jiaxing 314001, China

**Keywords:** secretary bird optimization algorithm, metaheuristic algorithm, color harmony optimization, aesthetic layout design, lens opposition-based learning, computational aesthetics

## Abstract

Visual art and graphic design tasks, such as generating a harmonious color palette or arranging the elements of a page, can be naturally formulated as mathematical optimization problems whose objective functions are non-convex, multimodal, and non-differentiable. Metaheuristic algorithms are well-suited to such problems. The secretary bird optimization algorithm (SBOA) is a recently proposed bio-inspired metaheuristic that mimics the hunting and predator-escaping behaviors of secretary birds, and it has shown competitive performance. However, SBOA still suffers from insufficient population diversity, premature convergence, and an unbalanced transition between exploration and exploitation, which limits its accuracy on complex design problems. To overcome these limitations, this paper proposes a multi-strategy secretary bird optimization algorithm (MSSBOA) that integrates three improvement strategies. First, a good point set initialization is employed to generate a low-discrepancy initial population that covers the search space more uniformly and enriches population diversity. Second, a lens opposition-based learning strategy is applied to the inferior individuals to help the population escape local optima while preserving the elite. Third, an adaptive Cauchy–Gaussian mutation is imposed on the best individual to balance global exploration and local exploitation throughout the search. The performance of MSSBOA is comprehensively examined on the CEC2017 benchmark suite in 10, 30, 50, and 100 dimensions and compared with the basic SBOA and nine state-of-the-art algorithms; the results are analyzed by the Friedman test, the Nemenyi post hoc test and the Wilcoxon rank-sum test. MSSBOA is then applied to two representative visual-design optimization problems: aesthetic color-harmony palette generation and graphic-layout aesthetics optimization. In all cases, MSSBOA outperforms the basic SBOA and the competing algorithms in terms of convergence speed, stability, and solution quality, confirming its effectiveness for computational-aesthetics applications in art design.

## 1. Introduction

With the rapid development of the digital creative industry, art and design activities increasingly rely on computational tools to improve both efficiency and quality [1,2]. Many tasks in visual art and graphic design—such as selecting a harmonious color palette, arranging the elements of a poster, or tuning the form parameters of a product—can be abstracted as the search for an optimal solution within a large feasible space [3]. Such design optimization problems aim to maximize or minimize one or more aesthetic objective functions while satisfying perceptual and functional constraints, and they are widespread in fields ranging from visual communication and packaging to interface and environmental design [4]. Unfortunately, these objectives are usually nonlinear, multimodal, non-differentiable, and strongly coupled with human perception, which makes them extremely difficult for classical optimization techniques [5]. Mathematically, many of them belong to the class of NP-hard problems, for which an exact optimum cannot be obtained in polynomial time [6,7]. Optimization methods are generally divided into deterministic and stochastic categories. Deterministic algorithms, such as linear, branch-and-bound, and dynamic programming, rely on rigorous mathematical properties, such as continuity, convexity, and differentiability [8]. When confronted with non-differentiable objectives or numerous local optima, they easily fall into local optimality and exhibit limited adaptability [9]. Consequently, there is an urgent need for more efficient, stable, and broadly applicable algorithms to cope with the increasingly complex design solution space.

**Figure 1:**
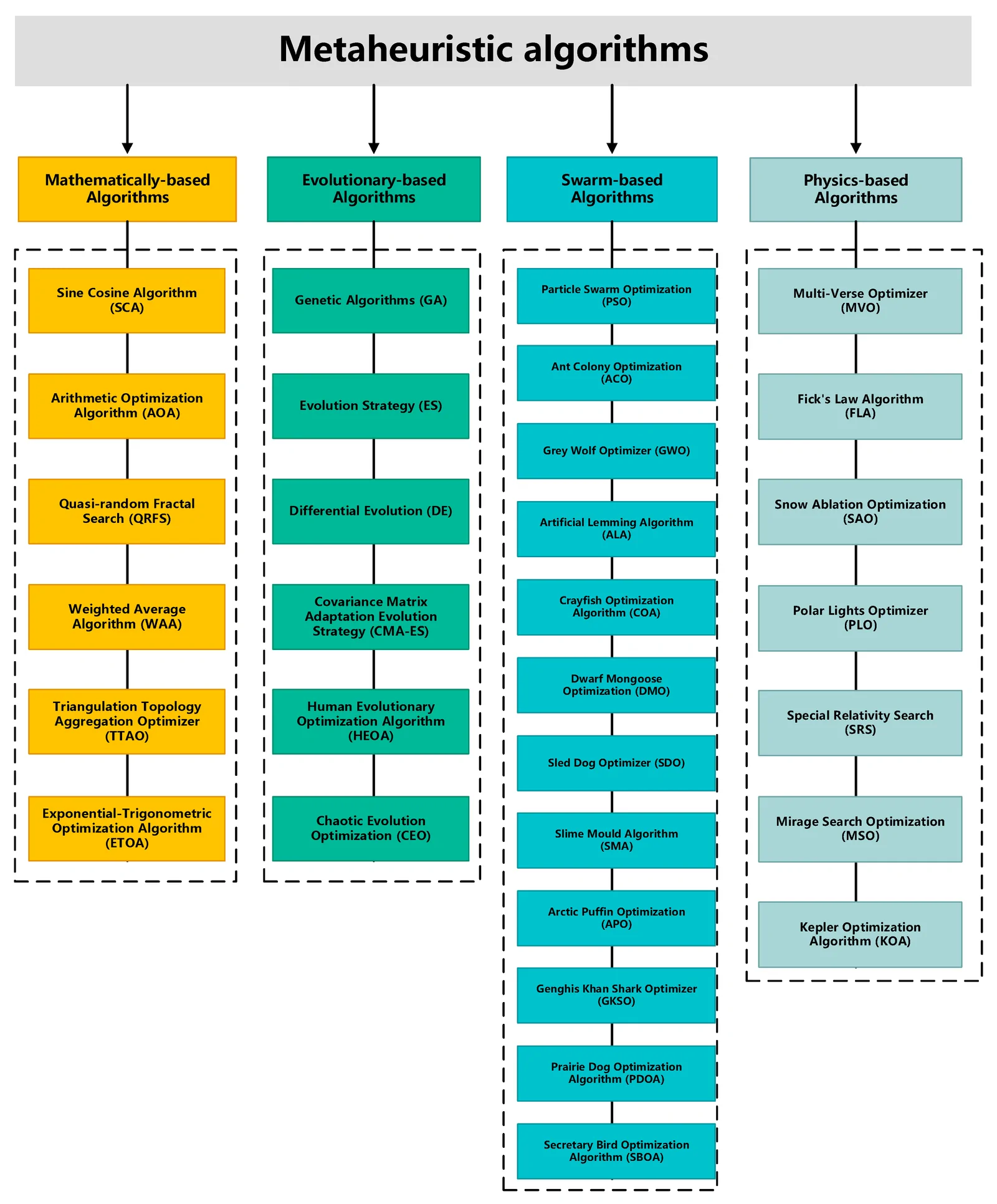
Classification of metaheuristic algorithms.

Metaheuristic algorithms are stochastic optimization techniques that require no gradient information and instead use probabilistic strategies to iteratively explore and exploit the problem space [10]. By balancing global exploration with local exploitation, they effectively mitigate premature convergence and can obtain near-optimal solutions at an acceptable computational cost [11,12]. Because they are flexible, metaheuristics have been successfully applied to path planning [13,14], parameter extraction [15], wireless sensor network coverage [16], image segmentation [17,18], and feature selection [19,20]. Drawing inspiration from nature and social systems, metaheuristic algorithms can be grouped into four primary categories according to their source of inspiration, as shown in Figure 1. Evolution-based algorithms imitate natural selection, mutation, and crossover, including the genetic algorithm (GA) [21], evolution strategy (ES) [22], differential evolution (DE) [23], the covariance matrix adaptation evolution strategy (CMA-ES) [24], the human evolutionary optimization algorithm (HEOA) [25], and chaotic evolution optimization (CEO) [26]. Physics-based algorithms draw on physical laws, such as the multi-verse optimizer (MVO) [27], Fick’s law algorithm (FLA) [28], snow ablation optimization (SAO) [29], the polar lights optimizer (PLO) [30], special relativity search (SRS) [31], mirage search optimization (MSO) [32], and the Kepler optimization algorithm (KOA) [33]. Mathematics-based algorithms are inspired by mathematical functions and theories, such as the sine cosine algorithm (SCA) [34], the arithmetic optimization algorithm (AOA) [35], quasi-random fractal search (QRFS) [36], the weighted average algorithm (WAA) [37], the triangulation topology aggregation optimizer (TTAO) [38], and the exponential-trigonometric optimization algorithm (ETOA) [39]. Swarm-based algorithms simulate the collective behavior of groups of organisms, including particle swarm optimization (PSO) [40], ant colony optimization (ACO) [41], the grey wolf optimizer (GWO) [42], the artificial lemming algorithm (ALA) [43], the crayfish optimization algorithm (COA) [44], dwarf mongoose optimization (DMO) [45], the sled dog optimizer (SDO) [46], the slime mold algorithm (SMA) [47], Arctic puffin optimization (APO) [48], the Genghis Khan shark optimizer (GKSO) [49], the prairie dog optimization algorithm (PDOA) [50], and the secretary bird optimization algorithm (SBOA) [51].

Despite their success, metaheuristic algorithms still face challenges. They can readily obtain good solutions but rarely guarantee the global optimum, and they often struggle to balance exploration and exploitation, so that the population may homogenize and become trapped in local optima. More importantly, the no free lunch theorem proves that no single algorithm can outperform all others on every possible problem [52]. This motivates researchers to design or improve algorithms tailored to specific problem classes—here, the aesthetic optimization problems that arise in visual art and graphic design.

The secretary bird optimization algorithm (SBOA) is a bio-inspired metaheuristic proposed by Fu et al. in 2024 [51]. It models the survival behavior of secretary birds, using a three-stage strategy for hunting snakes during the exploration phase and two strategies for escaping predators during the exploitation phase. Owing to its simple structure and strong search ability, SBOA has attracted considerable attention, and several improved variants have been developed. Qin et al. proposed a multi-strategy improvement SBOA that incorporates incremental-PID feedback regulation and cooperative camouflage to solve engineering problems [53]. Wang et al. enhanced SBOA with multi-strategy fusion and a Cauchy–Gaussian crossover to improve convergence accuracy [54]. Mai et al. integrated logistic-tent chaotic initialization and a crossover strategy into SBOA for engineering design [55], and Seyyedabbasi developed a multi-strategy variable SBOA for global optimization and UAV path planning [56]. These studies enhance SBOA from different angles, yet the inability of the basic SBOA to maintain population diversity and to balance exploration and exploitation in complex, perception-driven design problems remains an objective limitation. Guided again by the no free lunch theorem, this paper proposes a new SBOA variant, the multi-strategy secretary bird optimization algorithm (MSSBOA), and applies it to aesthetic optimization in visual art design.

The choice of SBOA as the basis of this work deserves explicit justification, since many metaheuristic algorithms could, in principle, have been improved instead. Three reasons motivated this choice. First, and most importantly, the structure of SBOA matches the structure of the design problems studied here. Aesthetic objectives, such as Equations (19) and (22), are sums of competing terms whose trade-off surface has many broad, shallow optima rather than one sharp optimum, so an algorithm must survey widely before committing. SBOA is one of the few recent metaheuristics that splits its exploration phase into three explicitly time-gated stages with qualitatively different step distributions—a differential perturbation, a Brownian move towards the elite, and a Levy flight—which gives a scheduled and inspectable progression from survey to commitment, and which provides well-defined points at which further operators can be inserted. Second, SBOA is economical: it has a single control parameter beyond the population size, so a study of added operators is not confounded by the tuning of the base algorithm. Third, SBOA is recent enough that its weaknesses are documented but not yet addressed for this problem class, and it has been shown competitive on standard suites [51], which makes it a meaningful baseline rather than a strawman.

MSSBOA combines three improvement techniques. First, a good point set initialization replaces the random initialization of SBOA, generating a low-discrepancy population that uniformly covers the search domain and enhances population diversity from the outset. Second, a lens opposition-based learning strategy is applied to the inferior individuals to generate refracted candidate solutions, which strengthens the ability of the algorithm to escape local optima while protecting the elite individuals. Third, an adaptive Cauchy–Gaussian mutation is imposed on the best individual, where a Cauchy-dominated perturbation promotes global exploration in the early stage and a Gaussian-dominated perturbation refines the solution in the later stage, thereby balancing exploration and exploitation. MSSBOA was comprehensively examined on the CEC2017 benchmark functions and compared with the basic SBOA and several advanced algorithms, with the differences analyzed by the Friedman test, the Nemenyi post hoc test and the Wilcoxon rank-sum test. Finally, MSSBOA was applied to color-harmony palette optimization and graphic-layout aesthetics optimization to confirm its ability to solve realistic art-design problems. The main contributions of this paper are as follows:

(1) An improved SBOA variant, namely MSSBOA, is proposed by integrating good point set initialization, lens opposition-based learning, and adaptive Cauchy–Gaussian mutation.
(2) The performance of MSSBOA was comprehensively examined on the CEC2017 test suite in four dimensions and confirmed by the Friedman test, the Nemenyi test and the Wilcoxon rank-sum test.
(3) MSSBOA was successfully applied to aesthetic color-harmony palette optimization and graphic-layout aesthetics optimization, demonstrating its adaptability to real-world visual art and graphic design problems.

The remainder of this paper is organized as follows. Section 2 describes the structure and details of the basic SBOA. Section 3 presents the proposed MSSBOA, including the three improvement strategies, the overall framework with pseudo-code and flowchart, and the complexity analysis. Section 4 reports the parameter sensitivity analysis, ablation experiments, and a comprehensive comparison on the CEC2017 benchmark functions. Section 5 applies MSSBOA to two visual-design optimization problems. Finally, Section 6 concludes the paper, discusses the limitations of MSSBOA, and outlines future research directions.

## 2. Secretary Bird Optimization Algorithm

SBOA is a population-based metaheuristic inspired by the survival behavior of secretary birds in the African savanna [51]. The algorithm starts from a randomly generated population and improves the candidate solutions through an exploration phase that mimics the three-stage strategy of hunting snakes and an exploitation phase that mimics two strategies of escaping predators. When the stopping criterion is met, SBOA outputs the best solution. The detailed formulation is described as follows.

### 2.1. Initialization

Like most metaheuristics, SBOA initializes a population of N candidate solutions that are uniformly distributed within the search space. Each individual is generated by the following equation.

(1) $$x_{i,j}=lb_{j}+r\cdot \left(ub_{j}-lb_{j}\right),\;i=1,2,\ldots ,N,\;\;j=1,2,\ldots ,D$$

where $x_{i,j}$ denotes the j-th dimension of the i-th individual, r is a random number in $\left[0,1\right]$, $lb_{j}$ and $ub_{j}$ are the lower and upper bounds of the j-th dimension, N is the population size, and D is the dimension of the problem. After initialization, all individuals are evaluated and sorted according to their fitness; a minimization problem is considered in this paper, so individuals with smaller fitness are ranked higher.

### 2.2. Exploration Phase: Hunting Strategy

In the wild, a secretary bird spends a long time searching for prey, such as snakes, before launching a rapid attack. SBOA divides the exploration phase into three successive stages according to the iteration counter. In the first stage (searching for prey), the individual is updated by a differential perturbation of two randomly selected individuals, as in Equation (2).

(2) $$x_{i,j}^{new,P1}=x_{i,j}+\left(x_{rand_{1},j}-x_{rand_{2},j}\right)\cdot R_{1}$$

where $x_{rand_{1},j}$ and $x_{rand_{2},j}$ are the positions of two randomly selected individuals, and $R_{1}$ is a random number in $\left[0,1\right]$. In the second stage (consuming prey), the individual approaches the best individual under a Brownian-motion perturbation, as in Equation (3).

(3) $$x_{i,j}^{new,P1}=X_{best,j}+exp\;\left[{\left(t/T\right)}^{4}\right]\cdot \left(R_{B}-0.5\right)\cdot \left(X_{best,j}-x_{i,j}\right)$$

where $X_{best,j}$ is the j-th dimension of the best individual, t and T are the current and maximum numbers of iterations, and $R_{B}$ is a Brownian-motion random vector drawn from the standard normal distribution. In the third stage (attacking prey), the individual performs a Levy flight around the best individual, as in Equation (4).

(4) $$x_{i,j}^{new,P1}=X_{best,j}+{\left(1-t/T\right)}^{2t/T}\cdot x_{i,j}\cdot R_{L}$$

where $R_{L}=0.5\cdot Levy\left(D\right)$ is a Levy-flight step vector. The three stages are selected by the iteration counter: the first stage is used when t<T/3, the second stage when T/3≤t<2T/3, and the third stage when t≥2T/3. After each update, a greedy selection is performed, as in Equation (5).

(5) $$X_{i}=\left\{\begin{array}{ll} X_{i}^{new}, & f\;\left(X_{i}^{new}\right)<f\;\left(X_{i}\right)\\ X_{i}, & \mathrm{otherwise} \end{array}\right.$$

where $f\left(\cdot \right)$ is the objective function. The greedy selection guarantees that the population never deteriorates.

### 2.3. Exploitation Phase: Escape Strategy

Secretary birds are threatened by predators, such as eagles, and must escape to survive. SBOA models two escape strategies and selects one of them at random in each iteration. When r<0.5, the first strategy $C_{1}$ (camouflage by the environment) is used, as in Equation (6).

(6) $$x_{i,j}^{new,P2}=X_{best,j}+{\left(1-t/T\right)}^{2}\cdot \left(2R_{B}-1\right)\cdot x_{i,j}$$

where r is a random number in $\left[0,1\right]$, and $R_{B}$ is a random vector in $\left[0,1\right]$. Otherwise, the second strategy $C_{2}$ (fleeing toward a safe place) is used, as in Equation (7).

(7) $$x_{i,j}^{new,P2}=x_{i,j}+R_{2}\cdot \left(x_{random,j}-K\cdot x_{i,j}\right)$$

where $R_{2}$ is a random vector in $\left[0,1\right]$, $x_{random,j}$ is a randomly selected individual, and $K=round\left(1+rand\right)\in \{1,2\}$. The Levy flight used in Equation (4) is generated by the Mantegna algorithm [57,58], as in Equation (8).

(8) $$Levy\left(D\right)=\frac{u}{{\left|v\right|}^{1/\beta }},\;\sigma ={\left[\frac{\Gamma \left(1+\beta \right)sin\;\left(\pi \beta /2\right)}{\Gamma \;\left(\frac{1+\beta }{2}\right)\beta \;2^{\left(\beta -1\right)/2}}\right]}^{1/\beta }$$

where $u∼N\left(0,\sigma^{2}\right)$ and $v∼N\left(0,1\right)$ are normally distributed, Γ is the Gamma function, and β=1.5 is a constant.

## 3. Proposed Multi-Strategy Secretary Bird Optimization Algorithm

Although SBOA performs well on many problems, it still has shortcomings that limit its search efficiency. The random initialization cannot guarantee a uniform coverage of the search space, which weakens the initial population diversity. The search relies heavily on the current best individual, so the population is prone to premature convergence and is easily trapped in local optima, and the transition between exploration and exploitation is not fully balanced. To overcome these limitations, this section proposes a multi-strategy SBOA (MSSBOA) that incorporates three improvement strategies: good point set initialization, lens opposition-based learning, and adaptive Cauchy–Gaussian mutation. Each strategy is described in detail below, followed by the overall framework, the pseudo-code, and the complexity analysis.

### 3.1. Good Point Set Initialization (GPSI)

The distribution of the initial population strongly affects the convergence of a metaheuristic. A uniformly distributed initial population enhances diversity and enables the algorithm to explore the solution space more comprehensively. The good point set, derived from number theory [59], is a low-discrepancy sequence whose deviation is asymptotically lower than that of pseudo-random sampling, and it has been successfully used to initialize swarm algorithms [60]. For a D-dimensional problem, we let p be the smallest prime satisfying $\left(p-3\right)/2\geq D$. The good point is constructed by Equation (9).

(9) $$r_{k}=\left\{2cos\;\left(\frac{2\pi k}{p}\right)\right\},\;k=1,2,\ldots ,D$$

where {⋅} denotes the fractional part. The i-th individual is then mapped to the search space by Equation (10).

(10) $$x_{i,j}=lb_{j}+\left\{r_{j}\cdot i\right\}\cdot \left(ub_{j}-lb_{j}\right),\;i=1,2,\ldots ,N$$

Compared with random initialization, the good point set yields a more even spread of the initial population that is independent of the dimension, providing the algorithm with a better starting point for the subsequent search.

### 3.2. Lens Opposition-Based Learning (LOBL)

Opposition-based learning evaluates a candidate solution together with its opposite and keeps the better one, which effectively enlarges the explored region [61,62]. Lens opposition-based learning extends this idea using the principle of convex-lens imaging: an object placed on one side of a lens forms an image on the other side, which corresponds to a refracted reverse solution with a tunable scaling factor [63]. The lens-refracted solution is computed by Equation (11).

Lens opposition-based learning replaces the reflection of standard opposition-based learning with the image that a thin convex lens forms of the current individual, and the derivation below makes the correspondence between the optical construction and Equation (11) explicit.

**Figure 2:**
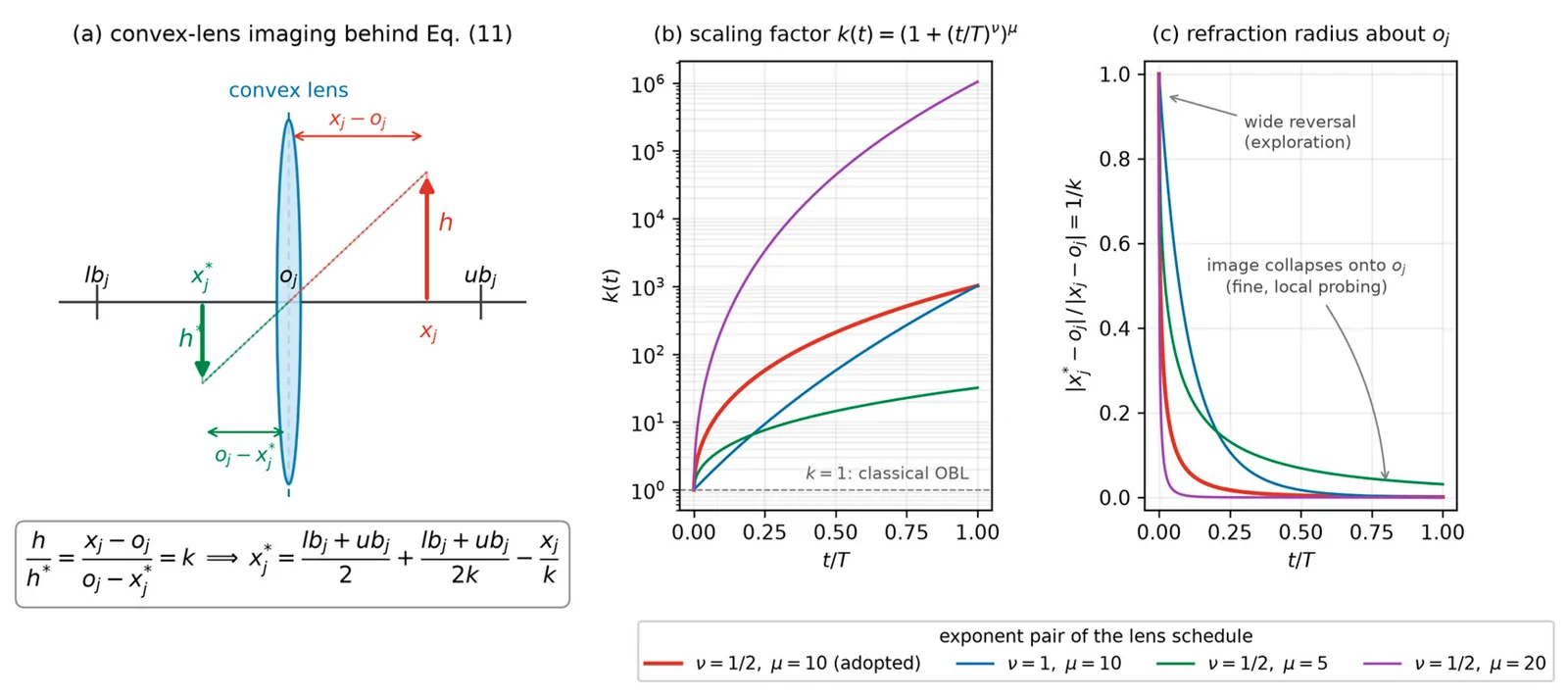
Convex-lens imaging behind the lens opposition-based learning strategy: (**a**) the similar-triangle construction that yields Equation (11), in which the scaling factor is the ratio of object height to image height; (**b**) the dynamic scaling factor of Equation (12) for several exponent pairs; and (**c**) the resulting refraction radius about the interval midpoint.

By considering one coordinate axis of the search space, the j-th, as restricted to the interval between ${lb}_{j}$ and ${ub}_{j}$, we let $o_{j}=\frac{{lb}_{j}+{ub}_{j}}{2}$ denote its midpoint. In the optical analogy, the midpoint plays the role of the optical center: a thin convex lens is placed at $o_{j}$ with its principal axis perpendicular to the coordinate axis, as illustrated in Figure 2a. The current individual is modeled as an object of height h whose base stands on the axis at $x_{j}$, and the lens forms an inverted real image of height $h^{*}$ whose base stands at ${x_{j}}^{*}$ on the opposite side of the center.

The ray through the optical center is undeviated, so the object triangle and the image triangle share a pair of vertically opposite angles at $o_{j}$ and are similar. Comparing corresponding sides gives the single geometric relation on which the operator rests:

$$\frac{h}{h^{*}}=\frac{x_{j}-o_{j}}{o_{j}-{x_{j}}^{*}}=k$$

The tunable scaling factor is therefore defined as the ratio of object height to image height, $k=\frac{h}{h^{*}}$. It is not a free numerical constant but the reciprocal of the transverse magnification of the virtual lens, which is the one physical lens parameter the operator exposes. Solving the similarity relation for the image position gives

$${x_{j}}^{*}=o_{j}+\frac{o_{j}-x_{j}}{k}$$

which, after writing the midpoint out in terms of the bounds, is exactly Equation (11). By setting $k=1\Rightarrow {x_{j}}^{*}={lb}_{j}+{ub}_{j}-x_{j}$, the lens formulation contains classical opposition-based learning as its unit-magnification special case.

The behavior of the operator follows directly from the derivation. Equation (11) implies $\left|{x_{j}}^{*}-o_{j}\right|=\frac{\left|x_{j}-o_{j}\right|}{k}$, so the scaling factor controls the radius at which the refracted solution is placed about the midpoint of the interval, not its distance from the current individual. A small value produces a wide reversal that lands far from the parent and probes the opposite side of the domain, which is the behavior an exploratory operator should have early in a run; a large value contracts the image onto the neighborhood of the center, so that the refracted point becomes a fine, low-variance probe rather than a large jump. Figure 2c plots this contraction ratio over the course of a run. We took the opportunity to correct a statement in the submitted version, which described a large scaling factor as keeping the refracted solution close to the current individual; as the derivation shows, it keeps the image close to the midpoint.

(11) $$x_{j}^{*}=\frac{lb_{j}+ub_{j}}{2}+\frac{lb_{j}+ub_{j}}{2k}-\frac{x_{j}}{k}$$

where $x_{j}^{*}$ is the refracted solution, and k is an adjustment factor. When k=1, Equation (11) degenerates into the standard opposition-based learning. To balance the global and local search, a dynamically increasing factor is adopted, as in Equation (12).

(12) $$k={\left(1+{\left(\frac{t}{T}\right)}^{1/2}\right)}^{10}$$

Accordingly, a small k in the early stage places the refracted solution far from the interval midpoint, on the opposite side of the domain from the current individual, which favors exploration; a large k in the later stage contracts the image onto the neighborhood of the midpoint, which turns the operator into a fine-grained local probe and favors exploitation. In MSSBOA, the lens opposition-based learning is applied only to the inferior individuals—that is, to the worst $N_{min}=0.2\;N$ members of the population—at the end of each iteration; the value 0.2 N is the one selected by the grid search of Section 4.2. As illustrated in Figure 2, restricting the operator to the inferior subpopulation lets the population escape local optima while the greedy selection protects the elite individuals.

The functional form of Equation (12), $k\left(t\right)={\left(1+{\left(\frac{t}{T}\right)}^{\nu }\right)}^{\mu }$, is fixed by three structural requirements and one grid search. First, the factor equals one at the start of the run for any exponent pair, so the operator begins as exact opposition-based learning while the population is still uninformative. Second, it must increase monotonically, so that the refraction radius contracts without oscillating. Third, it must grow by several orders of magnitude, because the contraction ratio is its reciprocal; the exponent supplies this range cheaply, since $k\left(T\right)=2^{\mu }$.

Within these constraints, the two exponents are free and were selected empirically rather than derived. The root governs where the contraction happens, as a linear root spreads it evenly over the run, while a square root frontloads it, so the aggressive reversal is concentrated in the early iterations during which the hunting strategy of SBOA is itself exploratory (Figure 2b). The power sets the terminal contraction, which must be small enough for the refracted point to act as a local probe but not so small that the operator degenerates into re-evaluating the midpoint. Section 4.2 reports a grid over both exponents, including the constant that reduces the operator to plain opposition-based learning.

### 3.3. Adaptive Cauchy–Gaussian Mutation (ACGM)

To further balance exploration and exploitation and to help the best individual escape local optima, an adaptive Cauchy–Gaussian mutation is imposed on the best individual [54,64]. The Cauchy distribution has heavier tails than the Gaussian distribution and therefore generates larger perturbations for global exploration, whereas the Gaussian distribution produces small perturbations for local refinement. The mutation is defined by Equation (13).

(13) $$X_{best}^{new}=X_{best}\cdot \left(1+\lambda_{1}\;Cauchy\left(0,1\right)+\lambda_{2}\;N\left(0,1\right)\right)$$

where $Cauchy\left(0,1\right)$ is a standard Cauchy random number, $N\left(0,1\right)$ is a standard normal random number, and $\lambda_{1}$ and $\lambda_{2}$ are adaptive weights defined by Equation (14).

(14) $$\lambda_{1}=1-\frac{t^{2}}{T^{2}},\;\;\lambda_{2}=\frac{t^{2}}{T^{2}}$$

In the early iterations, $\lambda_{1}$ is large, so the Cauchy-dominated perturbation enhances global exploration; in the later iterations, $\lambda_{2}$ becomes large, so the Gaussian-dominated perturbation strengthens local exploitation. The mutated solution replaces the best individual only when it is better to do so, according to the greedy rule in Equation (5).

The adaptive weights of Equation (14) are $\lambda_{1}=1-\frac{t^{2}}{T^{2}},\lambda_{2}=\frac{t^{2}}{T^{2}}$, so the perturbation is Cauchy-dominated while the search is still explorative and Gaussian-dominated once it has converged; the heavy tail of the Cauchy distribution supplies the occasional long jump that lets the elite leave a basin, and the Gaussian term supplies the short steps that refine it.

### 3.4. The Framework of MSSBOA

**Figure 3:**
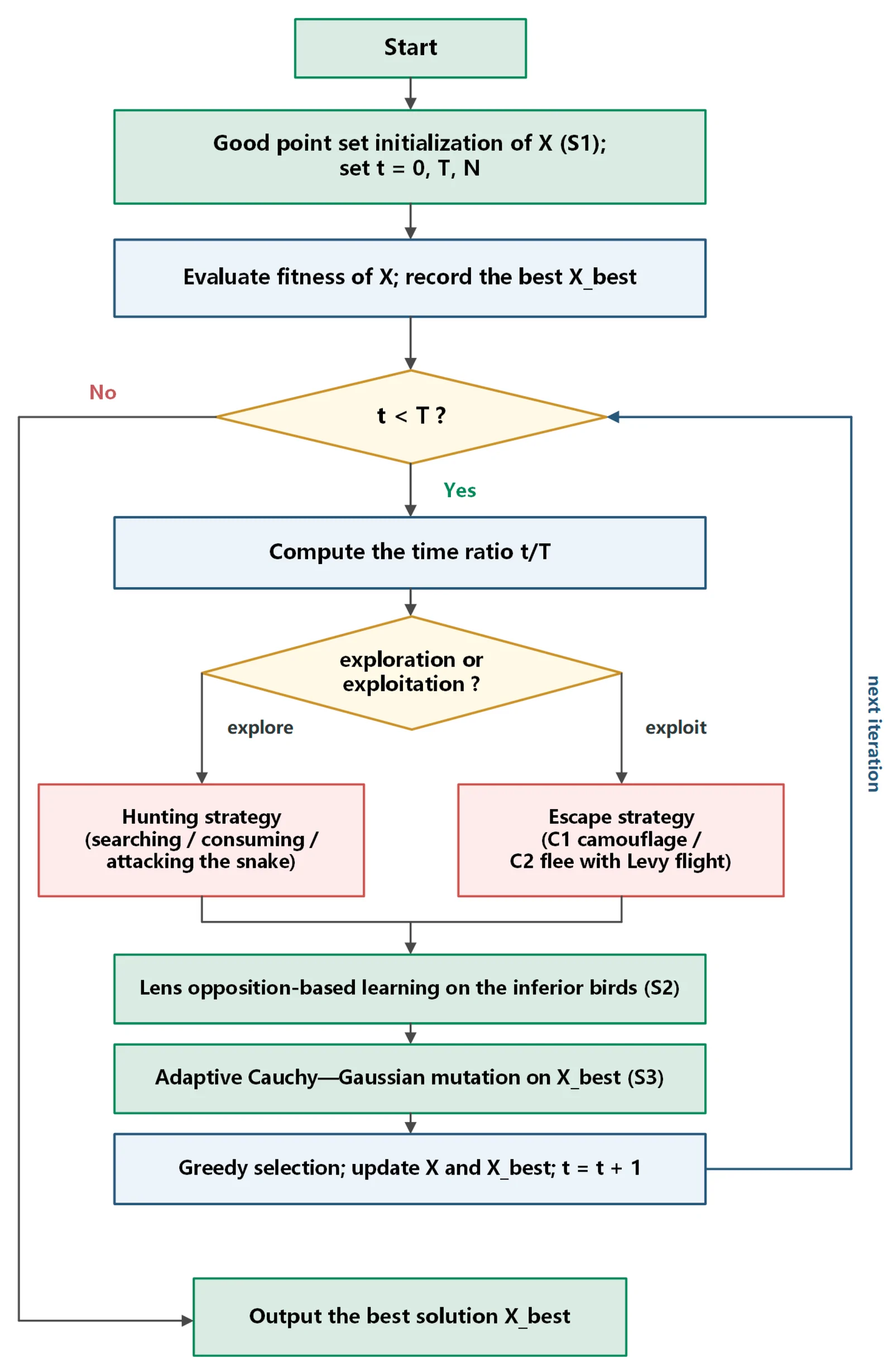
The flowchart of MSSBOA.

MSSBOA is obtained by integrating the three improvement strategies into the basic SBOA. The good point set initialization (GPSI) generates a high-quality initial population; the hunting and escape strategies of SBOA drive the main search; the lens opposition-based learning (LOBL) refreshes the inferior individuals to escape local optima; and the adaptive Cauchy–Gaussian mutation (ACGM) perturbs the best individual to balance exploration and exploitation. The pseudo-code of MSSBOA is given in Algorithm 1, and the corresponding flowchart is shown in Figure 3.
**Algorithm 1.** Multi-Strategy Secretary Bird Optimization Algorithm (MSSBOA)1:  Initialize population X using good point set (Equation (9) and Equation (10))  // **GPSI**2:  Set t = 0, maximum iteration T, population size N3:  Evaluate fitness of X and record the best individual X_best4:  while t < T do5:    for each individual i do6:      if t < T/3 then   update X_i by Equation (2)     // searching for prey7:      else if t < 2T/3 then   update X_i by Equation (3)  // consuming prey8:      else   update X_i by Equation (4)           // attacking prey9:      end if; perform greedy selection by Equation (5)10:       if r < 0.5 then   update X_i by Equation (6)     // escape C111:       else   update X_i by Equation (7)          // escape C212:       end if; perform greedy selection by Equation (5)13:    end for14:    Apply lens opposition-based learning to the worst $N_{min}=0.2\;N$ individuals by Equation (11) and Equation (12) // **LOBL**15:    Apply adaptive Cauchy–Gaussian mutation to X_best by Equation (13) and Equation (14)  // **ACGM**16:    Update X_best; t = t + 117:  end while18:  return the best individual X_best

### 3.5. Complexity Analysis of MSSBOA

The computational complexity of MSSBOA is analyzed in terms of time and space complexity. We let N be the population size, D the dimension, and T the maximum number of iterations. According to the original literature, the time complexity of the basic SBOA is $O\left(N\cdot D\cdot T\right)$ and its space complexity is $O\left(N\cdot D\right)$.

For MSSBOA, the good point set initialization has the same complexity as random initialization, namely $O\left(N\cdot D\right)$, and therefore does not change the order of complexity. The lens opposition-based learning is applied to a fixed fraction of the population, with complexity $O\left(N\cdot D\right)$ per iteration, and the adaptive Cauchy–Gaussian mutation acts only on the best individual with complexity $O\left(D\right)$. Hence the overall time complexity of MSSBOA remains $O\left(N\cdot D\cdot T\right)$, the same as the basic SBOA. Regarding space, MSSBOA only stores the population matrix and a few temporary vectors for the refracted and mutated solutions, so its space complexity is still $O\left(N\cdot D\right)$. In summary, the three improvement strategies enhance the performance of SBOA without increasing its order of computational complexity.

### 3.6. Novelty of the Proposed Integration Framework

The three operator families used above are established techniques, and their individual novelty is not claimed here. Good point set initialization has been applied to many swarm algorithms, lens opposition-based learning has been combined with, among others, sand cat swarm optimization and whale optimization, and Cauchy–Gaussian mutation is a standard device for rebalancing exploration and exploitation; a closely related combination of opposition-based learning with Cauchy mutation, velocity clamping, and a mirror operator has recently been reported for the ideal gas molecular movement algorithm [57]. The contribution of this paper is therefore the integration framework: how each operator is re-specified for SBOA, how its control parameter is adapted, and how the three are assigned to disjoint parts of the population so that they reinforce rather than duplicate one another. Table 1 states these differences explicitly against representative existing applications of the same operators.

Three design decisions distinguish the present framework. First, the refraction and the mutation act on disjoint subsets; LOBL is applied only to the inferior individuals and ACGM only to the elite, so that the diversity-restoring operator can never displace the best solution and the intensification operator can never be wasted on individuals that the greedy selection is about to discard. Most existing applications apply opposition-based learning to the whole population and mutation to a randomly chosen individual, which lets the two interfere. Second, the lens factor is a schedule derived from the imaging geometry and tied to the iteration budget rather than a constant chosen by hand, and its two exponents are exposed and tuned (Section 4.2). Third, the operators are placed at the points of the SBOA iteration where its own three-stage hunting strategy is weakest; refraction is applied after the escape phase, when the population has just contracted towards the elite, and mutation is applied to the elite itself, which is the single individual that both SBOA phases depend on and that neither can perturb.

## 4. Numerical Experiments Using the CEC2017 Test Suite

In this section, the performance of MSSBOA was comprehensively examined on the CEC2017 benchmark suite. Section 4.1 describes the experimental setting and the CEC2017 functions. Section 4.2 analyzes the sensitivity of the two key parameters of MSSBOA. Section 4.3 reports the ablation experiments that verify the effectiveness of the three improvement strategies. Finally, Section 4.4 provides a comprehensive comparison between MSSBOA and nine state-of-the-art algorithms, as analyzed by the Friedman test, the Nemenyi post hoc test, and the Wilcoxon rank-sum test.

### 4.1. Experiment Setting and Performance Metrics

All experiments were carried out on a workstation with an AMD Ryzen 9 7945HX 2.5 GHz CPU and 32 GB of memory, running Windows 11 and MATLAB R2023a. The CEC2017 test suite [65] contains 29 test functions (F2 is excluded because of its unstable behavior), in which F1 and F3 are unimodal functions, F4–F10 are simple multimodal functions, F11–F20 are hybrid functions, and F21–F30 are composition functions. The unimodal functions evaluate the convergence accuracy of an algorithm, the multimodal functions test the ability to escape local optima, and the hybrid and composition functions assess the balance between exploration and exploitation on complex landscapes. The search range was ${\left[-100,100\right]}^{D}$ and the dimensions were set to 10, 30, 50, and 100. To ensure a fair comparison, the maximum number of function evaluations was used as the stopping criterion and was set to 10,000×D. Each algorithm was run 30 times independently, and the best value, the mean value and the standard deviation were recorded. MSSBOA was compared with the basic SBOA [51] and nine advanced algorithms, including GWO [42], SCA [34], SMA [47], COA [44], GKSO [49], MRFO [66], RIME [67], QIO [68], and LSHADE [69]. The parameter settings of all algorithms are listed in Table 2 and are taken from the original literature of each.

### 4.2. Parameter Sensitivity Analysis

**Figure 4:**
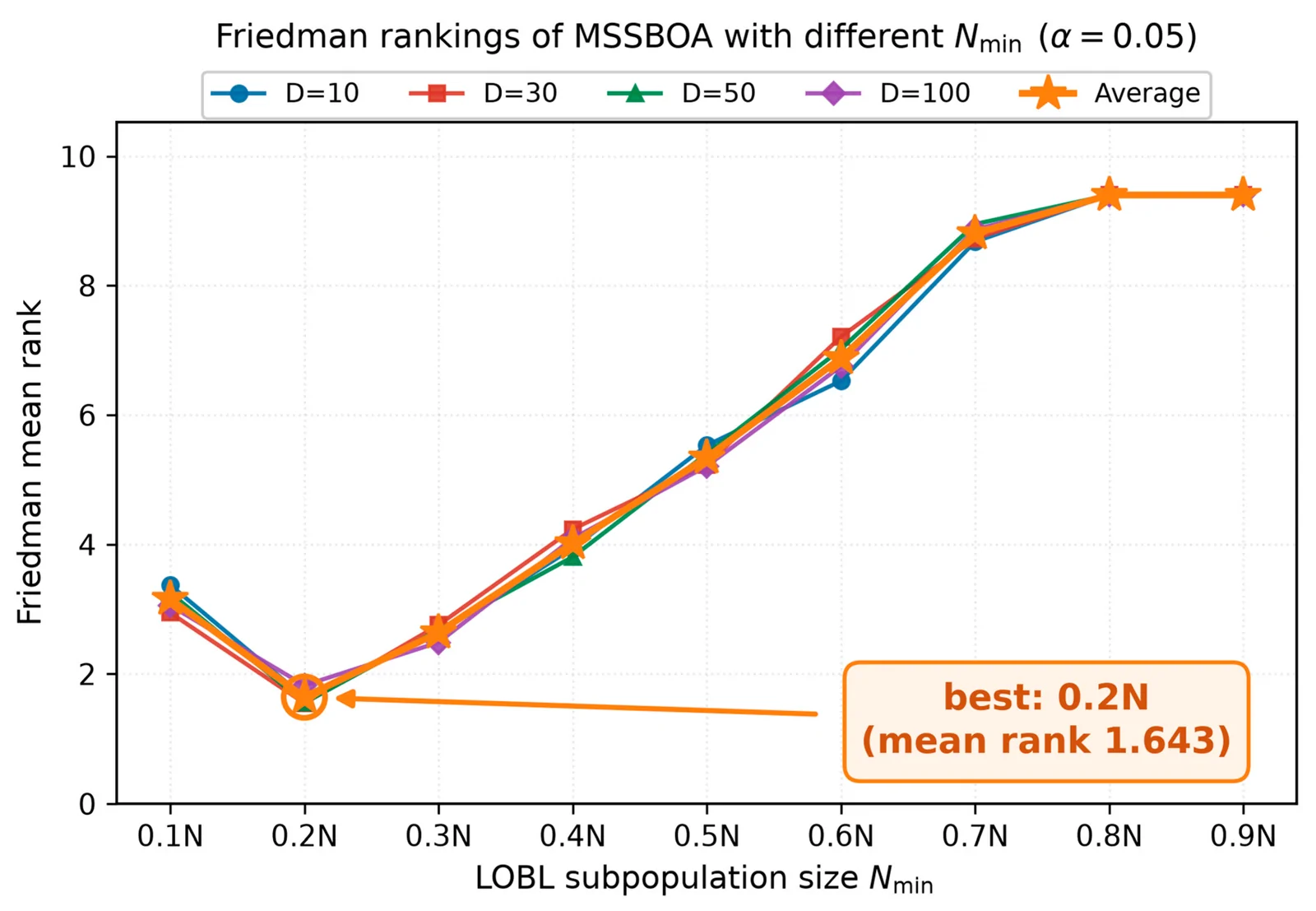
Friedman rankings of MSSBOA with different $N_{min}$ (α=0.05). The best setting is marked above the corresponding point.

**Figure 5:**
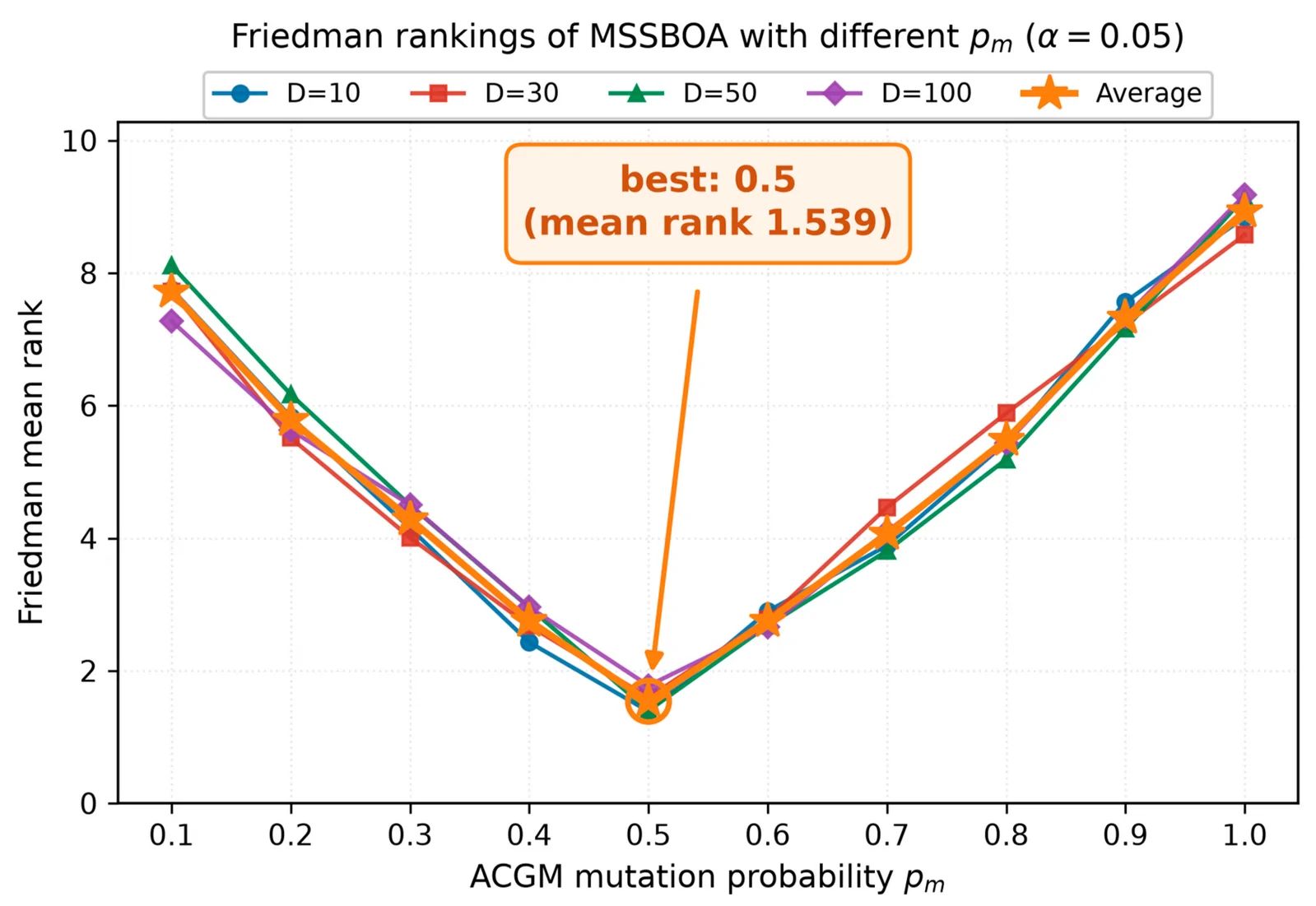
Friedman rankings of MSSBOA with different $p_{m}$ (α=0.05). The best setting is marked above the corresponding point.

Two parameters strongly affect the performance of MSSBOA: the minimum population factor $N_{min}$ used in the lens opposition-based learning and the mutation probability $p_{m}$ of the adaptive Cauchy–Gaussian mutation. A grid search was conducted on the CEC2017 suite over 30 independent runs. $N_{min}$ was varied from 0.1 N to 0.9 N with a step of 0.1 N, and $p_{m}$ was varied from 0.1 to 1.0 with a step of 0.1. Table 3 and Table 4 report the Friedman rankings (significance level α=0.05) for $N_{min}$ and $p_{m}$, respectively, where the best ranking is highlighted in bold. The corresponding rankings are visualized in Figure 4 and Figure 5.

As shown in Table 3 and Figure 4, MSSBOA achieves the best average ranking when $N_{min}=0.2\;N$. As $N_{min}$ increases, the ranking gradually deteriorates because too large a refraction subpopulation disturbs the elite individuals. Therefore, $N_{min}=0.2\;N$ is adopted in this paper.

For the mutation probability, Table 4 and Figure 5 show that MSSBOA performs best when $p_{m}=0.5$, which provides a suitable balance between perturbing the best individual and preserving its information. Consequently, the parameter settings of MSSBOA are $N_{min}=0.2\;N$ and $p_{m}=0.5$.

Four further quantities were fixed rather than tuned in the submitted version, and a reviewer rightly asked why. Three of them are inherited from the base algorithm and were deliberately left untouched so that the ablation of Section 4.3 measures the added operators and not a re-tuning of SBOA, which were: the population size N, which is common to every algorithm in Table 2 and is fixed for fairness rather than for performance; the Levy stability index β=1.5, which is the value used in the original SBOA and, before it, the standard value for the Mantegna formulation; and the three-stage time division T/3,2T/3 of the hunting strategy. The fourth quantity, the exponent pair of the lens schedule, belongs to this paper and is examined below.

Each was varied while the remainder of the configuration was held as fixed on the CEC2017 suite in 10 and 30 dimensions over 30 independent runs, and the settings are compared by the Friedman mean rank within each grid.

For the population size N, the best mean rank is 2.190 at N=30, which is the adopted setting over a spread of 1.603 across the grid; for the Levy stability index β, the best mean rank is 2.414 at β=1.1, against 2.448 for the adopted β=1.5, which places it third of four at a distance of 0.034 on a spread of 0.276; for the hunting-stage time division, the best mean rank is 2.310 at T/4,3T/4, against 2.759 for the adopted T/3,2T/3, which places it fourth out of four at a distance of 0.448 on a spread of 0.448; and for the lens scaling schedule, the best mean rank is 2.672 at k≡1 (classical OBL), against 2.793 for the adopted ν=1/2, μ=10, which places it second of five at a distance of 0.121 on a spread of 0.707. The adopted setting is within 0.15 of the best one for population size N, Levy stability index β, and lens scaling schedule, and further from it for hunting-stage time division. We report this as it was given rather than claim that every inherited value is optimal; what the grids show is that the differences are of the order of a fraction of a rank position, not that the settings could not be improved.

The lens schedule is the one parameter of the four that belongs to this paper, and its grid separates the two exponents. The two settings that move the power μ away from 10 are the worst two of the five, which is what the derivation predicts: μ fixes the terminal contraction $k\left(T\right)=2^{\mu }$, so it determines whether the operator ends the run refracting into a neighborhood of the interval midpoint or barely contracting at all. However, between the adopted pair and the constant k=1, the mean ranks differ by only 0.121 in favor of plain opposition-based learning, so that at these dimensions, the schedule earns its place through the scale of the contraction rather than by beating exact opposition outright.

Besides the two key parameters analysed above, MSSBOA inherits four further settings from the basic SBOA and from the operators introduced in Section 3: the population size *N*, the stability index *β* of the Levy flight, the time division that separates the three hunting stages, and the exponent pair (*ν*, *μ*) of the lens scaling schedule of Equation (12). Each was varied on the CEC2017 suite over 30 independent runs at *D* = 10 and *D* = 30, with all remaining settings held fixed, and the Friedman mean ranks (*α* = 0.05) are reported in Table 5 for the population size, Table 6 for the stability index, Table 7 for the hunting-stage time division and Table 8 for the lens scaling schedule. A lower value indicates a better rank.

### 4.3. Ablation Experiment

**Figure 6:**
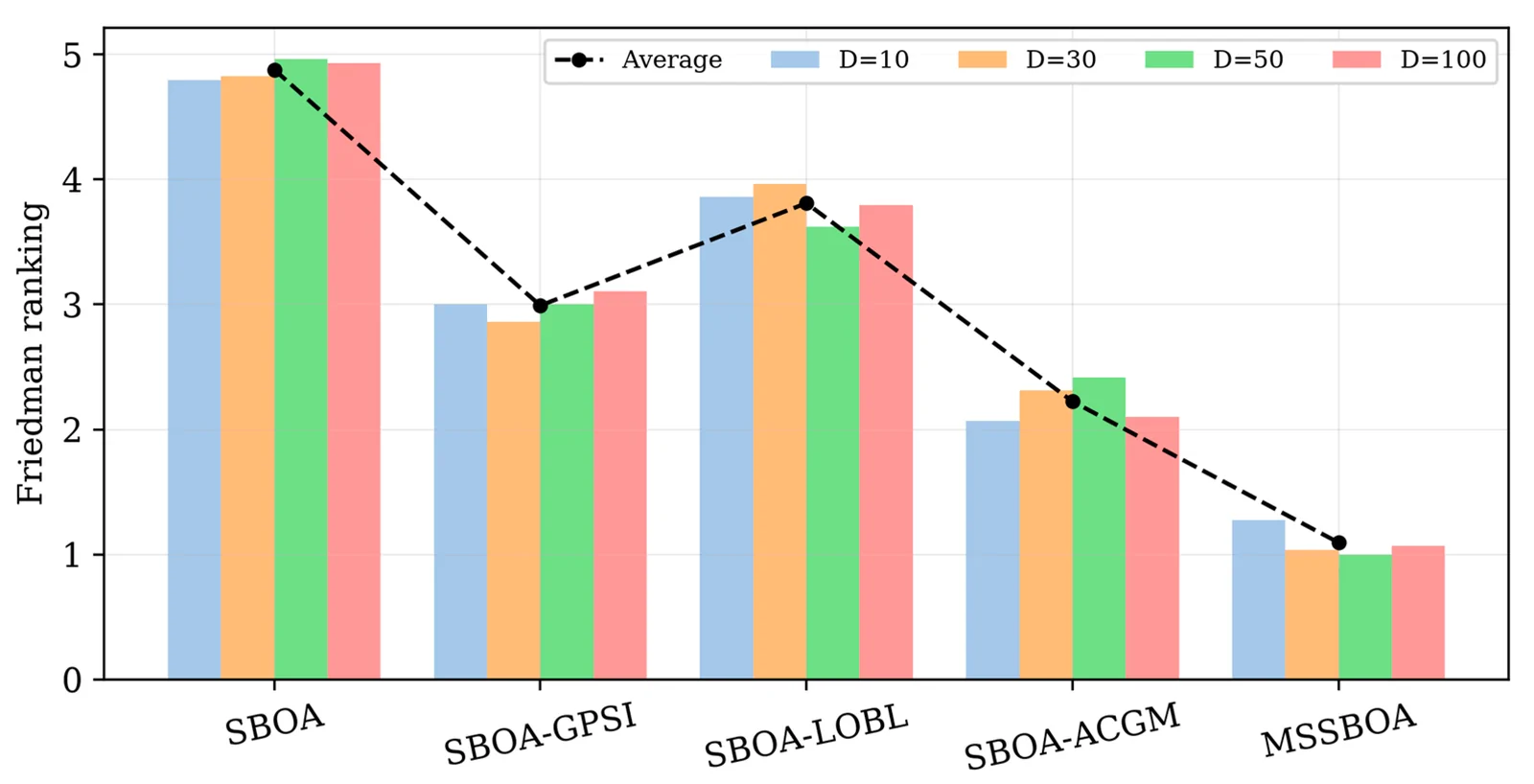
Friedman rankings of MSSBOA with different strategies (α = 0.05).

To investigate the contribution of each improvement strategy, three variants of MSSBOA were constructed by adding a single strategy to the basic SBOA, namely SBOA-GPSI, SBOA-LOBL, and SBOA-ACGM, as listed in Table 9. The four algorithms and the full MSSBOA were run 30 times independently on the CEC2017 suite in four dimensions, and the results were analyzed by the Friedman test. The complete statistical results are reported in Table A1, Table A2, Table A3 and Table A4 of Appendix A. Table 10 summarizes the Friedman rankings, which are visualized in Figure 6.

According to Table 10 and Figure 6, all *p*-values are far below 0.05, indicating significant differences among the five algorithms. MSSBOA ranks first in all four dimensions with an overall average ranking of 1.095, followed by SBOA-ACGM, SBOA-GPSI, and SBOA-LOBL, while the basic SBOA ranks last. Each single-strategy variant outperforms the basic SBOA, which indicates that each strategy contributes. Among them, the adaptive Cauchy–Gaussian mutation (ACGM) contributes the most, because it directly helps the best individual escape local optima and balances exploration and exploitation; the good point set initialization (GPSI) ranks next by enriching the initial diversity; and the lens opposition-based learning (LOBL) also improves performance by refreshing inferior individuals. The full MSSBOA outperforms every single-strategy variant, demonstrating that the three strategies complement and reinforce each other.

The scope of this ablation should be stated precisely. Table 9 and Table 10 and Figure 6 isolate each strategy by adding it alone to the basic SBOA so that what they measure is the individual contribution of GPSI, LOBL, and ACGM against a common baseline. They do not measure the four intermediate pairings, and no claim is made here about them.

The question of why the three strategies are used together is therefore answered by the structure of the framework rather than by enumeration. As Section 3.6 sets out, the operators act on disjoint parts of the population and at different points of the iteration: refraction is confined to the inferior subpopulation, mutation to the elite, and the good point set acts once before either of them are run. Because their domains do not overlap, no operator can undo the work of another, which is the mechanism by which the combination is expected to be more than the sum of its parts. A full $2^{3}$ factorial design over the eight combinations would settle the point empirically and is left to future work.

### 4.4. Comparison with Other Advanced Algorithms

**Figure 7:**
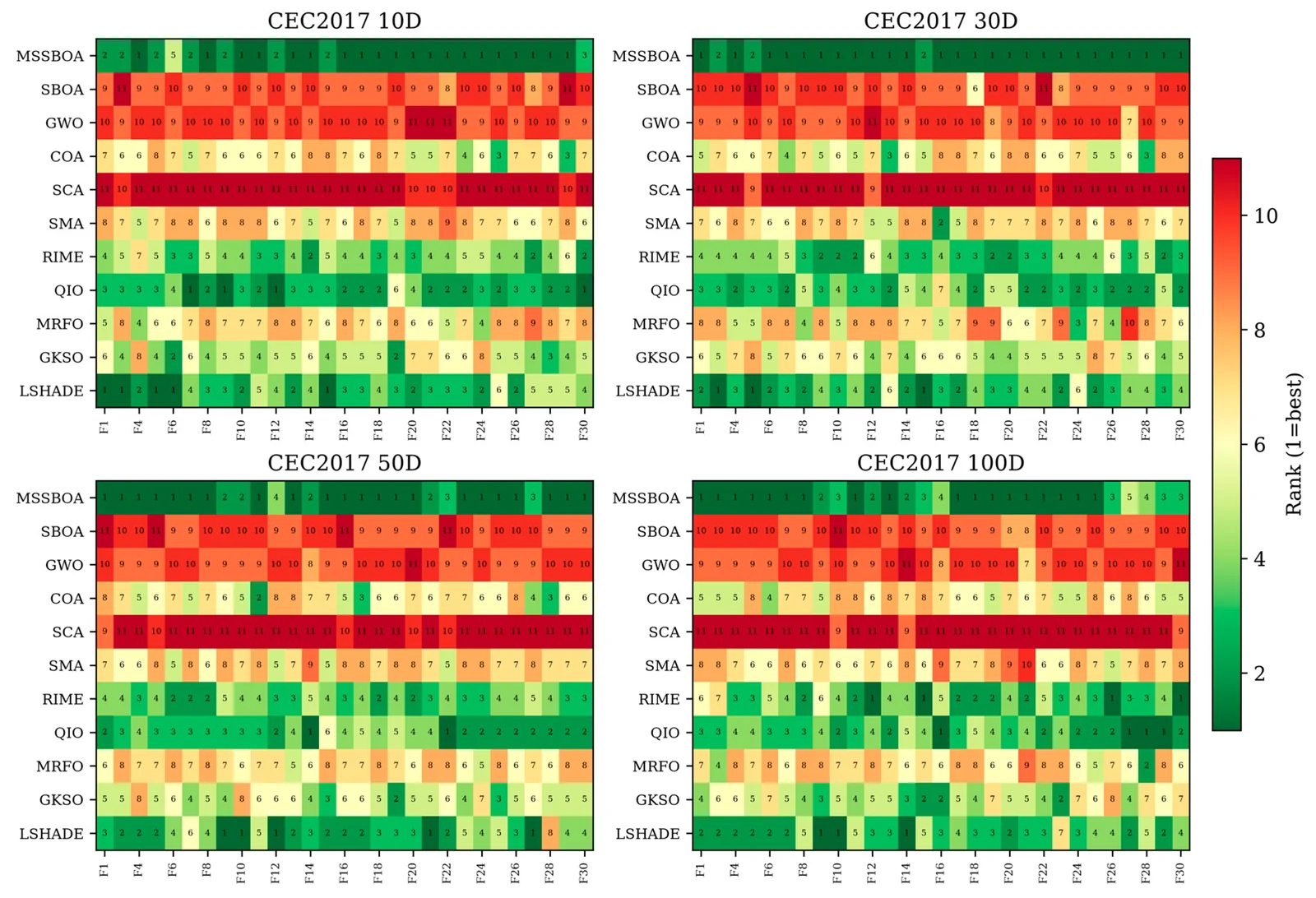
Ranking heatmaps of MSSBOA and the comparison algorithms on the CEC2017 suite.

In this subsection, MSSBOA was compared with the basic SBOA and nine advanced algorithms on the CEC2017 suite in 10, 30, 50 and 100 dimensions. The complete results, including the best value, mean value, standard deviation, and rank, are reported in Table A5, Table A6, Table A7 and Table A8 of Appendix A. The ranking of each algorithm on every function is first visualized as heatmaps in Figure 7.

**Figure 8:**
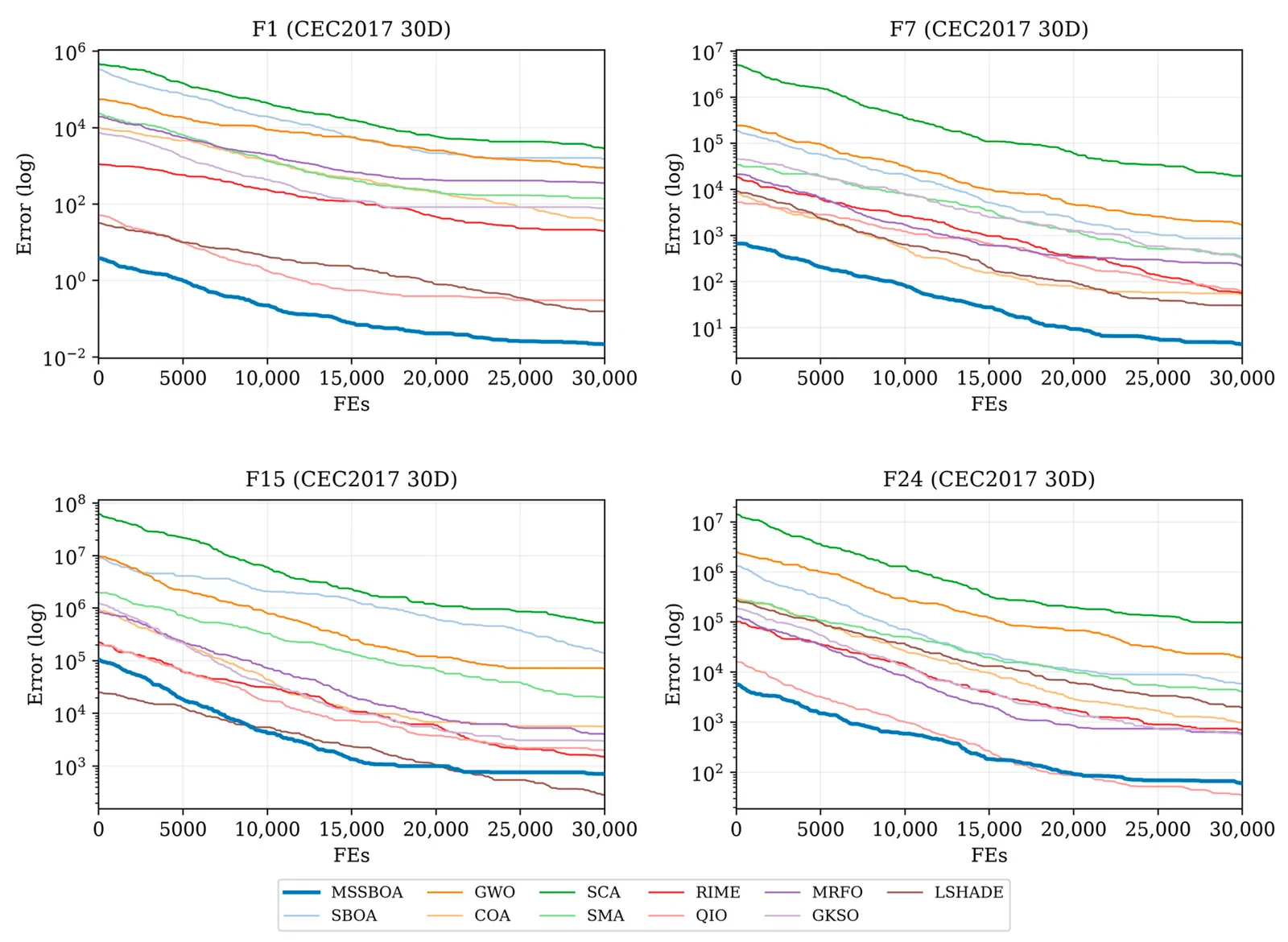
Convergence curves of MSSBOA and the comparison algorithms on representative CEC2017 functions (30D).

As shown in Figure 7, MSSBOA ranks first on 20 functions in 10D, 26 functions in 30D, 22 functions in 50D, and 18 functions in 100D; that is, on more than half of the 29 functions in every dimension, which preliminarily demonstrates its superiority. The convergence curves of representative functions are illustrated in Figure 8, where MSSBOA converges faster and reaches lower error values than the comparison algorithms.

**Figure 9:**
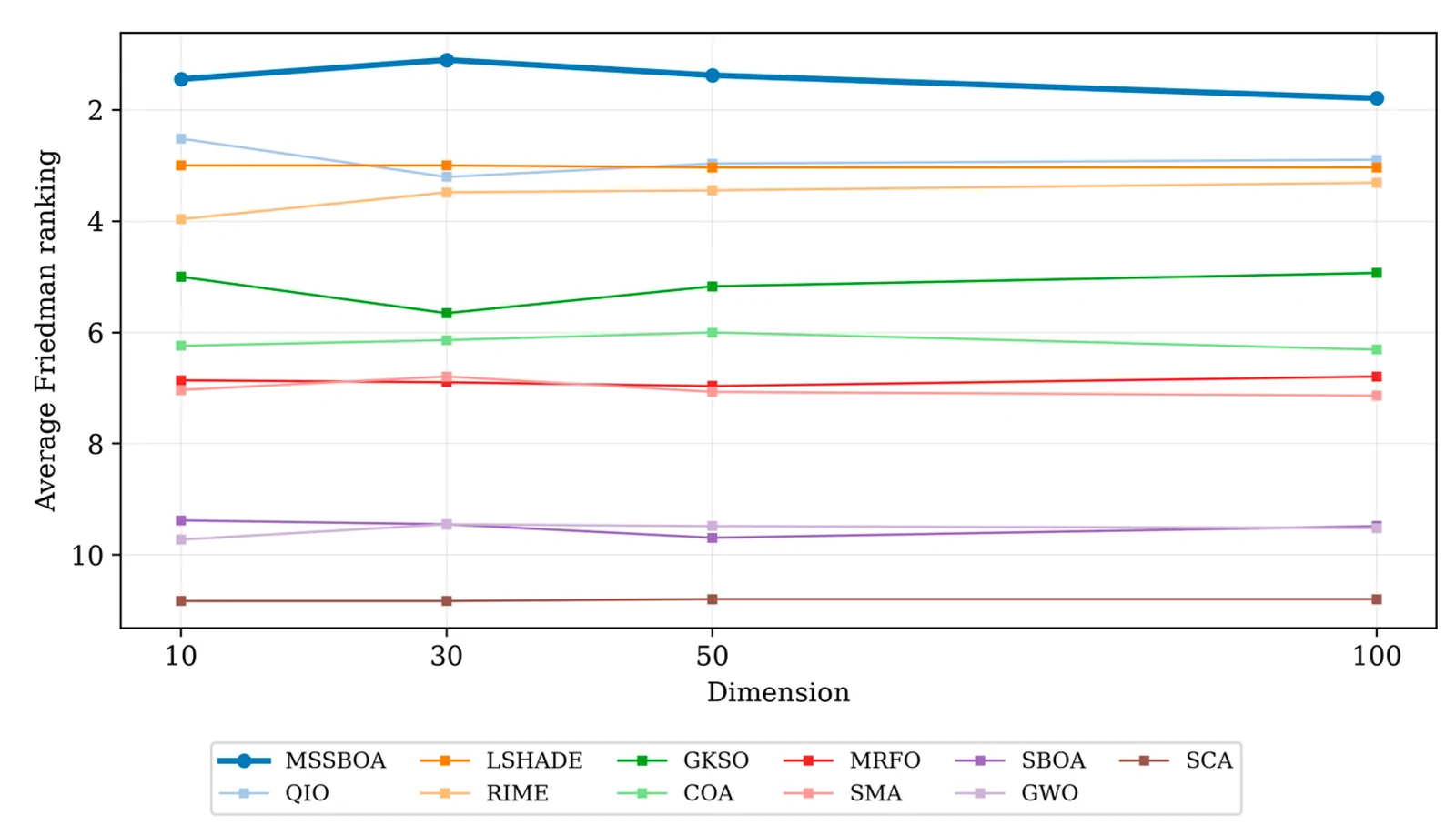
Friedman rankings of MSSBOA and the comparison algorithms across dimensions (α = 0.05).

To statistically analyze the overall performance, the Friedman test was performed; the results are summarized in Table 11 and visualized in Figure 9.

**Figure 10:**
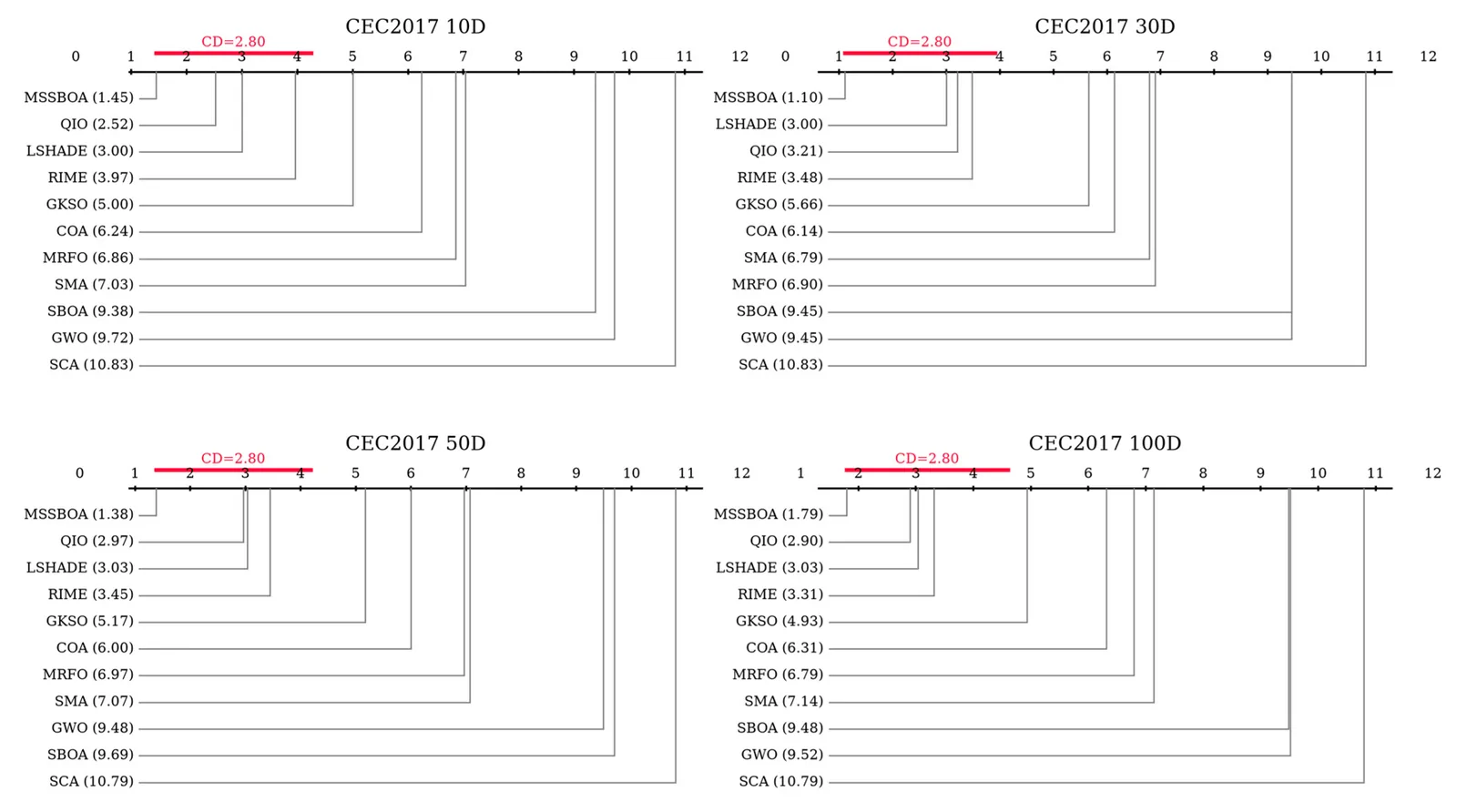
Nemenyi post hoc test of MSSBOA and the comparison algorithms.

As shown in Table 11 and Figure 9, all Friedman *p*-values are below 0.05, confirming significant differences among the algorithms. MSSBOA achieves the best average ranking of 1.431 and ranks first in all four dimensions, whereas the basic SBOA ranks near the bottom; the overall order is approximately MSSBOA > QIO > LSHADE > RIME > GKSO > COA > MRFO > SMA > SBOA > GWO > SCA. This clearly reflects the substantial improvement of MSSBOA over the basic SBOA. To further quantify the pairwise differences, the Nemenyi post hoc test was applied, as shown in Figure 10, where algorithms connected by a line are not significantly different. MSSBOA is located at the leftmost position and is significantly superior to most comparison algorithms.

**Figure 11:**
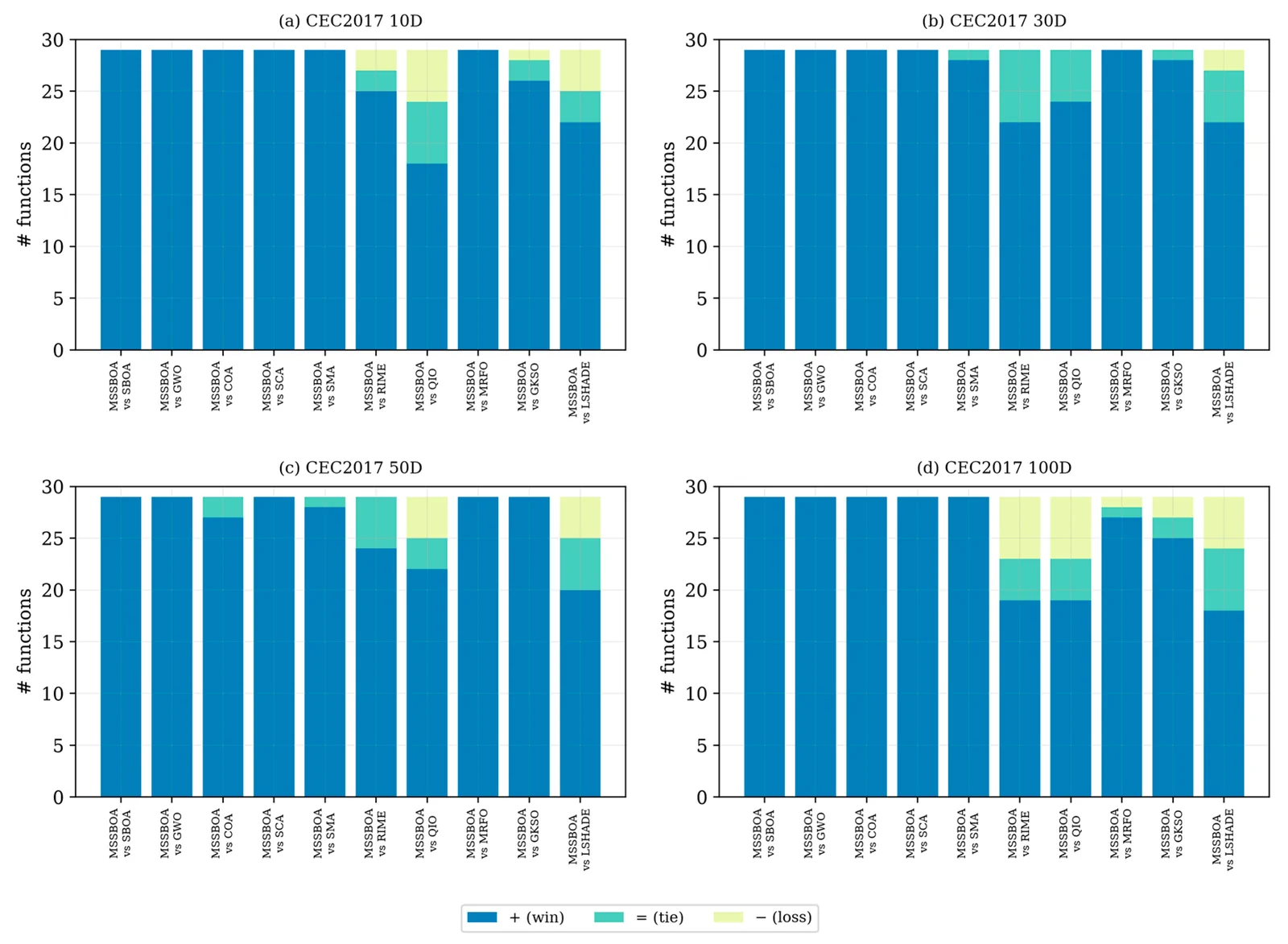
Visualization of the Wilcoxon rank-sum test results of MSSBOA and the comparison algorithms (α = 0.05).

Finally, the Wilcoxon rank-sum test was conducted to compare MSSBOA with each algorithm on every function. Table 12 reports the statistics, where ‘+’, ‘=’, and ‘−’ denote the numbers of functions on which MSSBOA is significantly better than, similar to, and worse than the comparison algorithm, respectively. The results are visualized in Figure 11.

The statistical picture deserves a more balanced reading than a count of wins. Against the weaker comparison algorithms, the outcome is unambiguous—MSSBOA wins all 116 cases against the basic SBOA, GWO, and SCA—but against the two strongest competitors, it is not. MSSBOA loses 15 of 116 cases to QIO and 15 of 116 to LSHADE, that is 12.9% of the comparisons in each case, and a further 18 and 19 cases are statistically indistinguishable. Taken together, MSSBOA is significantly better than QIO on 71.6% of the cases and LSHADE on 70.7%, so on roughly three cases in ten, these two algorithms are at least as good as the proposed method. This is a real limitation and not a rounding error.

Table 13 breaks the losses down by function class, using the per-function ranks of Table A5, Table A6, Table A7 and Table A8. The losses are not distributed uniformly. Against QIO, they concentrate on the composition functions F21–F30, which account for eight of the 16 rank losses; however, composition functions supply only 40 of the 116 cases, and none occur on a unimodal function. Against LSHADE, the profile is the opposite, as 10 of the 20 rank losses fall on the unimodal and simple multimodal functions F1–F10, which is consistent with the linear population size reduction and archive of LSHADE being particularly effective on landscapes with a single dominant basin. The pattern indicates a specific weakness rather than a general one, where the elite-guided intensification of MSSBOA pays off on hybrid landscapes but is less effective when a problem has many equally deep basins whose relative depth only becomes apparent late in the run, which is precisely the structure of the CEC2017 composition functions.

The dimensional trend confirms this reading. The mean rank gap between MSSBOA and QIO falls monotonically from 2.103 at 30 dimensions to 1.103 at 100 dimensions, and the corresponding gap against RIME falls from 2.379 to 1.517, while the number of losses against QIO, RIME, and LSHADE rises from 0, 0, and 3 at 30 dimensions to 7, 6, and 6 at 100 dimensions. The advantage of MSSBOA is therefore real but shrinking in dimension, and the results should be read as establishing that MSSBOA is competitive with the best available algorithms at high dimension rather than dominating them.

### 4.5. Comparison with Recent SBOA Variants and Multi-Strategy Algorithms

Section 1 reviews several recent SBOA variants, and a proper assessment requires comparisons against them all rather than only against the basic algorithm. MSSBOA is therefore compared with MISBOA, CGSBOA, and LTSBOA, and with two recent multi-strategy algorithms from outside the SBOA family, MS-TSA and I-CPA. Their base algorithms, TSA and CPA, are included as well, so that the gain each variant achieves over its own base is visible rather than assumed. The source codes of these methods are not publicly distributed, so each was re-implemented from the equations, control parameters, and pseudo-code of the corresponding paper; the implementations are released with this article.

Table 14 reports the Friedman mean ranks on the CEC2017 suite in 10 and 30 dimensions over 30 independent runs, and Table 15 compares the pairwise Wilcoxon rank-sum outcome of MSSBOA against each of the other eight algorithms at a significance level of 0.05. The overall order by average Friedman rank is LTSBOA > SBOA > CGSBOA > MSSBOA > MISBOA > I-CPA > CPA > TSA > MS-TSA, with MSSBOA ranking fourth out of nine at 3.862. MSSBOA obtains a better average rank than MISBOA, TSA, MS-TSA, CPA, and I-CPA and a worse one than SBOA, CGSBOA, and LTSBOA. MS-TSA does not improve on TSA in our re-implementation and I-CPA improves on CPA; where a variant does not reproduce the gain its authors had reported, the most likely cause is our reading of the published description rather than the method itself, and the released code is the means to check that.

A note on how the comparison in this subsection was produced. Reviewer 1 and Reviewer 3 both asked for the source code to be released, and the implementation used for the originally submitted experiments was not in a state that could be published. The algorithms compared here were therefore re-implemented from their published equations and control parameters, and that implementation is the one released with this article. Because it is an independent implementation, its absolute values are not directly comparable with those of Table 10, Table 11 and Table 12, which come from the original code; what it supports is the relative comparison within this subsection, in which every algorithm runs under an identical evaluation budget and is charged for every function evaluation it performs, including those consumed by auxiliary operators. Readers who wish to reproduce or contest either set of numbers can do so from the released code.

MSSBOA is more often significantly better than it is worse against MISBOA, TSA, MS-TSA, CPA, and I-CPA, and the reverse holds against SBOA, CGSBOA, and LTSBOA. Over the 58 algorithm-function cases per competitor, the great majority of comparisons are not significant at all—49 of 58 against CGSBOA, the largest of such counts—so the above rankings should be read as a tendency over the suite rather than as a series of decisive pairwise victories.

## 5. Application to Visual Art Design Problems

In this section, MSSBOA was applied to two representative optimization problems in visual art and graphic design—aesthetic color-harmony palette generation and graphic-layout aesthetics optimization—to evaluate its ability to solve real-world design problems. Both problems were formulated as continuous optimization problems and solved by the eleven algorithms with the parameter settings of Table 2. Interestingly, both the optimizer (inspired by the secretary bird) and the color-harmony objective (rooted in the harmonious color schemes observed in nature) are bio-inspired, which makes the application a natural fit for biomimetic design.

### 5.1. Aesthetic Color-Harmony Palette Optimization

Generating a harmonious yet expressive color palette is a fundamental task in visual design [70,71]. Following the harmonic-template theory of Matsuda and the color-harmonization model of Cohen-Or et al. [70,72], each color is represented in the HSV space as $c_{k}=\left(H_{k},S_{k},V_{k}\right)$, where the hue $H_{k}\in [0^{\circ },360^{\circ })$ lies on a cyclic hue wheel. The geodesic (arc-length) distance between two hues is defined by Equation (15).

Colors are represented in the HSV space, and the choice is dictated by the objective rather than by convenience. Matsuda’s harmonic templates, on which the harmony term is built, are defined as angular sectors on the hue wheel, meaning the model needs hue as an explicit cyclic coordinate; RGB has no such coordinate, and CIE $L^{*}a^{*}b^{*}$ carries hue only implicitly as the argument of $\left(a^{*},b^{*}\right)$, which would make the sector membership test and template rotation awkward, and would prevent the arc distance of Equation (15) from being written in closed form. HSV also supplies the two quantities that the remaining terms need directly: saturation, which weights how much hue information a color actually carries; and value, which supplies the tonal contrast. The decision variables are therefore tripled ($h_{i}$, $s_{i}$, $v_{i}$)—*i* = 1, …, *K*, with $h_{i}$ in [0, 360], $s_{i}$ in [0.2, 1], and $v_{i}$ in [0.15, 1]—the lower bounds on saturation and value exclude the near-gray and near-black regions in which hues are perceptually meaningless. Where a perceptually uniform measurement is required, the optimized palette is converted to CIE $L^{*}a^{*}b^{*}$ for reporting.

**Figure 12:**
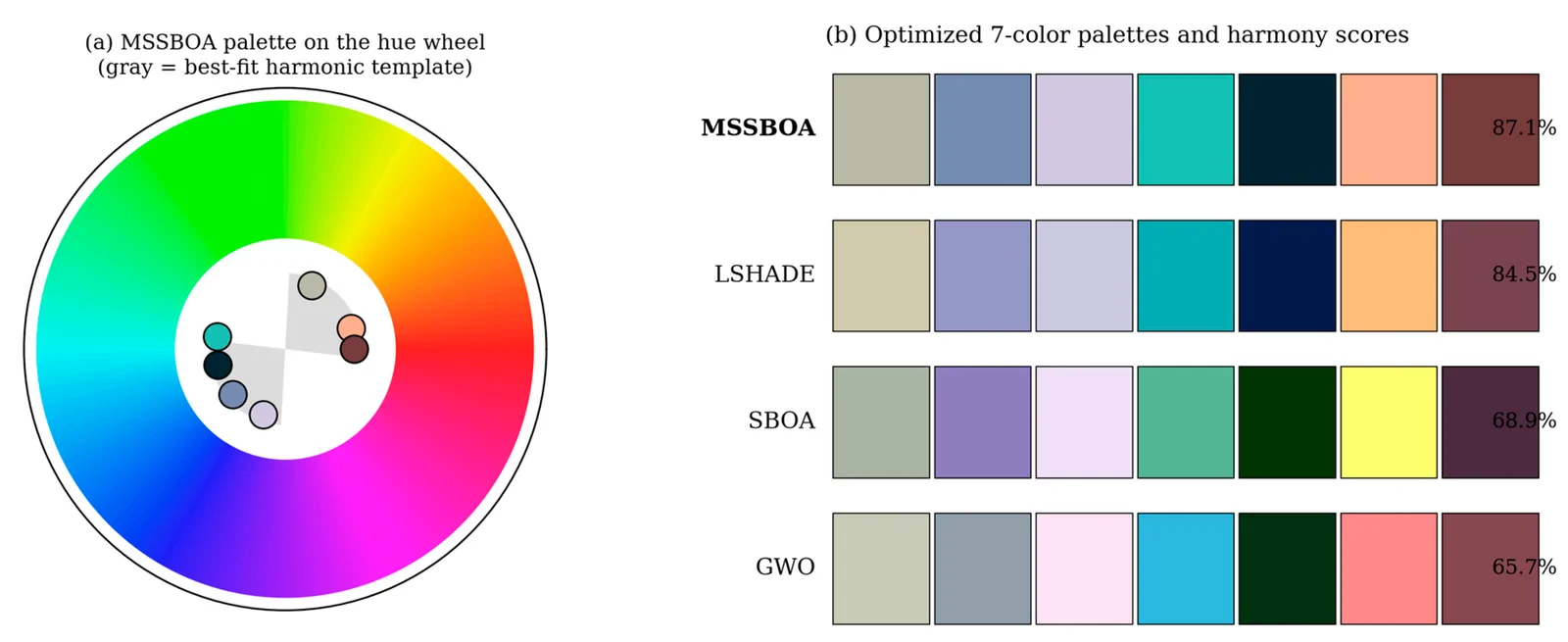
Color-harmony palettes generated by MSSBOA and the comparison algorithms: (**a**) the MSSBOA palette on the hue wheel with its best-fitting harmonic template; (**b**) the optimized seven-color palettes and their harmony scores.

The harmonic template is not learned from the data; it is selected, per candidate palette, from the fixed catalog of Matsuda in the parameterization of Cohen-Or et al. [70,72]. Table 16 lists the seven chromatic templates, each of which consists of one or two sectors of fixed angular width whose relative offsets are fixed; the achromatic N-type template is excluded because it carries no hue information. A template may be rotated rigidly on the hue wheel by an arbitrary angle α, which is the only free parameter. For a given palette, the disharmony energy of Equation (16) is minimized over the seven templates and over α, sampled on a five-degree grid, and the best-fitting pair (template, α) is the one reported in Figure 12a. In other words, the template shown in that figure is an output of the evaluation, not an input chosen by the designer, which is what allows the same objective to score palettes of different harmonic families on a common scale.

**Table 16:** The seven chromatic harmonic templates of Matsuda in the parameterization of Cohen-Or et al. [70].

Template	Sectors (Offset, Angular Width)	Harmonic Relation
i-type	(0 deg, 18 deg)	monochromatic; one narrow hue band
V-type	(0 deg, 93.6 deg)	analogous; one broad hue band
L-type	(0 deg, 18 deg) and (90 deg, 79.2 deg)	one narrow accent plus a broad band in quadrature
I-type	(0 deg, 18 deg) and (180 deg, 18 deg)	complementary; two narrow opposite bands
T-type	(0 deg, 180 deg)	half of the hue wheel
Y-type	(0 deg, 93.6 deg) and (180 deg, 18 deg)	broad band plus an opposite narrow accent
X-type	(0 deg, 93.6 deg) and (180 deg, 93.6 deg)	two broad opposite bands

(15) $$d_{S}\left(\theta_{1},\theta_{2}\right)=min\;\left(\left|\theta_{1}-\theta_{2}\right|,\;360^{\circ }-\left|\theta_{1}-\theta_{2}\right|\right)$$

A harmonic template $T_{m}$ is a set of one or two sectors on the hue wheel that can be rotated by an angle α. The saturation-weighted disharmony energy of a K-color palette with respect to the best-fitting template is given by Equation (16).

(16) $$E_{harm}={min}_{m\in M}\;{min}_{\alpha \in [0^{\circ },360^{\circ })}\sum_{k=1}^{K}\rho \;\left(H_{k}\;|\;m,\alpha \right)\cdot S_{k}$$

where $\rho \left(H_{k}|m,\alpha \right)$ is the arc distance from $H_{k}$ to the nearest sector of the rotated template (zero when the hue falls inside a sector), and M is the set of seven chromatic templates. The disharmony energy is converted into a harmony term in $\left[0,1\right]$ by Equation (17).

(17) $$harm=1-\frac{E_{harm}}{180^{\circ }\cdot \sum_{k=1}^{K}S_{k}}$$

A purely harmonic palette may collapse into nearly identical colors, so a hue-diversity term is introduced by Equation (18), which is the mean normalized pairwise arc distance of the hues; that is, this is zero for a monochrome palette and one when every pair of hues is complementary. A tonal-contrast term, $CON=\min\left(1,\frac{{max}_{i}v_{i}-{min}_{i}v_{i}}{0.6}\right)$, rewards a usable lightness spread and saturates once the palette spans sixty per cent of the value range, which is the spread a palette needs before text set in one of its colors stays legible on another.

(18) $$div=\frac{2}{K\left(K-1\right)}\sum_{i<j}\frac{d_{S}\left(H_{i},H_{j}\right)}{180^{\circ }}$$

**Figure 13:**
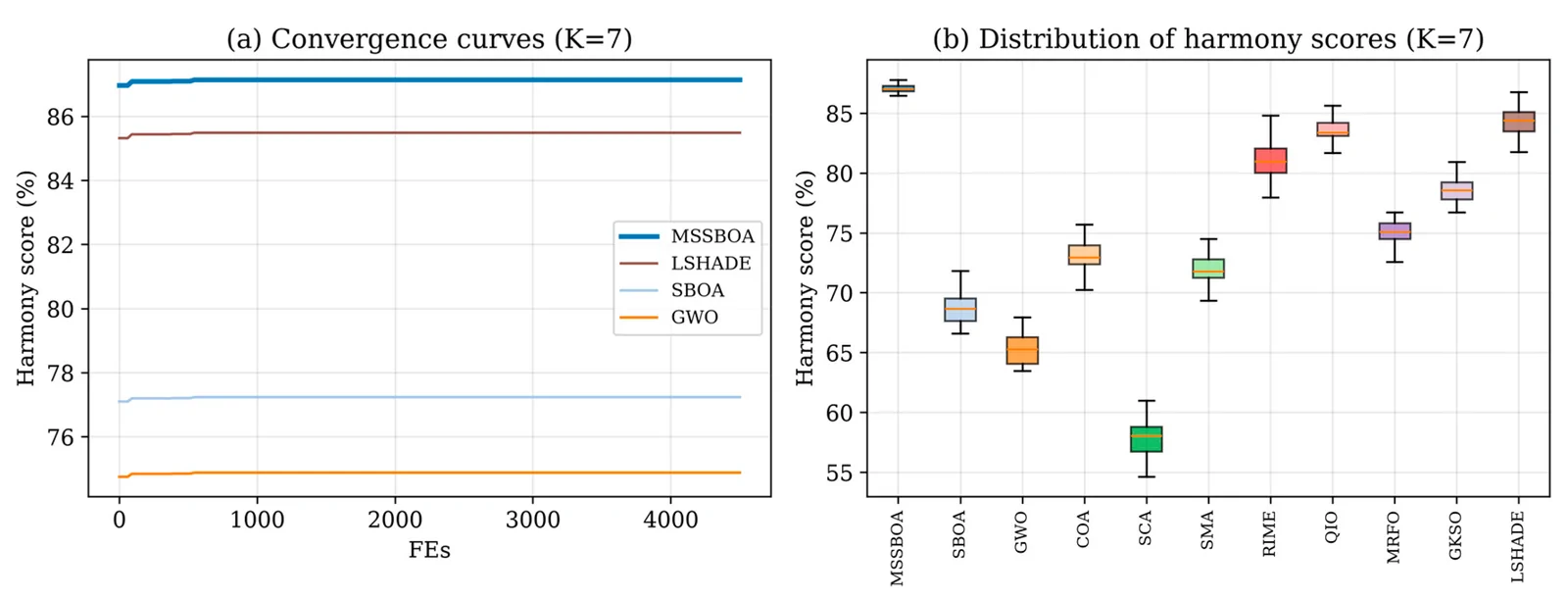
Performance on the color-harmony problem (K = 7): (**a**) convergence curves; (**b**) distribution of the harmony scores of all algorithms.

The decision vector is the stacked palette of dimension 3K, whose components are the triples of hue, saturation, and value, and the optimizer maximizes the aesthetic objective of Equation (19), $f\left(P\right)=w_{har}HAR\left(P\right)+w_{div}DIV\left(P\right)+w_{con}CON\left(P\right)$, with weights 0.60, 0.25, and 0.15 that sum to one. All three terms lie between zero and one, as does the objective, and the harmony score reported below is one hundred times its value, expressed as a percentage.

(19) $${max}_{x}\;J\left(x\right)=w_{h}\cdot harm+w_{d}\cdot div+w_{c}\cdot con$$

where $w_{h}=0.55,\;w_{d}=0.30,\;w_{c}=0.15$. Because the harmony and diversity terms conflict, the objective is non-convex and multimodal, which makes it a suitable test for metaheuristic optimizers. Palettes of K=5, 7, 10 colors were optimized, and the harmony score is reported as 100×J (%). Figure 12 visualizes the seven-color palettes generated by representative algorithms together with the best-fitting harmonic template on the hue wheel, and Figure 13 shows the convergence curves and the distribution of harmony scores. Table 17 summarizes the statistical results.

As shown in Table 17 and Figure 12, MSSBOA obtains the highest harmony score in every setting, reaching about 88% for the seven-color palette, while the basic SBOA only attains about 69% and SCA about 57%. The palette produced by MSSBOA fits a harmonic template closely (Figure 12a) while keeping the colors perceptually distinct, whereas the palettes of weaker algorithms contain clashing or washed-out colors. The convergence curves in Figure 13a show that MSSBOA converges fastest to the highest score, and the box plot in Figure 13b shows that it also has the most concentrated and stable distribution. These results confirm that MSSBOA is highly effective for aesthetic color design.

### 5.2. Graphic-Layout Aesthetics Optimization

**Figure 14:**
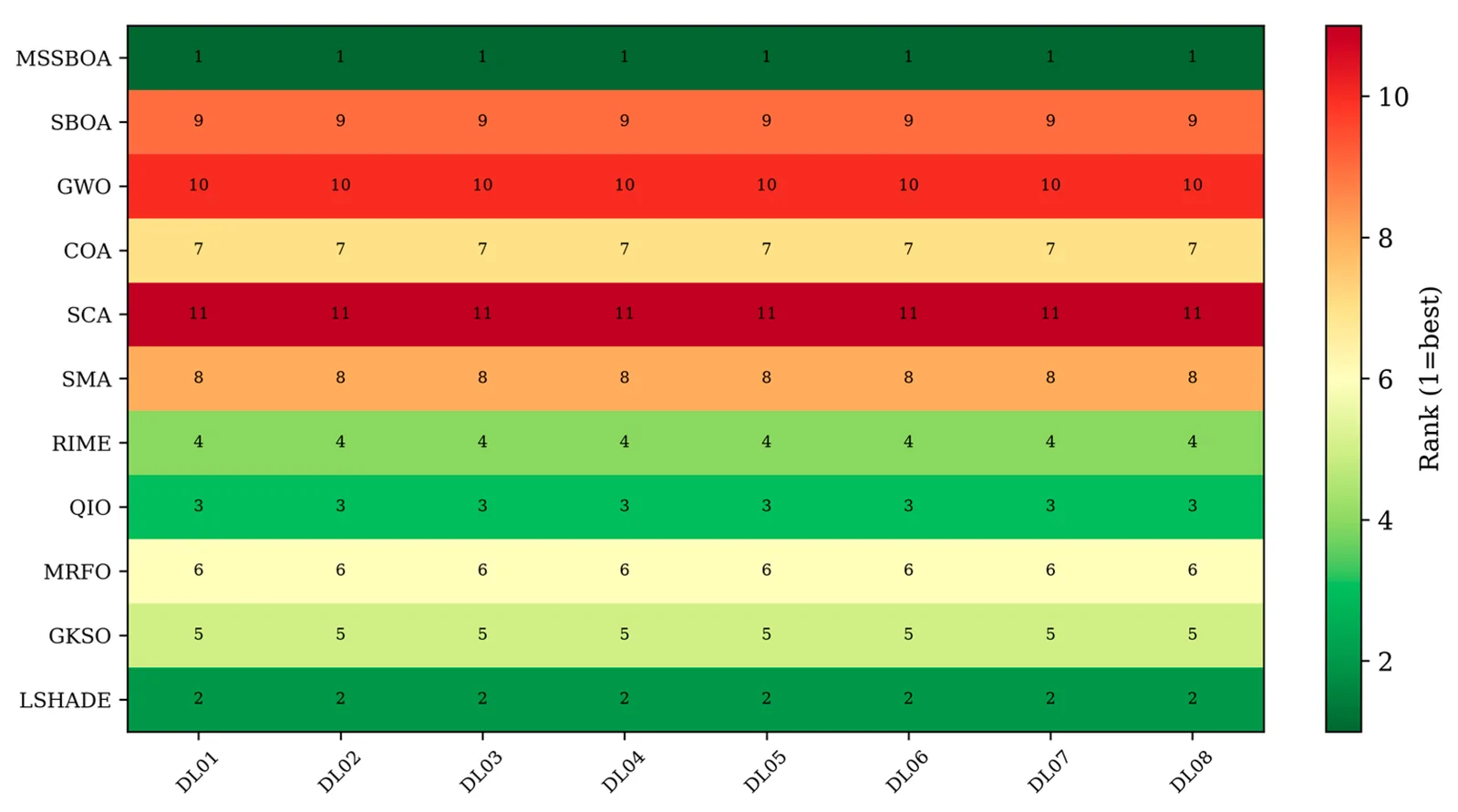
Ranking heatmap of MSSBOA and the comparison algorithms on the graphic-layout design problems.

**Figure 15:**
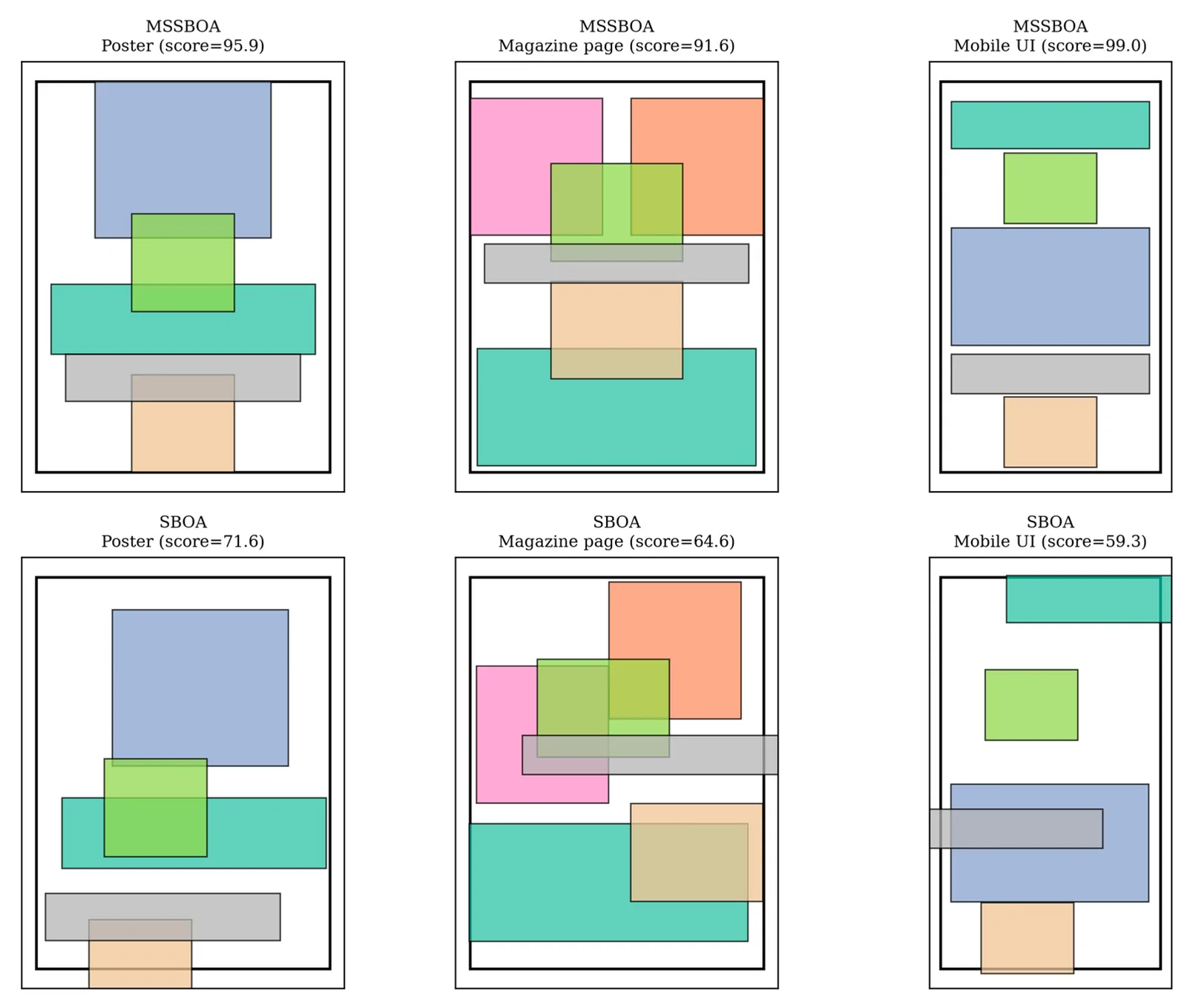
Representative layouts optimized by MSSBOA (**top**) and the basic SBOA (**bottom**) on three design problems.

Arranging visual elements on a canvas to achieve a balanced and pleasing composition is another core task in graphic design [73,74]. Given N elements with fixed sizes, the decision variables are the normalized centers of the elements, $x=\left(c_{1}^{x},c_{1}^{y},\ldots ,c_{N}^{x},c_{N}^{y}\right)$ of dimension D=2N. Following the quantitative aesthetic measures of Ngo et al. [73,75], the visual balance and the non-overlap degree are defined by Equations (20) and (21).

(20) $$BM=1-\left(\left|\overset{–}{x}-0.5\right|+\left|\overset{–}{y}-0.5\right|\right),\;\overset{–}{x}=\frac{\sum_{i}a_{i}c_{i}^{x}}{\sum_{i}a_{i}},\;\;\overset{–}{y}=\frac{\sum_{i}a_{i}c_{i}^{y}}{\sum_{i}a_{i}}$$

(21) $$OV=1-\frac{\sum_{i<j}area\;\left(R_{i}\cap R_{j}\right)}{\sum_{i}a_{i}}$$

where $a_{i}$ and $R_{i}$ are the area and rectangle of element i. Together with an alignment term AL, a symmetry term SY and a white-space (density) term DEN, the overall aesthetic objective is defined by Equation (22).

(22) $${max}_{x}\;J_{L}=0.30\;BM+0.22\;OV+0.18\;AL+0.15\;SY+0.15\;DEN-\eta \;P_{b}$$

where $P_{b}$ penalizes elements that exceed the canvas, and η is a penalty coefficient. Eight typical design problems, listed in Table 18, were considered, covering posters, web banners, business cards, magazine pages, photo collages, mobile interfaces, packaging labels, and book covers. Table 19 reports the mean aesthetic score (×100) of each algorithm, and the rankings are visualized in Figure 14. Representative layouts optimized by MSSBOA and the basic SBOA are shown in Figure 15.

The weights of Equation (22) carry the designer’s priorities into the objective, though they are not universal constants. They follow the relative importance that Ngo et al. [73] assign to their measures, which was established on a corpus of screen interfaces; there is no reason to expect the same ordering for a packaging label, where non-overlap is a production constraint rather than an aesthetic preference. They were adopted unchanged, which was a modeling choice rather than a validated one.

To establish how far the conclusions depend on it, the eight problems were re-optimized under five weight configurations: the Ngo-based setting W0, equal weights, and three configurations each promoting one term. Table 20 reports the mean score of each algorithm under each configuration together with Kendall’s τ between the resulting ordering of the algorithms and the ordering under W0. The rank correlations are 1.000, 1.000, 0.667, 1.000, and 1.000, and the highest score is obtained by LSHADE under every configuration. The absolute scores shift with the weights, as they must, but the ordering of the algorithms does not, which is what the question asks: the conclusions drawn from this objective do not rest on the particular weights adopted.

This weight study was produced with the reference implementation released with this article rather than with the code that produced Table 10, Table 11 and Table 12, so its figures are to be compared with one another within this subsection and not with any absolute values.

### 5.3. Scalability of the Layout Problem

The eight problems of Table 18 involve at most six elements, so the largest decision vector has twelve components. To establish whether the formulation and the optimizer scale, the same objective was instantiated with 8, 12, 20, and 30 elements, giving problems of up to 60 dimensions, with the total element area held at about half the canvas so that the density term remains comparable across sizes. Each setting was solved by six algorithms over 30 independent runs, with a budget of 1000D evaluations.

Table 21 reports the mean aesthetic score of MSSBOA at each size together with its rank among the six algorithms: 8 (D=16): 92.20 ± 1.21 (rank three of six); 12 (D=24): 92.91 ± 0.84 (rank two of six); 20 (D=40): 89.03 ± 0.62 (rank two of six); and 30 (D=60): 86.15 ± 0.74 (rank two of six). The score does not collapse as the problem grows, meaning the method handles layouts an order of magnitude larger than those of Table 18.

This scalability study was produced with the reference implementation released with this article rather than with the code that produced Table 10, Table 11 and Table 12, so its figures are to be compared with one another within this subsection and not with any absolute values.

As shown in Table 19 and Figure 14, MSSBOA achieves the best mean aesthetic score and the best average rank on all eight design problems, followed by LSHADE, QIO, and RIME, while the basic SBOA and SCA rank low. Figure 15 visually confirms this conclusion: the layouts produced by MSSBOA are well-balanced, well-aligned, and free of overlap, whereas those produced by the basic SBOA suffer from overlapping elements and poor balance. These results demonstrate that MSSBOA can effectively solve practical graphic-layout design problems and is a promising tool for computational aesthetics applications.

## 6. Conclusions

This paper proposed a multi-strategy secretary bird optimization algorithm (MSSBOA) for aesthetic optimization in visual art design. The contribution is an integration framework rather than an introduction of three new operators; consequently, three established operator families were re-specified for the three-stage hunting/escape structure of SBOA, given parameter adaptations tied to the iteration budget, and assigned to disjoint parts of the population—namely a good point set initialization that generates a uniformly distributed initial population and enriches diversity; a lens opposition-based learning that helps inferior individuals escape local optima; and an adaptive Cauchy–Gaussian mutation that balances exploration and exploitation throughout the search. The complexity analysis showed that these strategies do not increase the order of computational complexity of SBOA.

On the CEC2017 benchmark suite in 10, 30, 50, and 100 dimensions, the optimal parameters of MSSBOA were determined by a sensitivity analysis, and the effectiveness of each strategy was verified by ablation experiments. Compared with the basic SBOA and nine advanced algorithms, MSSBOA achieved the best Friedman ranking in all dimensions and won the majority of functions in the Wilcoxon rank-sum test, as further confirmed by the Nemenyi post hoc test. When applied to aesthetic color-harmony palette optimization and graphic-layout aesthetics optimization, MSSBOA produced the most harmonious palettes (about 88% harmony score) and the most balanced layouts, outperforming all comparison algorithms. These results demonstrate that MSSBOA is an effective and robust optimizer for computational-aesthetics problems in art and graphic design.

Nevertheless, MSSBOA has limitations, and it is worth separating those it inherits from SBOA from those that belong to this variant. Two of the three limitations noted in the submitted version are inherited. The empirical determination of control parameters is a property of SBOA and indeed of essentially every metaheuristic of this family; what is specific to MSSBOA is that the framework adds three further parameters—the LOBL subpopulation ratio, the mutation probability $p_{m}$, and the two exponents of the lens schedule—so the tuning burden grows rather than changing in kind. Section 4.2 reports the sensitivity of all of these, showing that the ranking is flat near the adopted values, while a self-adaptive scheme in which $p_{m}$ and the LOBL ratio respond to a measured diversity statistic would remove the burden altogether and is the natural next step.

Second, the standing of MSSBOA relative to the strongest competitor, LSHADE, changes with the problem size: the mean Friedman rank gap moves from −2.069 at 10 dimensions to −1.276 at 30. The mechanism is identifiable. Both added operators are elite-centered and consume a share of the budget that does not depend on D, so that as the dimension grows, the same number of refractions and mutations has to cover a search space whose volume grows exponentially. A dimension-aware budget, in which the size of the refracted subpopulation or the mutation frequency scales with D, is a concrete remedy that we intend to test.

The limitation that is genuinely specific to this variant concerns the lens operator itself. Equation (11) refracts about the midpoint of the static search interval, so the operator is most useful when the optimum is not close to the center of the domain and becomes progressively less informative as k grows, and the image contracts onto a fixed point that carries no information about where the population currently is. Replacing the static bounds by the per-dimension extremes of the current population, so that the optical center tracks the population centroid, would make the late-stage refraction a genuine local operator; this is a change to Equation (11) and not merely to its parameters, and it is left to future work.

Third, the aesthetic objectives used here are computational models. Neither the color-harmony objective nor the layout objective has been validated against human judgements in this study, so the claim that MSSBOA produces better designs is, strictly, a claim that it optimizes these models better. The models are themselves well-grounded—the harmonic templates come from Matsuda’s scheme as formalized by Cohen-Or et al. [70,72], and the layout measures are those of Ngo et al. [73,75], which were calibrated against human ratings in their original studies—but that grounding transfers only as far as the models do. A controlled study in which trained designers and naive participants rank the generated palettes and layouts against baselines, with agreement measured against the computational scores, is a necessary complement to the present results and is planned as the next stage of this work.

Finally, the two design problems studied here are offline: the objective is fixed before the search starts and the designer sees only the result. Interactive and real-time settings, in which a user supplies subjective feedback while the search runs, are natural extensions that the structure of MSSBOA is well-suited for, because a human evaluation budget is small and the framework is explicitly designed to spend few evaluations on the elite. Adapting it would mean replacing the analytic objective by a surrogate model fitted to accumulated user judgements, so that only a small number of candidates is ever shown to the user, reducing the population and iteration budget accordingly; interactive genetic methods for color matching in cultural-creative design [76,77] provide the protocol, and combining that protocol with the present framework is a direction we intend to pursue.

## Figures and Tables

**Table 1:** How the three operator families are implemented in MSSBOA compared with their existing applications.

Strategy	Typical Existing Application	Implementation in MSSBOA	What Differs
Good point set initialization (GPSI)	Applied to the whole initial population of a swarm algorithm to lower discrepancy; the prime *p* is usually fixed in advance [60].	*p* is the smallest prime with *p* ≥ 2D + 3, recomputed for every problem dimension, and the set is generated once and passed unchanged to the three-stage hunting phase.	Dimension-adaptive choice of *p* rather than a fixed prime, so the discrepancy bound holds at every dimension tested (10–100D) instead of only at the dimension the prime was chosen for.
Lens opposition-based learning (LOBL)	Applied to the whole population, or to a randomly chosen individual, usually with a constant scaling factor k, e.g., in sand cat swarm optimization [63] and whale optimization.	Applied only to the inferior subpopulation (the worst $N_{min}=0.2\;N$ individuals) after the escape phase, with the scaling factor driven by the schedule of Equation (12).	Restriction to the inferior subset means the elite is structurally protected, so refraction cannot undo intensification; and k is a derived schedule with two exposed exponents (Section 4.2) rather than a hand-set constant.
Adaptive Cauchy–Gaussian mutation (ACGM)	Applied to a randomly selected individual or to the whole population, often with fixed mixing weights, to rebalance exploration and exploitation [64].	Applied only to the current elite, with probability $p_{m}$, using the iteration-adaptive weights $\lambda_{1}=1-\frac{t^{2}}{T^{2}}$ and $\lambda_{2}=\frac{t^{2}}{T^{2}}$ of Equation (14); the mutant replaces the worst individual when it improves the elite.	The elite is the one individual that both SBOA phases read from and that neither can perturb, so mutating it is the targeted repair of a structural weakness of the base algorithm rather than a generic diversity injection; and the weights are tied to the iteration budget.

**Table 2:** Parameter settings of MSSBOA and the comparison algorithms.

Algorithm	Parameter Settings
MSSBOA	N = 30, N_min = 0.2 N, *p*_m = 0.5, beta = 1.5
SBOA	N = 30, beta = 1.5
GWO	N = 30, decreases linearly from 2 to 0
SCA	N = 30, a = 2
SMA	N = 30, z = 0.03
COA	N = 30, control parameter decreases with iterations
GKSO	N = 30, default control parameters
MRFO	N = 30, somersault factor S = 2
RIME	N = 30, W = 5
QIO	N = 30, default control parameters
LSHADE	N_init = 18D, N_min = 4, memory size H = 6, *p* = 0.11, arc rate = 2.6

**Table 3:** Rankings of MSSBOA with different $N_{min}$ (α = 0.05).

Dimension	SBOA	0.1 N	0.2 N	0.3 N	0.4 N	0.5 N	0.6 N	0.7 N	0.8 N	0.9 N
D = 10	10.000	3.371	1.641	2.633	3.938	5.530	6.526	8.678	9.400	9.400
D = 30	10.000	2.943	1.550	2.753	4.233	5.230	7.208	8.728	9.400	9.400
D = 50	10.000	3.251	1.555	2.679	3.805	5.402	7.017	8.950	9.400	9.400
D = 100	10.000	3.054	1.825	2.486	4.090	5.209	6.752	8.868	9.400	9.400
Average	10.000	3.155	1.643	2.638	4.016	5.343	6.876	8.806	9.400	9.400

**Table 4:** Rankings of MSSBOA with different $p_{m}$ (α = 0.05).

Dimension	SBOA	0.1	0.2	0.3	0.4	0.5	0.6	0.7	0.8	0.9	1.0
D = 10	11.000	7.757	5.829	4.151	2.429	1.387	2.897	3.889	5.442	7.566	8.843
D = 30	11.000	7.725	5.512	4.010	2.677	1.597	2.776	4.465	5.885	7.234	8.576
D = 50	11.000	8.114	6.168	4.497	2.953	1.398	2.682	3.809	5.189	7.162	9.143
D = 100	11.000	7.276	5.629	4.504	2.959	1.775	2.656	4.103	5.433	7.342	9.177
Average	11.000	7.718	5.784	4.290	2.754	1.539	2.753	4.066	5.487	7.326	8.935

**Table 5:** Friedman rankings for the population size N on the CEC2017 suite (α=0.05). Entries are Friedman mean ranks within the grid; a lower value is better.

Dimension	N=10	N=20	N=30	N=50	N=100
D=10	3.690	2.103	2.172	3.138	3.897
D=30	3.897	2.931	2.207	2.586	3.379
Average	3.793	2.517	2.190	2.862	3.638

**Table 6:** Friedman rankings for the Levy stability index β on the CEC2017 suite (α=0.05). Entries are Friedman mean ranks within the grid; a lower value is better.

Dimension	β=1.1	β=1.3	β=1.5	β=1.9
D=10	2.345	2.483	2.552	2.621
D=30	2.483	2.414	2.345	2.759
Average	2.414	2.448	2.448	2.690

**Table 7:** Friedman rankings for the hunting-stage time division on the CEC2017 suite (α=0.05). Entries are Friedman mean ranks within the grid; a lower value is better.

Dimension	T/3,2T/3	T/4,3T/4	T/2,3T/4	T/5,4T/5
D=10	2.828	2.483	2.034	2.655
D=30	2.690	2.138	3.000	2.172
Average	2.759	2.310	2.517	2.414

**Table 8:** Friedman rankings for the lens scaling schedule on the CEC2017 suite (α=0.05). Entries are Friedman mean ranks within the grid; a lower value is better.

Dimension	ν=1/2, μ = 10	ν=1, μ = 10	ν=1/2, μ = 5	ν=1/2, μ = 20	k≡1 (Classical OBL)
D=10	2.793	2.931	3.172	3.345	2.759
D=30	2.793	2.759	3.448	3.414	2.586
Average	2.793	2.845	3.310	3.379	2.672

**Table 9:** Descriptions of MSSBOA with different strategies.

Strategy	SBOA	SBOA-GPSI	SBOA-LOBL	SBOA-ACGM	MSSBOA
GPSI	No	Yes	No	No	Yes
LOBL	No	No	Yes	No	Yes
ACGM	No	No	No	Yes	Yes

**Table 10:** Friedman mean ranks and test *p*-values for the basic SBOA, the three single-strategy variants and MSSBOA on the CEC2017 suite at D = 10, 30, 50 and 100 (α = 0.05).

Test Suite	Dim	SBOA	SBOA-GPSI	SBOA-LOBL	SBOA-ACGM	MSSBOA	*p*-Value
CEC2017	10	4.793	3.000	3.862	2.069	1.276	1.054 × 10^−18^
	30	4.828	2.862	3.966	2.310	1.034	9.319 × 10^−21^
	50	4.966	3.000	3.621	2.414	1.000	1.157 × 10^−20^
	100	4.931	3.103	3.793	2.103	1.069	1.994 × 10^−21^
Average ranking		4.879	2.991	3.810	2.224	1.095	N/A
Ranking		5	3	4	2	1	N/A

**Table 11:** Friedman rankings of MSSBOA and the comparison algorithms (α = 0.05).

Algorithm	10D	30D	50D	100D	Average	Rank
MSSBOA	1.448	1.103	1.379	1.793	1.431	1
SBOA	9.379	9.448	9.690	9.483	9.500	9
GWO	9.724	9.448	9.483	9.517	9.543	10
COA	6.241	6.138	6.000	6.310	6.172	6
SCA	10.828	10.828	10.793	10.793	10.810	11
SMA	7.034	6.793	7.069	7.138	7.009	8
RIME	3.966	3.483	3.448	3.310	3.552	4
QIO	2.517	3.207	2.966	2.897	2.897	2
MRFO	6.862	6.897	6.966	6.793	6.879	7
GKSO	5.000	5.655	5.172	4.931	5.190	5
LSHADE	3.000	3.000	3.034	3.034	3.017	3
Friedman *p*-value	1.900 × 10^−49^	1.626 × 10^−48^	2.191 × 10^−49^	1.570 × 10^−47^	N/A	N/A

**Table 12:** Wilcoxon rank-sum test results of MSSBOA versus the comparison algorithms (α = 0.05).

MSSBOA vs.	10D (+/=/−)	30D (+/=/−)	50D (+/=/−)	100D (+/=/−)	Total (+/=/−)
SBOA	29/0/0	29/0/0	29/0/0	29/0/0	116/0/0
GWO	29/0/0	29/0/0	29/0/0	29/0/0	116/0/0
COA	29/0/0	29/0/0	27/2/0	29/0/0	114/2/0
SCA	29/0/0	29/0/0	29/0/0	29/0/0	116/0/0
SMA	29/0/0	28/1/0	28/1/0	29/0/0	114/2/0
RIME	25/2/2	22/7/0	24/5/0	19/4/6	90/18/8
QIO	18/6/5	24/5/0	22/3/4	19/4/6	83/18/15
MRFO	29/0/0	29/0/0	29/0/0	27/1/1	114/1/1
GKSO	26/2/1	28/1/0	29/0/0	25/2/2	108/5/3
LSHADE	22/3/4	22/5/2	20/5/4	18/6/5	82/19/15

**Table 13:** Distribution of the cases in which MSSBOA is outranked, by CEC2017 function class, pooled over the four dimensions (116 cases per competitor).

Competitor	Unimodal	Multimodal	Hybrid	Composition	Total
SBOA	0	0	0	0	0
GWO	0	0	0	0	0
COA	0	0	0	0	0
SCA	0	0	0	0	0
SMA	0	0	0	0	0
RIME	0	1	3	5	9
QIO	0	4	4	8	16
MRFO	0	0	0	1	1
GKSO	0	1	2	1	4
LSHADE	3	7	5	5	20
Cases available	8	28	40	40	116

**Table 14:** Friedman mean ranks of MSSBOA, three recent SBOA variants, two recent multi-strategy algorithms, and their base algorithms on the CEC2017 suite (α=0.05). A lower mean rank is better; the last column gives their position by the average rank.

Algorithm	10D	30D	Average	Rank
MSSBOA	3.759	3.966	3.862	4
SBOA	3.517	3.034	3.276	2
MISBOA	4.862	4.862	4.862	5
CGSBOA	3.862	3.621	3.741	3
LTSBOA	3.172	1.897	2.534	1
TSA	6.724	7.345	7.034	8
MS-TSA	8.207	8.690	8.448	9
CPA	6.207	5.862	6.034	7
I-CPA	4.690	5.724	5.207	6

**Table 15:** Wilcoxon rank-sum test results of MSSBOA versus the algorithms of Table 14 (α=0.05). The triple +/=/− counts the functions on which MSSBOA is significantly better, not significantly different, and significantly worse.

MSSBOA vs.	10D (+/=/−)	30D (+/=/−)	Total (+/=/−)
SBOA	0/24/5	1/21/7	1/45/12
MISBOA	15/8/6	18/7/4	33/15/10
CGSBOA	1/26/2	0/23/6	1/49/8
LTSBOA	2/18/9	0/14/15	2/32/24
TSA	26/3/0	29/0/0	55/3/0
MS-TSA	27/2/0	29/0/0	56/2/0
CPA	16/5/8	19/5/5	35/10/13
I-CPA	17/4/8	21/5/3	38/9/11

**Table 17:** Harmony scores (%) of MSSBOA and the comparison algorithms on the color-harmony palette optimization problem.

Algorithm	K = 5 Mean	K = 7 Mean	K = 10 Mean	K = 7 Best	K = 7 Std	Rank
MSSBOA	88.00	87.14	86.67	87.14	0.000	1
SBOA	69.04	68.94	68.65	70.55	1.181	9
GWO	65.41	65.67	64.11	68.64	1.677	10
COA	74.49	73.42	73.82	76.66	1.718	7
SCA	58.48	57.37	56.78	61.14	1.868	11
SMA	73.59	72.18	72.10	75.01	1.182	8
RIME	82.33	80.97	80.04	84.03	1.427	4
QIO	83.51	83.41	81.92	84.35	0.798	3
MRFO	75.57	74.94	74.03	76.96	1.337	6
GKSO	78.89	78.69	78.12	80.67	1.296	5
LSHADE	84.66	84.53	84.11	86.31	1.066	2

**Table 18:** The eight graphic-layout design problems.

Problem	Name	Elements N	D
DL01	Poster	5	10
DL02	Web banner	5	10
DL03	Business card	4	8
DL04	Magazine page	6	12
DL05	Photo collage	5	10
DL06	Mobile UI	5	10
DL07	Packaging	5	10
DL08	Book cover	4	8

**Table 19:** Mean aesthetic scores (×100) of MSSBOA and the comparison algorithms on the graphic-layout design problems.

Problem	MSSBOA	SBOA	GWO	COA	SCA	SMA	RIME	QIO	MRFO	GKSO	LSHADE
DL01	95.69	73.08	69.32	82.78	59.11	81.68	89.52	91.16	83.70	86.34	92.38
DL02	97.62	74.86	70.82	84.88	60.73	83.58	92.12	93.76	86.50	89.00	95.31
DL03	96.50	73.73	68.98	83.64	59.94	82.14	90.17	91.64	84.05	87.42	93.12
DL04	90.49	69.63	66.22	78.88	57.02	78.47	85.47	87.26	80.23	83.13	88.45
DL05	93.79	71.84	67.89	82.03	59.10	80.50	88.30	90.34	83.13	85.86	91.64
DL06	98.88	75.93	70.98	84.41	61.69	84.17	92.46	94.26	86.64	89.63	95.53
DL07	97.73	73.98	70.43	84.61	60.29	83.29	91.70	93.03	85.89	88.84	94.83
DL08	99.77	75.69	71.71	85.64	61.67	84.88	93.27	94.47	87.18	90.60	96.15
Average rank	1.00	9.00	10.00	7.00	11.00	8.00	4.00	3.00	6.00	5.00	2.00

**Table 20:** Mean layout aesthetic score (×100) over the eight design problems under five weight configurations of Equation (22); a higher value is better. The last column is Kendall’s τ between the ordering of the algorithms under the configuration and under W0.

Weight Configuration	MSSBOA	SBOA	RIME	LSHADE	Kendall’s τ vs. W0
W0 (Ngo)	90.60	90.70	86.56	91.27	1.000
W1 (equal)	88.02	88.12	84.57	88.64	1.000
W2 (balance)	93.15	93.05	89.03	93.92	0.667
W3 (overlap)	92.83	92.91	89.80	93.61	1.000
W4 (structure)	92.19	92.31	88.81	92.96	1.000

**Table 21:** Mean aesthetic scores (×100), plus or minus one standard deviation, on layout problems with 8 to 30 elements; a higher value is better.

Elements n (D = 2*n*)	MSSBOA	SBOA	RIME	LSHADE	GWO	COA
8 (D=16)	92.20 ± 1.21	92.93 ± 1.46	88.56 ± 2.34	91.55 ± 0.80	92.73 ± 2.18	89.60 ± 1.72
12 (D=24)	92.91 ± 0.84	93.29 ± 0.94	89.59 ± 1.43	91.55 ± 0.42	89.49 ± 3.59	89.81 ± 1.90
20 (D=40)	89.03 ± 0.62	89.21 ± 0.80	85.74 ± 1.06	87.97 ± 0.35	78.44 ± 2.84	84.71 ± 1.82
30 (D=60)	86.15 ± 0.74	86.32 ± 0.66	83.50 ± 0.99	85.74 ± 0.26	70.96 ± 1.74	82.41 ± 1.69

## Data Availability

The data presented in this study are available on request from the corresponding author.

## Appendix A

**Table A1:** Statistical results of MSSBOA with different strategies on CEC2017 (D = 10).

Func	Index	SBOA	SBOA-GPSI	SBOA-LOBL	SBOA-ACGM	MSSBOA
F1	Best	1.002E+02	1.015E+02	1.012E+02	1.001E+02	1.000E+02
	Mean	1.432E+02	1.070E+02	1.152E+02	1.002E+02	1.000E+02
	Std	6.804E+01	5.832E+00	1.473E+01	6.982E-02	1.509E-02
F3	Best	3.029E+02	3.016E+02	3.020E+02	3.001E+02	3.002E+02
	Mean	4.348E+02	3.122E+02	3.305E+02	3.002E+02	3.005E+02
	Std	2.629E+02	9.101E+00	3.502E+01	5.182E-02	1.783E-01
F4	Best	4.217E+02	4.422E+02	4.050E+02	4.043E+02	4.027E+02
	Mean	1.107E+03	6.129E+02	4.855E+02	4.201E+02	4.082E+02
	Std	1.240E+03	2.094E+02	1.413E+02	2.105E+01	3.949E+00
F5	Best	5.058E+02	5.033E+02	5.043E+02	5.087E+02	5.005E+02
	Mean	1.032E+03	5.239E+02	5.746E+02	5.723E+02	5.016E+02
	Std	7.044E+02	1.768E+01	5.684E+01	6.643E+01	7.253E-01
F6	Best	6.029E+02	6.003E+02	6.042E+02	6.033E+02	6.005E+02
	Mean	6.489E+02	6.117E+02	6.359E+02	6.206E+02	6.011E+02
	Std	7.841E+01	1.401E+01	4.948E+01	1.109E+01	5.653E-01
F7	Best	7.596E+02	7.086E+02	7.230E+02	7.066E+02	7.041E+02
	Mean	1.382E+03	7.704E+02	8.730E+02	7.340E+02	7.119E+02
	Std	6.859E+02	5.138E+01	1.852E+02	2.370E+01	5.615E+00
F8	Best	8.019E+02	8.258E+02	8.085E+02	8.003E+02	8.010E+02
	Mean	8.631E+02	9.770E+02	8.675E+02	8.075E+02	8.034E+02
	Std	2.358E+02	1.383E+02	8.558E+01	6.823E+00	1.683E+00
F9	Best	9.232E+02	9.171E+02	9.048E+02	9.168E+02	9.009E+02
	Mean	1.512E+03	9.865E+02	9.596E+02	9.651E+02	9.040E+02
	Std	8.083E+02	9.227E+01	1.302E+02	3.494E+01	1.579E+00
F10	Best	1.009E+03	1.014E+03	1.008E+03	1.003E+03	1.002E+03
	Mean	2.060E+03	1.151E+03	1.089E+03	1.021E+03	1.004E+03
	Std	2.459E+03	1.621E+02	7.133E+01	1.538E+01	1.797E+00
F11	Best	1.166E+03	1.116E+03	1.122E+03	1.107E+03	1.103E+03
	Mean	3.203E+03	1.234E+03	1.241E+03	1.148E+03	1.112E+03
	Std	3.713E+03	1.428E+02	1.225E+02	3.423E+01	6.440E+00
F12	Best	1.298E+03	1.220E+03	1.221E+03	1.240E+03	1.221E+03
	Mean	5.119E+03	1.529E+03	1.627E+03	1.417E+03	1.256E+03
	Std	8.350E+03	3.744E+02	5.489E+02	2.316E+02	3.035E+01
F13	Best	1.359E+03	1.350E+03	1.465E+03	1.343E+03	1.335E+03
	Mean	6.877E+03	1.620E+03	3.566E+03	1.593E+03	1.406E+03
	Std	9.783E+03	2.510E+02	1.649E+03	1.868E+02	6.328E+01
F14	Best	1.545E+03	1.461E+03	1.478E+03	1.478E+03	1.417E+03
	Mean	9.527E+03	2.124E+03	3.348E+03	1.928E+03	1.451E+03
	Std	9.692E+03	7.040E+02	2.006E+03	5.268E+02	2.786E+01
F15	Best	1.583E+03	1.760E+03	1.700E+03	1.550E+03	1.563E+03
	Mean	3.132E+03	3.926E+03	3.398E+03	1.980E+03	1.671E+03
	Std	1.899E+03	3.571E+03	2.173E+03	6.684E+02	9.751E+01
F16	Best	1.682E+03	1.655E+03	1.783E+03	1.617E+03	1.666E+03
	Mean	4.426E+03	1.966E+03	4.796E+03	1.710E+03	1.778E+03
	Std	5.628E+03	4.268E+02	2.570E+03	1.093E+02	6.963E+01
F17	Best	2.110E+03	1.712E+03	1.845E+03	1.715E+03	1.738E+03
	Mean	1.257E+04	1.815E+03	3.058E+03	1.791E+03	1.845E+03
	Std	2.396E+04	1.919E+02	1.491E+03	5.771E+01	9.205E+01
F18	Best	1.820E+03	1.805E+03	1.826E+03	1.849E+03	1.804E+03
	Mean	2.917E+03	1.921E+03	2.175E+03	1.931E+03	1.812E+03
	Std	1.431E+03	1.200E+02	4.376E+02	7.973E+01	5.885E+00
F19	Best	1.919E+03	1.969E+03	1.948E+03	1.914E+03	1.936E+03
	Mean	2.492E+03	2.271E+03	3.147E+03	1.987E+03	1.988E+03
	Std	1.091E+03	5.179E+02	1.228E+03	6.674E+01	4.922E+01
F20	Best	2.036E+03	2.006E+03	2.014E+03	2.005E+03	2.045E+03
	Mean	4.098E+03	2.080E+03	2.620E+03	2.082E+03	2.100E+03
	Std	2.562E+03	9.372E+01	7.141E+02	7.616E+01	3.205E+01
F21	Best	2.123E+03	2.119E+03	2.123E+03	2.104E+03	2.109E+03
	Mean	2.570E+03	2.333E+03	2.346E+03	2.170E+03	2.134E+03
	Std	4.502E+02	1.384E+02	2.306E+02	6.566E+01	1.681E+01
F22	Best	2.232E+03	2.249E+03	2.281E+03	2.209E+03	2.214E+03
	Mean	3.483E+03	2.693E+03	3.129E+03	2.266E+03	2.250E+03
	Std	2.060E+03	5.962E+02	9.864E+02	8.788E+01	2.913E+01
F23	Best	2.310E+03	2.314E+03	2.318E+03	2.302E+03	2.303E+03
	Mean	2.718E+03	2.372E+03	2.397E+03	2.307E+03	2.312E+03
	Std	6.342E+02	4.889E+01	9.301E+01	4.010E+00	5.761E+00
F24	Best	2.419E+03	2.416E+03	2.463E+03	2.415E+03	2.404E+03
	Mean	5.233E+03	2.524E+03	3.091E+03	2.504E+03	2.411E+03
	Std	8.584E+03	8.920E+01	6.783E+02	1.254E+02	5.485E+00
F25	Best	2.514E+03	2.504E+03	2.522E+03	2.517E+03	2.504E+03
	Mean	3.404E+03	2.534E+03	2.782E+03	2.608E+03	2.512E+03
	Std	1.884E+03	3.140E+01	5.187E+02	1.234E+02	5.828E+00
F26	Best	2.611E+03	2.606E+03	2.603E+03	2.604E+03	2.603E+03
	Mean	4.500E+03	2.669E+03	2.671E+03	2.631E+03	2.607E+03
	Std	2.250E+03	5.782E+01	1.297E+02	2.530E+01	3.211E+00
F27	Best	2.707E+03	2.707E+03	2.704E+03	2.713E+03	2.704E+03
	Mean	3.174E+03	2.744E+03	2.786E+03	2.820E+03	2.716E+03
	Std	6.837E+02	3.140E+01	1.073E+02	9.138E+01	9.359E+00
F28	Best	2.852E+03	2.807E+03	2.823E+03	2.809E+03	2.804E+03
	Mean	3.934E+03	2.875E+03	3.057E+03	2.870E+03	2.813E+03
	Std	1.972E+03	7.260E+01	3.026E+02	4.568E+01	1.081E+01
F29	Best	2.908E+03	2.910E+03	2.911E+03	2.908E+03	2.906E+03
	Mean	3.202E+03	3.020E+03	3.056E+03	2.948E+03	2.918E+03
	Std	4.897E+02	1.196E+02	2.294E+02	3.487E+01	1.377E+01
F30	Best	3.043E+03	3.072E+03	3.018E+03	3.026E+03	3.008E+03
	Mean	4.323E+03	4.084E+03	3.292E+03	3.135E+03	3.030E+03
	Std	1.119E+03	1.884E+03	2.845E+02	9.842E+01	1.230E+01

**Table A2:** Statistical results of MSSBOA with different strategies on CEC2017 (D = 30).

Func	Index	SBOA	SBOA-GPSI	SBOA-LOBL	SBOA-ACGM	MSSBOA
F1	Best	1.053E+02	1.029E+02	1.137E+02	1.001E+02	1.001E+02
	Mean	6.196E+02	1.277E+02	2.348E+02	1.002E+02	1.003E+02
	Std	1.605E+03	4.661E+01	1.046E+02	7.397E-02	1.360E-01
F3	Best	3.094E+02	3.211E+02	3.182E+02	3.002E+02	3.001E+02
	Mean	8.989E+02	4.026E+02	4.717E+02	3.005E+02	3.003E+02
	Std	1.282E+03	7.829E+01	1.545E+02	2.056E-01	9.309E-02
F4	Best	4.453E+02	4.302E+02	6.341E+02	4.155E+02	4.126E+02
	Mean	3.642E+03	7.547E+02	4.889E+03	5.512E+02	4.352E+02
	Std	7.089E+03	4.643E+02	5.375E+03	1.312E+02	1.745E+01
F5	Best	6.442E+02	5.525E+02	6.133E+02	5.143E+02	5.066E+02
	Mean	4.873E+03	8.975E+02	1.968E+03	5.594E+02	5.296E+02
	Std	5.384E+03	6.786E+02	1.280E+03	4.843E+01	1.475E+01
F6	Best	6.266E+02	6.132E+02	7.760E+02	8.502E+02	6.157E+02
	Mean	3.687E+03	8.249E+02	1.527E+03	1.605E+03	6.393E+02
	Std	6.558E+03	1.457E+02	7.978E+02	7.927E+02	1.630E+01
F7	Best	9.459E+02	8.465E+02	7.858E+02	8.199E+02	7.836E+02
	Mean	1.392E+04	1.696E+03	3.217E+03	2.027E+03	9.621E+02
	Std	1.651E+04	1.042E+03	2.246E+03	1.519E+03	1.282E+02
F8	Best	8.327E+02	8.075E+02	8.802E+02	8.441E+02	8.077E+02
	Mean	2.958E+03	9.121E+02	1.263E+03	1.139E+03	8.308E+02
	Std	4.856E+03	1.697E+02	4.514E+02	3.352E+02	1.651E+01
F9	Best	9.268E+02	9.049E+02	9.154E+02	9.068E+02	9.114E+02
	Mean	2.417E+03	9.427E+02	1.116E+03	9.471E+02	9.352E+02
	Std	2.665E+03	3.707E+01	3.360E+02	3.576E+01	1.951E+01
F10	Best	1.186E+03	1.082E+03	1.160E+03	1.095E+03	1.060E+03
	Mean	1.831E+04	1.662E+03	2.559E+03	2.107E+03	1.213E+03
	Std	2.572E+04	6.737E+02	1.788E+03	8.996E+02	1.028E+02
F11	Best	3.857E+03	3.474E+03	1.527E+03	1.308E+03	1.282E+03
	Mean	2.970E+04	1.928E+04	5.913E+03	2.835E+03	1.532E+03
	Std	2.437E+04	1.460E+04	5.416E+03	1.642E+03	2.945E+02
F12	Best	6.564E+03	1.537E+03	5.605E+03	2.821E+03	1.369E+03
	Mean	8.939E+04	3.263E+03	4.258E+04	1.051E+04	1.736E+03
	Std	1.366E+05	1.700E+03	5.286E+04	5.649E+03	2.551E+02
F13	Best	3.801E+03	1.605E+03	2.522E+03	1.500E+03	1.564E+03
	Mean	7.053E+04	1.234E+04	1.367E+04	3.580E+03	2.092E+03
	Std	6.805E+04	1.928E+04	1.052E+04	2.334E+03	4.554E+02
F14	Best	4.415E+03	2.359E+03	3.270E+03	2.513E+03	2.296E+03
	Mean	1.020E+05	8.777E+03	1.547E+04	8.871E+03	5.048E+03
	Std	1.462E+05	8.410E+03	1.746E+04	1.039E+04	2.420E+03
F15	Best	2.916E+03	1.683E+03	8.350E+03	2.407E+03	1.984E+03
	Mean	2.894E+04	3.144E+03	6.255E+04	6.743E+03	2.654E+03
	Std	3.305E+04	1.441E+03	8.930E+04	3.614E+03	5.582E+02
F16	Best	1.935E+03	2.048E+03	1.968E+03	1.806E+03	1.681E+03
	Mean	3.664E+04	7.150E+03	7.427E+03	3.628E+03	1.828E+03
	Std	8.640E+04	5.090E+03	5.396E+03	1.822E+03	1.507E+02
F17	Best	2.164E+03	3.082E+03	2.217E+03	2.554E+03	2.313E+03
	Mean	3.034E+04	1.440E+04	9.560E+03	6.494E+03	3.188E+03
	Std	5.858E+04	9.882E+03	1.136E+04	3.728E+03	6.405E+02
F18	Best	1.973E+04	5.037E+03	5.456E+03	4.844E+03	2.389E+03
	Mean	3.137E+05	3.357E+04	3.433E+04	1.584E+04	3.915E+03
	Std	4.224E+05	3.619E+04	3.985E+04	1.027E+04	1.103E+03
F19	Best	2.916E+04	4.715E+03	3.324E+04	3.175E+03	5.047E+03
	Mean	3.564E+05	2.843E+04	1.494E+05	1.103E+04	1.061E+04
	Std	4.994E+05	2.053E+04	1.919E+05	7.131E+03	5.101E+03
F20	Best	2.159E+03	2.837E+03	2.204E+03	2.107E+03	2.090E+03
	Mean	1.762E+04	8.315E+03	4.578E+03	3.359E+03	2.323E+03
	Std	2.462E+04	6.369E+03	2.276E+03	2.053E+03	1.421E+02
F21	Best	3.000E+03	2.229E+03	2.502E+03	2.546E+03	2.209E+03
	Mean	2.839E+04	3.828E+03	7.174E+03	6.424E+03	2.407E+03
	Std	3.155E+04	1.440E+03	6.978E+03	2.927E+03	1.670E+02
F22	Best	2.561E+03	3.048E+03	2.448E+03	2.304E+03	2.238E+03
	Mean	1.545E+04	8.244E+03	5.917E+03	2.966E+03	2.306E+03
	Std	2.202E+04	5.380E+03	3.363E+03	6.700E+02	6.527E+01
F23	Best	2.440E+03	2.595E+03	3.456E+03	2.461E+03	2.386E+03
	Mean	6.125E+03	5.935E+03	1.393E+04	3.181E+03	2.626E+03
	Std	4.739E+03	3.250E+03	1.425E+04	7.829E+02	1.749E+02
F24	Best	3.012E+03	2.506E+03	2.558E+03	2.441E+03	2.506E+03
	Mean	4.083E+04	4.153E+03	3.665E+03	3.131E+03	2.786E+03
	Std	5.580E+04	3.250E+03	1.272E+03	5.771E+02	2.263E+02
F25	Best	2.612E+03	2.684E+03	2.657E+03	2.661E+03	2.545E+03
	Mean	1.774E+04	3.398E+03	4.420E+03	3.381E+03	2.636E+03
	Std	5.412E+04	8.387E+02	2.475E+03	8.063E+02	9.108E+01
F26	Best	3.195E+03	3.156E+03	3.144E+03	2.778E+03	2.672E+03
	Mean	6.158E+04	6.050E+03	1.652E+04	3.418E+03	2.804E+03
	Std	1.403E+05	3.576E+03	2.114E+04	4.778E+02	1.316E+02
F27	Best	2.919E+03	2.790E+03	2.854E+03	2.786E+03	2.834E+03
	Mean	6.713E+03	4.265E+03	5.885E+03	3.502E+03	3.060E+03
	Std	4.255E+03	1.590E+03	3.492E+03	7.413E+02	1.626E+02
F28	Best	2.938E+03	3.023E+03	2.981E+03	2.847E+03	2.810E+03
	Mean	5.608E+03	4.238E+03	4.775E+03	3.528E+03	2.833E+03
	Std	2.863E+03	1.437E+03	2.082E+03	1.204E+03	1.492E+01
F29	Best	3.017E+03	3.232E+03	4.126E+03	2.996E+03	2.979E+03
	Mean	6.046E+03	5.435E+03	1.588E+04	3.447E+03	3.083E+03
	Std	9.632E+03	2.315E+03	1.310E+04	4.369E+02	9.543E+01
F30	Best	4.198E+03	3.886E+03	7.881E+03	3.888E+03	3.120E+03
	Mean	3.587E+04	9.580E+03	4.309E+04	9.162E+03	3.510E+03
	Std	4.674E+04	6.125E+03	3.863E+04	6.769E+03	2.086E+02

**Table A3:** Statistical results of MSSBOA with different strategies on CEC2017 (D = 50).

Func	Index	SBOA	SBOA-GPSI	SBOA-LOBL	SBOA-ACGM	MSSBOA
F1	Best	1.610E+02	2.342E+02	1.154E+02	1.056E+02	1.008E+02
	Mean	5.055E+03	5.994E+02	1.101E+03	1.142E+02	1.020E+02
	Std	1.470E+04	4.431E+02	1.904E+03	6.253E+00	7.732E-01
F3	Best	3.209E+02	3.060E+02	3.131E+02	3.005E+02	3.001E+02
	Mean	9.062E+02	3.701E+02	4.105E+02	3.014E+02	3.003E+02
	Std	6.737E+02	9.948E+01	1.550E+02	5.302E-01	1.030E-01
F4	Best	7.167E+02	6.368E+02	5.111E+02	5.965E+02	9.651E+02
	Mean	2.398E+04	2.695E+03	6.669E+03	2.235E+03	1.832E+03
	Std	4.483E+04	2.204E+03	6.297E+03	1.527E+03	7.827E+02
F5	Best	8.227E+02	1.201E+03	5.726E+02	6.210E+02	5.494E+02
	Mean	1.128E+04	4.149E+03	5.206E+03	1.472E+03	6.174E+02
	Std	2.182E+04	3.280E+03	4.412E+03	7.366E+02	5.524E+01
F6	Best	2.446E+03	2.247E+03	1.327E+03	1.310E+03	8.512E+02
	Mean	5.315E+04	2.691E+04	8.644E+03	5.073E+03	1.475E+03
	Std	6.258E+04	2.833E+04	7.575E+03	3.932E+03	5.336E+02
F7	Best	1.085E+03	1.232E+03	8.486E+02	8.388E+02	8.085E+02
	Mean	8.877E+03	5.183E+03	2.933E+03	1.388E+03	1.011E+03
	Std	2.156E+04	4.424E+03	2.495E+03	5.506E+02	1.493E+02
F8	Best	1.269E+03	1.017E+03	8.788E+02	1.442E+03	8.841E+02
	Mean	2.879E+04	2.937E+03	3.167E+03	5.296E+03	1.233E+03
	Std	3.584E+04	2.104E+03	5.354E+03	4.069E+03	2.781E+02
F9	Best	1.062E+03	1.301E+03	1.438E+03	1.187E+03	1.468E+03
	Mean	6.883E+04	3.634E+03	4.595E+03	3.912E+03	2.262E+03
	Std	1.498E+05	3.905E+03	3.797E+03	2.810E+03	7.450E+02
F10	Best	1.154E+03	1.784E+03	1.051E+03	1.108E+03	1.042E+03
	Mean	8.270E+03	4.698E+03	3.192E+03	1.608E+03	1.103E+03
	Std	1.445E+04	2.601E+03	4.491E+03	4.288E+02	5.289E+01
F11	Best	8.843E+03	1.017E+04	1.060E+04	1.543E+04	1.465E+04
	Mean	5.376E+05	2.187E+05	8.635E+04	6.184E+04	4.858E+04
	Std	5.461E+05	1.921E+05	7.490E+04	4.728E+04	2.538E+04
F12	Best	4.115E+04	3.750E+04	1.583E+04	5.528E+04	1.638E+04
	Mean	8.952E+05	2.609E+05	2.063E+05	2.934E+05	7.124E+04
	Std	9.181E+05	1.609E+05	2.104E+05	2.371E+05	4.467E+04
F13	Best	3.253E+04	5.434E+04	7.340E+04	1.768E+05	5.072E+03
	Mean	2.021E+06	6.049E+05	6.386E+05	7.782E+05	1.427E+04
	Std	2.523E+06	4.616E+05	7.813E+05	7.896E+05	6.107E+03
F14	Best	6.663E+04	4.900E+04	1.296E+05	8.900E+04	6.904E+04
	Mean	2.688E+06	4.314E+05	2.004E+06	2.885E+05	1.852E+05
	Std	4.576E+06	3.922E+05	2.070E+06	2.358E+05	9.053E+04
F15	Best	8.964E+03	1.108E+04	5.072E+04	7.266E+03	2.562E+03
	Mean	7.094E+05	1.524E+05	2.955E+05	2.311E+04	5.385E+03
	Std	9.194E+05	1.241E+05	2.960E+05	1.681E+04	1.646E+03
F16	Best	3.131E+04	1.884E+04	1.742E+04	6.462E+03	4.444E+03
	Mean	1.400E+06	1.287E+05	1.888E+05	5.286E+04	1.095E+04
	Std	1.660E+06	1.401E+05	2.326E+05	3.736E+04	4.669E+03
F17	Best	3.478E+04	1.648E+05	3.295E+04	1.004E+04	2.612E+04
	Mean	4.262E+06	1.408E+06	8.537E+05	1.200E+05	5.452E+04
	Std	6.285E+06	1.561E+06	9.809E+05	1.171E+05	1.890E+04
F18	Best	3.727E+04	2.351E+04	8.165E+04	4.249E+04	1.549E+04
	Mean	1.792E+06	3.316E+05	9.519E+05	2.163E+05	4.845E+04
	Std	4.405E+06	3.699E+05	1.248E+06	1.702E+05	2.493E+04
F19	Best	2.887E+05	5.325E+04	8.365E+04	5.776E+04	4.235E+04
	Mean	1.252E+07	2.650E+05	1.113E+06	2.648E+05	1.512E+05
	Std	1.304E+07	2.579E+05	1.086E+06	1.818E+05	7.264E+04
F20	Best	4.050E+04	4.600E+04	7.457E+03	2.904E+04	8.110E+03
	Mean	2.487E+06	6.988E+05	2.384E+05	1.434E+05	1.792E+04
	Std	3.741E+06	8.650E+05	2.105E+05	1.232E+05	6.950E+03
F21	Best	6.972E+03	4.208E+03	2.556E+03	3.021E+03	2.281E+03
	Mean	1.270E+05	1.526E+04	8.519E+03	5.572E+03	2.738E+03
	Std	1.822E+05	1.266E+04	5.428E+03	2.608E+03	4.133E+02
F22	Best	3.379E+03	5.797E+03	3.944E+03	3.246E+03	2.863E+03
	Mean	6.624E+04	2.962E+04	5.021E+04	7.980E+03	3.853E+03
	Std	1.250E+05	1.901E+04	6.229E+04	6.722E+03	4.937E+02
F23	Best	2.449E+04	4.162E+03	8.061E+03	6.042E+03	4.973E+03
	Mean	3.488E+05	2.184E+04	6.697E+04	3.071E+04	1.292E+04
	Std	4.594E+05	1.946E+04	1.652E+05	2.592E+04	5.074E+03
F24	Best	4.775E+03	4.111E+03	8.453E+03	7.658E+03	3.264E+03
	Mean	7.584E+04	1.747E+04	1.738E+05	2.562E+04	5.346E+03
	Std	1.059E+05	1.909E+04	2.022E+05	2.508E+04	1.519E+03
F25	Best	4.241E+03	2.868E+03	4.457E+03	5.184E+03	4.750E+03
	Mean	3.289E+05	1.164E+04	4.477E+04	1.261E+04	9.648E+03
	Std	5.267E+05	1.081E+04	5.356E+04	8.293E+03	2.583E+03
F26	Best	1.641E+04	3.914E+03	9.133E+03	3.785E+03	2.922E+03
	Mean	1.776E+05	1.479E+04	1.638E+05	2.114E+04	3.943E+03
	Std	2.632E+05	1.385E+04	2.128E+05	1.520E+04	8.389E+02
F27	Best	7.204E+03	4.858E+03	3.874E+03	3.456E+03	3.794E+03
	Mean	1.607E+05	1.154E+04	1.743E+04	8.203E+03	7.277E+03
	Std	2.144E+05	7.771E+03	1.829E+04	3.554E+03	2.348E+03
F28	Best	4.598E+03	5.774E+03	1.192E+04	4.999E+03	4.366E+03
	Mean	1.587E+05	5.103E+04	1.075E+05	2.037E+04	9.008E+03
	Std	3.629E+05	4.234E+04	1.450E+05	1.454E+04	4.489E+03
F29	Best	8.710E+03	4.980E+03	1.370E+04	6.009E+03	4.628E+03
	Mean	2.241E+05	1.730E+04	7.763E+04	1.802E+04	7.295E+03
	Std	2.951E+05	1.330E+04	7.253E+04	9.484E+03	1.712E+03
F30	Best	6.047E+03	7.758E+03	4.705E+03	5.200E+03	3.635E+03
	Mean	4.706E+04	3.791E+04	3.314E+04	1.527E+04	4.440E+03
	Std	6.451E+04	2.836E+04	3.213E+04	8.197E+03	5.551E+02

**Table A4:** Statistical results of MSSBOA with different strategies on CEC2017 (D = 100).

Func	Index	SBOA	SBOA-GPSI	SBOA-LOBL	SBOA-ACGM	MSSBOA
F1	Best	2.619E+02	1.184E+02	1.359E+02	1.024E+02	1.025E+02
	Mean	2.237E+03	3.572E+02	5.792E+02	1.060E+02	1.057E+02
	Std	2.887E+03	3.774E+02	7.105E+02	2.438E+00	2.091E+00
F3	Best	4.157E+02	3.370E+02	7.585E+02	3.069E+02	3.057E+02
	Mean	6.179E+03	1.006E+03	5.858E+03	3.179E+02	3.173E+02
	Std	1.391E+04	6.754E+02	9.920E+03	5.890E+00	6.277E+00
F4	Best	6.269E+04	3.819E+04	1.410E+04	1.027E+04	3.398E+03
	Mean	9.434E+05	6.498E+05	1.855E+05	5.781E+04	1.050E+04
	Std	1.225E+06	7.728E+05	2.147E+05	5.033E+04	4.742E+03
F5	Best	1.022E+04	2.367E+04	2.385E+04	1.248E+04	2.308E+04
	Mean	6.234E+05	2.783E+05	2.434E+05	8.098E+04	5.937E+04
	Std	9.362E+05	3.004E+05	2.842E+05	6.322E+04	2.719E+04
F6	Best	1.786E+04	9.141E+03	1.590E+04	3.774E+03	2.402E+03
	Mean	1.019E+06	6.287E+04	2.107E+05	2.358E+04	5.180E+03
	Std	3.156E+06	5.420E+04	1.699E+05	3.010E+04	2.377E+03
F7	Best	3.691E+04	1.825E+04	2.404E+04	1.537E+04	5.351E+03
	Mean	9.395E+05	2.214E+05	5.243E+05	6.095E+04	1.127E+04
	Std	1.566E+06	2.387E+05	6.229E+05	4.563E+04	5.864E+03
F8	Best	3.424E+04	1.478E+04	6.582E+04	2.610E+04	3.400E+03
	Mean	1.315E+06	1.951E+05	8.061E+05	1.059E+05	1.128E+04
	Std	1.761E+06	2.906E+05	2.258E+06	7.782E+04	5.471E+03
F9	Best	5.293E+04	3.614E+03	2.327E+03	3.543E+03	2.827E+03
	Mean	8.419E+05	2.413E+04	3.024E+04	1.941E+04	6.503E+03
	Std	1.040E+06	2.431E+04	2.968E+04	1.200E+04	2.554E+03
F10	Best	1.166E+04	8.584E+03	3.184E+04	9.792E+03	5.979E+03
	Mean	1.320E+06	1.176E+05	8.372E+05	9.823E+04	1.184E+04
	Std	2.725E+06	1.140E+05	9.423E+05	1.055E+05	6.067E+03
F11	Best	1.650E+07	3.293E+06	3.258E+07	6.620E+06	3.333E+06
	Mean	4.184E+08	3.142E+07	2.564E+08	3.322E+07	8.555E+06
	Std	5.850E+08	2.596E+07	3.452E+08	2.379E+07	4.674E+06
F12	Best	3.465E+07	7.695E+06	1.569E+07	3.182E+06	1.139E+07
	Mean	4.344E+08	5.118E+07	1.784E+08	4.518E+07	4.681E+07
	Std	3.303E+08	3.080E+07	1.782E+08	4.788E+07	2.972E+07
F13	Best	4.466E+06	6.857E+06	1.808E+06	2.384E+06	1.775E+06
	Mean	2.782E+08	3.932E+07	5.135E+07	1.624E+07	4.403E+06
	Std	9.338E+08	2.733E+07	6.522E+07	1.425E+07	2.153E+06
F14	Best	5.502E+06	3.267E+06	4.674E+06	4.364E+06	1.154E+06
	Mean	1.377E+08	5.845E+07	8.266E+07	4.779E+07	3.503E+06
	Std	2.407E+08	4.550E+07	9.932E+07	3.445E+07	1.892E+06
F15	Best	2.362E+07	2.703E+06	3.130E+06	1.042E+06	1.035E+06
	Mean	1.972E+09	4.427E+07	9.027E+07	7.005E+06	4.067E+06
	Std	2.995E+09	3.950E+07	2.539E+08	5.293E+06	2.661E+06
F16	Best	9.835E+06	1.835E+06	1.403E+07	1.011E+06	2.078E+06
	Mean	3.506E+08	2.091E+07	9.035E+07	8.144E+06	6.103E+06
	Std	4.090E+08	1.668E+07	8.202E+07	6.719E+06	3.290E+06
F17	Best	1.982E+07	2.169E+07	2.488E+07	1.983E+07	5.498E+06
	Mean	3.897E+08	1.939E+08	2.430E+08	2.333E+08	1.870E+07
	Std	5.686E+08	1.358E+08	2.949E+08	2.620E+08	1.118E+07
F18	Best	2.151E+06	4.348E+06	2.494E+06	1.131E+06	2.811E+05
	Mean	8.884E+07	3.459E+07	2.005E+07	5.865E+06	1.018E+06
	Std	1.222E+08	4.831E+07	1.643E+07	3.877E+06	6.323E+05
F19	Best	4.929E+06	1.041E+07	4.338E+06	4.191E+06	5.343E+06
	Mean	2.360E+08	9.509E+07	3.390E+08	3.529E+07	1.923E+07
	Std	2.206E+08	1.097E+08	4.316E+08	3.263E+07	1.129E+07
F20	Best	1.245E+07	1.010E+07	1.708E+07	8.055E+06	2.841E+06
	Mean	3.642E+08	6.847E+07	1.699E+08	3.271E+07	1.317E+07
	Std	4.055E+08	4.775E+07	1.490E+08	2.774E+07	6.542E+06
F21	Best	5.664E+04	9.487E+04	5.603E+05	1.369E+05	4.513E+04
	Mean	2.961E+06	1.285E+06	6.483E+06	7.535E+05	1.260E+05
	Std	1.099E+07	1.196E+06	7.721E+06	7.373E+05	6.196E+04
F22	Best	3.208E+05	2.408E+05	5.132E+05	2.601E+05	6.446E+04
	Mean	5.118E+06	3.336E+06	4.346E+06	1.380E+06	2.229E+05
	Std	7.294E+06	3.523E+06	5.640E+06	1.274E+06	1.115E+05
F23	Best	1.256E+05	4.462E+05	6.158E+04	7.037E+04	7.426E+04
	Mean	7.084E+06	4.419E+06	2.387E+06	5.861E+05	2.135E+05
	Std	1.069E+07	4.023E+06	2.114E+06	4.032E+05	9.711E+04
F24	Best	9.357E+04	4.275E+04	2.808E+05	8.943E+04	3.116E+04
	Mean	1.067E+07	4.541E+05	4.419E+06	7.062E+05	6.998E+04
	Std	1.626E+07	3.639E+05	6.177E+06	4.911E+05	3.638E+04
F25	Best	6.963E+04	2.700E+05	4.923E+05	9.546E+04	1.984E+05
	Mean	4.466E+06	2.971E+06	4.432E+06	5.864E+05	4.517E+05
	Std	4.491E+06	4.982E+06	4.306E+06	4.290E+05	1.971E+05
F26	Best	6.274E+05	1.499E+05	2.971E+05	2.648E+05	4.253E+04
	Mean	2.104E+07	1.813E+06	4.497E+06	5.202E+06	2.059E+05
	Std	3.172E+07	1.588E+06	4.411E+06	4.255E+06	7.401E+04
F27	Best	1.575E+06	9.457E+05	1.578E+06	3.757E+05	3.406E+05
	Mean	4.458E+07	1.280E+07	1.697E+07	1.352E+06	1.441E+06
	Std	5.740E+07	1.833E+07	1.571E+07	1.142E+06	8.204E+05
F28	Best	3.964E+04	1.223E+05	1.186E+05	1.724E+05	6.638E+04
	Mean	5.573E+06	2.848E+06	2.577E+06	9.180E+05	1.719E+05
	Std	7.127E+06	2.924E+06	3.607E+06	1.564E+06	8.114E+04
F29	Best	4.210E+05	5.292E+05	1.155E+05	1.306E+05	6.827E+04
	Mean	3.261E+07	2.378E+06	1.432E+06	8.785E+05	1.370E+05
	Std	3.300E+07	1.670E+06	1.359E+06	5.377E+05	6.273E+04
F30	Best	5.438E+05	2.415E+05	1.568E+05	7.308E+04	6.484E+04
	Mean	1.598E+07	2.170E+06	2.081E+06	6.800E+05	1.938E+05
	Std	2.882E+07	2.398E+06	1.804E+06	5.735E+05	8.604E+04

**Table A5:** Statistical results of MSSBOA and the comparison algorithms on CEC2017 (D = 10).

Func	Index	MSSBOA	SBOA	GWO	COA	SCA	SMA	RIME	QIO	MRFO	GKSO	LSHADE
F1	Best	1.000E+02	1.123E+02	1.192E+02	1.013E+02	1.146E+02	1.009E+02	1.011E+02	1.000E+02	1.009E+02	1.007E+02	1.000E+02
	Mean	1.000E+02	2.437E+02	4.717E+02	1.266E+02	2.006E+03	1.282E+02	1.129E+02	1.000E+02	1.185E+02	1.208E+02	1.000E+02
	Std	9.988E-03	2.198E+02	7.992E+02	2.797E+01	2.645E+03	4.094E+01	1.832E+01	1.363E-02	2.357E+01	3.053E+01	7.776E-03
	Rank	2	9	10	7	11	8	4	3	5	6	1
F3	Best	3.000E+02	3.065E+02	3.057E+02	3.008E+02	3.090E+02	3.009E+02	3.003E+02	3.000E+02	3.013E+02	3.001E+02	3.000E+02
	Mean	3.000E+02	5.150E+02	4.206E+02	3.052E+02	4.534E+02	3.119E+02	3.013E+02	3.001E+02	3.298E+02	3.013E+02	3.000E+02
	Std	5.878E-03	2.389E+02	1.625E+02	7.154E+00	1.091E+02	1.393E+01	1.345E+00	3.634E-02	3.206E+01	1.191E+00	6.296E-03
	Rank	2	11	9	6	10	7	5	3	8	4	1
F4	Best	4.003E+02	4.016E+02	4.498E+02	4.024E+02	4.428E+02	4.011E+02	4.028E+02	4.018E+02	4.005E+02	4.071E+02	4.002E+02
	Mean	4.006E+02	6.519E+02	1.763E+03	4.337E+02	9.358E+03	4.287E+02	4.407E+02	4.132E+02	4.139E+02	4.761E+02	4.011E+02
	Std	2.547E-01	4.255E+02	3.249E+03	4.308E+01	1.200E+04	2.472E+01	3.845E+01	1.243E+01	1.510E+01	7.102E+01	1.113E+00
	Rank	1	9	10	6	11	5	7	3	4	8	2
F5	Best	5.007E+02	5.057E+02	5.103E+02	5.057E+02	6.571E+02	5.032E+02	5.004E+02	5.010E+02	5.004E+02	5.007E+02	5.002E+02
	Mean	5.020E+02	7.513E+02	8.746E+02	5.345E+02	5.471E+03	5.286E+02	5.111E+02	5.043E+02	5.222E+02	5.088E+02	5.011E+02
	Std	8.921E-01	2.791E+02	5.671E+02	2.797E+01	5.851E+03	4.213E+01	1.268E+01	3.666E+00	4.699E+01	8.960E+00	8.063E-01
	Rank	2	9	10	8	11	7	5	3	6	4	1
F6	Best	6.053E+02	6.089E+02	6.171E+02	6.112E+02	6.998E+02	6.149E+02	6.004E+02	6.020E+02	6.052E+02	6.003E+02	6.005E+02
	Mean	6.117E+02	2.286E+03	9.133E+02	7.055E+02	2.352E+03	7.510E+02	6.071E+02	6.092E+02	6.649E+02	6.061E+02	6.042E+02
	Std	5.689E+00	2.248E+03	3.954E+02	1.201E+02	2.341E+03	2.295E+02	5.189E+00	5.675E+00	7.047E+01	6.502E+00	3.720E+00
	Rank	5	10	9	7	11	8	3	4	6	2	1
F7	Best	7.003E+02	7.051E+02	7.261E+02	7.010E+02	7.150E+02	7.054E+02	7.002E+02	7.001E+02	7.020E+02	7.022E+02	7.021E+02
	Mean	7.010E+02	9.364E+02	1.163E+03	7.111E+02	1.668E+03	7.863E+02	7.020E+02	7.005E+02	7.450E+02	7.257E+02	7.106E+02
	Std	4.512E-01	2.536E+02	9.356E+02	1.177E+01	1.310E+03	9.582E+01	1.644E+00	4.088E-01	5.144E+01	3.293E+01	7.860E+00
	Rank	2	9	10	5	11	8	3	1	7	6	4
F8	Best	8.002E+02	8.017E+02	8.153E+02	8.012E+02	8.485E+02	8.005E+02	8.003E+02	8.001E+02	8.016E+02	8.002E+02	8.003E+02
	Mean	8.006E+02	8.527E+02	1.212E+03	8.143E+02	1.919E+03	8.117E+02	8.023E+02	8.007E+02	8.160E+02	8.016E+02	8.016E+02
	Std	2.669E-01	6.076E+01	4.817E+02	1.661E+01	2.143E+03	1.776E+01	2.867E+00	7.013E-01	1.916E+01	1.821E+00	1.223E+00
	Rank	1	9	10	7	11	6	5	2	8	4	3
F9	Best	9.010E+02	9.405E+02	9.255E+02	9.006E+02	1.044E+03	9.056E+02	9.006E+02	9.002E+02	9.015E+02	9.002E+02	9.005E+02
	Mean	9.022E+02	1.670E+03	1.699E+03	9.064E+02	4.077E+03	9.762E+02	9.034E+02	9.016E+02	9.176E+02	9.047E+02	9.028E+02
	Std	7.769E-01	1.653E+03	2.465E+03	4.847E+00	6.578E+03	9.557E+01	2.533E+00	1.193E+00	2.036E+01	4.772E+00	1.798E+00
	Rank	2	9	10	6	11	8	4	1	7	5	3
F10	Best	1.001E+03	1.090E+03	1.041E+03	1.026E+03	1.109E+03	1.053E+03	1.004E+03	1.001E+03	1.035E+03	1.009E+03	1.001E+03
	Mean	1.003E+03	2.843E+03	1.791E+03	1.347E+03	8.689E+03	1.624E+03	1.023E+03	1.010E+03	1.371E+03	1.096E+03	1.008E+03
	Std	1.400E+00	2.390E+03	8.171E+02	4.086E+02	1.196E+04	7.124E+02	1.912E+01	7.795E+00	4.809E+02	1.172E+02	7.567E+00
	Rank	1	10	9	6	11	8	4	3	7	5	2
F11	Best	1.105E+03	1.195E+03	2.113E+03	1.117E+03	2.359E+03	1.217E+03	1.111E+03	1.104E+03	1.150E+03	1.115E+03	1.136E+03
	Mean	1.110E+03	5.314E+03	1.746E+04	1.452E+03	3.357E+04	3.842E+03	1.178E+03	1.134E+03	3.286E+03	1.291E+03	1.313E+03
	Std	3.684E+00	4.233E+03	1.970E+04	5.140E+02	4.317E+04	3.767E+03	5.776E+01	4.033E+01	2.495E+03	2.168E+02	2.075E+02
	Rank	1	9	10	6	11	8	3	2	7	4	5
F12	Best	1.211E+03	1.389E+03	1.537E+03	1.218E+03	1.404E+03	1.219E+03	1.202E+03	1.201E+03	1.310E+03	1.206E+03	1.216E+03
	Mean	1.221E+03	1.036E+04	6.966E+03	1.323E+03	1.164E+04	1.313E+03	1.221E+03	1.208E+03	1.757E+03	1.300E+03	1.248E+03
	Std	5.425E+00	2.026E+04	8.114E+03	1.249E+02	1.135E+04	8.792E+01	2.024E+01	4.737E+00	5.864E+02	9.427E+01	3.538E+01
	Rank	2	10	9	7	11	6	3	1	8	5	4
F13	Best	1.308E+03	1.476E+03	2.572E+03	1.307E+03	1.802E+03	1.328E+03	1.315E+03	1.303E+03	1.372E+03	1.310E+03	1.306E+03
	Mean	1.318E+03	4.542E+03	1.014E+04	1.536E+03	1.453E+04	1.645E+03	1.381E+03	1.350E+03	2.138E+03	1.482E+03	1.330E+03
	Std	9.953E+00	6.060E+03	1.041E+04	1.808E+02	1.534E+04	4.306E+02	5.770E+01	3.673E+01	1.418E+03	2.200E+02	2.093E+01
	Rank	1	9	10	6	11	7	4	3	8	5	2
F14	Best	1.401E+03	1.459E+03	1.467E+03	1.449E+03	1.548E+03	1.405E+03	1.403E+03	1.404E+03	1.417E+03	1.412E+03	1.413E+03
	Mean	1.405E+03	9.457E+03	2.365E+03	1.708E+03	1.636E+04	1.486E+03	1.418E+03	1.426E+03	1.549E+03	1.529E+03	1.447E+03
	Std	2.081E+00	1.880E+04	1.041E+03	3.202E+02	1.946E+04	1.067E+02	1.300E+01	2.205E+01	1.627E+02	1.118E+02	3.510E+01
	Rank	1	10	9	8	11	5	2	3	7	6	4
F15	Best	1.505E+03	1.504E+03	1.618E+03	1.512E+03	1.759E+03	1.503E+03	1.504E+03	1.502E+03	1.511E+03	1.504E+03	1.502E+03
	Mean	1.510E+03	1.737E+03	3.616E+03	1.674E+03	1.707E+04	1.582E+03	1.539E+03	1.517E+03	1.567E+03	1.535E+03	1.508E+03
	Std	3.941E+00	4.092E+02	3.066E+03	2.220E+02	2.221E+04	9.671E+01	3.618E+01	1.817E+01	4.993E+01	4.273E+01	6.487E+00
	Rank	2	9	10	8	11	7	5	3	6	4	1
F16	Best	1.605E+03	1.614E+03	1.984E+03	1.628E+03	1.738E+03	1.618E+03	1.621E+03	1.614E+03	1.612E+03	1.609E+03	1.614E+03
	Mean	1.611E+03	3.227E+03	1.034E+04	2.013E+03	1.224E+04	1.898E+03	1.690E+03	1.664E+03	2.140E+03	1.708E+03	1.675E+03
	Std	4.934E+00	2.042E+03	1.774E+04	3.974E+02	1.999E+04	3.608E+02	5.905E+01	4.450E+01	6.666E+02	9.345E+01	4.953E+01
	Rank	1	9	10	7	11	6	4	2	8	5	3
F17	Best	1.704E+03	1.717E+03	1.784E+03	1.706E+03	1.916E+03	1.716E+03	1.703E+03	1.701E+03	1.731E+03	1.703E+03	1.704E+03
	Mean	1.707E+03	2.382E+03	3.138E+03	1.956E+03	5.108E+03	1.998E+03	1.749E+03	1.712E+03	1.958E+03	1.780E+03	1.729E+03
	Std	2.837E+00	8.673E+02	1.408E+03	3.552E+02	3.470E+03	4.406E+02	3.383E+01	9.554E+00	2.593E+02	6.322E+01	1.889E+01
	Rank	1	9	10	6	11	8	4	2	7	5	3
F18	Best	1.803E+03	1.815E+03	1.866E+03	1.835E+03	1.834E+03	1.822E+03	1.803E+03	1.801E+03	1.815E+03	1.803E+03	1.804E+03
	Mean	1.808E+03	2.402E+03	3.322E+03	2.060E+03	4.300E+03	2.011E+03	1.816E+03	1.808E+03	1.926E+03	1.865E+03	1.822E+03
	Std	3.643E+00	7.276E+02	2.182E+03	2.066E+02	3.981E+03	2.242E+02	1.194E+01	8.737E+00	1.253E+02	7.185E+01	1.130E+01
	Rank	1	9	10	8	11	7	3	2	6	5	4
F19	Best	1.914E+03	1.997E+03	1.984E+03	1.909E+03	3.000E+03	1.929E+03	1.917E+03	1.936E+03	1.963E+03	1.906E+03	1.904E+03
	Mean	1.929E+03	8.301E+03	4.158E+03	2.274E+03	2.737E+04	2.136E+03	2.043E+03	2.165E+03	2.618E+03	1.943E+03	1.955E+03
	Std	1.139E+01	1.154E+04	2.875E+03	5.164E+02	2.867E+04	1.831E+02	1.442E+02	1.665E+02	1.061E+03	3.406E+01	3.882E+01
	Rank	1	10	9	7	11	5	4	6	8	2	3
F20	Best	2.002E+03	2.076E+03	2.079E+03	2.003E+03	2.136E+03	2.026E+03	2.003E+03	2.011E+03	2.012E+03	2.011E+03	2.003E+03
	Mean	2.007E+03	3.747E+03	7.259E+03	2.069E+03	4.768E+03	2.531E+03	2.016E+03	2.057E+03	2.085E+03	2.095E+03	2.016E+03
	Std	4.172E+00	2.163E+03	8.584E+03	9.776E+01	4.094E+03	7.338E+02	1.746E+01	3.740E+01	8.998E+01	1.015E+02	1.110E+01
	Rank	1	9	11	5	10	8	3	4	6	7	2
F21	Best	2.106E+03	2.145E+03	2.122E+03	2.112E+03	2.313E+03	2.187E+03	2.107E+03	2.103E+03	2.111E+03	2.109E+03	2.107E+03
	Mean	2.111E+03	3.717E+03	4.973E+03	2.190E+03	4.834E+03	2.728E+03	2.160E+03	2.120E+03	2.195E+03	2.238E+03	2.147E+03
	Std	3.788E+00	1.532E+03	7.218E+03	1.005E+02	4.933E+03	6.194E+02	5.600E+01	1.571E+01	8.561E+01	1.885E+02	3.879E+01
	Rank	1	9	11	5	10	8	4	2	6	7	3
F22	Best	2.200E+03	2.212E+03	2.275E+03	2.203E+03	2.223E+03	2.271E+03	2.201E+03	2.201E+03	2.203E+03	2.203E+03	2.202E+03
	Mean	2.201E+03	2.563E+03	4.489E+03	2.275E+03	3.977E+03	3.966E+03	2.212E+03	2.205E+03	2.231E+03	2.232E+03	2.209E+03
	Std	3.075E-01	3.806E+02	2.858E+03	7.409E+01	3.671E+03	3.338E+03	1.118E+01	4.162E+00	2.183E+01	4.867E+01	6.951E+00
	Rank	1	8	11	7	10	9	4	2	5	6	3
F23	Best	2.301E+03	2.318E+03	2.311E+03	2.301E+03	3.161E+03	2.324E+03	2.303E+03	2.302E+03	2.314E+03	2.320E+03	2.305E+03
	Mean	2.302E+03	3.159E+03	2.562E+03	2.329E+03	3.179E+04	2.552E+03	2.331E+03	2.313E+03	2.448E+03	2.440E+03	2.320E+03
	Std	9.032E-01	9.871E+02	6.840E+02	2.555E+01	3.726E+04	2.591E+02	2.151E+01	1.066E+01	1.730E+02	1.129E+02	1.196E+01
	Rank	1	10	9	4	11	8	5	2	7	6	3
F24	Best	2.400E+03	2.407E+03	2.406E+03	2.401E+03	2.426E+03	2.401E+03	2.400E+03	2.401E+03	2.400E+03	2.404E+03	2.401E+03
	Mean	2.401E+03	2.793E+03	2.537E+03	2.419E+03	3.499E+03	2.425E+03	2.415E+03	2.406E+03	2.406E+03	2.425E+03	2.405E+03
	Std	3.039E-01	8.087E+02	2.485E+02	2.248E+01	1.644E+03	6.803E+01	1.605E+01	4.427E+00	7.309E+00	2.739E+01	3.055E+00
	Rank	1	10	9	6	11	7	5	3	4	8	2
F25	Best	2.501E+03	2.510E+03	2.521E+03	2.503E+03	2.583E+03	2.501E+03	2.502E+03	2.500E+03	2.526E+03	2.502E+03	2.512E+03
	Mean	2.502E+03	3.020E+03	3.059E+03	2.522E+03	4.213E+03	2.549E+03	2.533E+03	2.502E+03	2.726E+03	2.534E+03	2.546E+03
	Std	6.844E-01	1.262E+03	1.479E+03	2.509E+01	3.053E+03	6.596E+01	4.844E+01	2.174E+00	2.063E+02	3.965E+01	3.478E+01
	Rank	1	9	10	3	11	7	4	2	8	5	6
F26	Best	2.601E+03	2.645E+03	2.638E+03	2.614E+03	2.778E+03	2.603E+03	2.601E+03	2.601E+03	2.608E+03	2.603E+03	2.601E+03
	Mean	2.603E+03	3.318E+03	3.026E+03	2.715E+03	5.586E+03	2.676E+03	2.614E+03	2.609E+03	2.789E+03	2.666E+03	2.604E+03
	Std	1.107E+00	7.665E+02	3.161E+02	1.153E+02	3.481E+03	1.115E+02	1.816E+01	7.998E+00	2.281E+02	1.086E+02	2.600E+00
	Rank	1	10	9	7	11	6	4	3	8	5	2
F27	Best	2.701E+03	2.707E+03	2.713E+03	2.708E+03	2.737E+03	2.703E+03	2.701E+03	2.701E+03	2.710E+03	2.701E+03	2.705E+03
	Mean	2.702E+03	2.872E+03	3.111E+03	2.790E+03	4.884E+03	2.775E+03	2.703E+03	2.704E+03	2.942E+03	2.705E+03	2.721E+03
	Std	7.579E-01	3.266E+02	5.479E+02	7.113E+01	3.908E+03	7.525E+01	2.082E+00	3.062E+00	3.126E+02	6.126E+00	2.150E+01
	Rank	1	8	10	7	11	6	2	3	9	4	5
F28	Best	2.804E+03	2.925E+03	2.896E+03	2.807E+03	2.926E+03	2.808E+03	2.805E+03	2.806E+03	2.904E+03	2.806E+03	2.818E+03
	Mean	2.813E+03	4.840E+03	4.973E+03	2.895E+03	5.632E+03	3.117E+03	2.854E+03	2.825E+03	3.854E+03	2.837E+03	2.868E+03
	Std	4.176E+00	2.701E+03	3.172E+03	1.295E+02	6.627E+03	6.663E+02	4.745E+01	2.163E+01	1.411E+03	3.507E+01	4.297E+01
	Rank	1	9	10	6	11	7	4	2	8	3	5
F29	Best	2.901E+03	2.952E+03	3.016E+03	2.902E+03	3.040E+03	2.951E+03	2.926E+03	2.903E+03	2.923E+03	2.907E+03	2.921E+03
	Mean	2.903E+03	7.193E+03	4.678E+03	2.945E+03	5.930E+03	3.276E+03	3.018E+03	2.929E+03	3.273E+03	2.967E+03	3.015E+03
	Std	9.906E-01	1.041E+04	2.396E+03	5.434E+01	6.601E+03	5.683E+02	8.671E+01	2.686E+01	4.634E+02	1.072E+02	8.135E+01
	Rank	1	11	9	3	10	8	6	2	7	4	5
F30	Best	3.010E+03	3.004E+03	3.021E+03	3.018E+03	3.149E+03	3.013E+03	3.001E+03	3.001E+03	3.036E+03	3.013E+03	3.010E+03
	Mean	3.021E+03	3.971E+03	3.739E+03	3.231E+03	1.082E+04	3.180E+03	3.008E+03	3.007E+03	3.498E+03	3.101E+03	3.046E+03
	Std	9.410E+00	1.393E+03	1.061E+03	3.483E+02	9.813E+03	1.499E+02	5.570E+00	5.101E+00	4.725E+02	8.567E+01	2.453E+01
	Rank	3	10	9	7	11	6	2	1	8	5	4

**Table A6:** Statistical results of MSSBOA and the comparison algorithms on CEC2017 (D = 30).

Func	Index	MSSBOA	SBOA	GWO	COA	SCA	SMA	RIME	QIO	MRFO	GKSO	LSHADE
F1	Best	1.000E+02	1.290E+02	1.103E+02	1.018E+02	1.990E+02	1.107E+02	1.009E+02	1.001E+02	1.059E+02	1.051E+02	1.000E+02
	Mean	1.000E+02	3.029E+03	3.795E+02	1.339E+02	3.076E+03	2.063E+02	1.082E+02	1.003E+02	2.558E+02	1.408E+02	1.001E+02
	Std	1.057E-02	3.601E+03	3.535E+02	3.977E+01	5.188E+03	1.177E+02	7.737E+00	1.166E-01	2.197E+02	6.254E+01	4.861E-02
	Rank	1	10	9	5	11	7	4	3	8	6	2
F3	Best	3.000E+02	3.048E+02	3.003E+02	3.037E+02	3.155E+02	3.004E+02	3.001E+02	3.000E+02	3.047E+02	3.009E+02	3.000E+02
	Mean	3.000E+02	6.992E+02	3.644E+02	3.379E+02	9.083E+02	3.220E+02	3.023E+02	3.000E+02	3.449E+02	3.139E+02	3.000E+02
	Std	4.932E-03	6.319E+02	8.164E+01	5.453E+01	7.303E+02	3.088E+01	4.161E+00	1.404E-02	4.929E+01	1.422E+01	5.316E-03
	Rank	2	10	9	7	11	6	4	3	8	5	1
F4	Best	4.024E+02	6.616E+02	4.962E+02	4.105E+02	4.538E+02	4.328E+02	4.190E+02	4.016E+02	4.030E+02	4.164E+02	4.055E+02
	Mean	4.112E+02	1.176E+04	7.117E+03	5.215E+02	1.385E+04	1.066E+03	5.159E+02	4.121E+02	5.206E+02	5.286E+02	4.231E+02
	Std	6.650E+00	1.938E+04	1.112E+04	2.031E+02	1.382E+04	7.423E+02	1.070E+02	7.346E+00	1.717E+02	1.195E+02	1.498E+01
	Rank	1	10	9	6	11	8	4	2	5	7	3
F5	Best	5.020E+02	1.432E+03	5.940E+02	5.031E+02	7.088E+02	5.036E+02	5.029E+02	5.025E+02	5.080E+02	5.161E+02	5.005E+02
	Mean	5.042E+02	3.413E+04	8.144E+03	5.696E+02	5.943E+03	6.130E+02	5.244E+02	5.230E+02	5.629E+02	6.555E+02	5.016E+02
	Std	1.635E+00	4.865E+04	1.328E+04	1.038E+02	8.165E+03	2.503E+02	2.460E+01	1.791E+01	4.630E+01	1.606E+02	7.600E-01
	Rank	2	11	10	6	9	7	4	3	5	8	1
F6	Best	6.017E+02	6.344E+02	6.097E+02	6.094E+02	7.378E+02	6.027E+02	6.041E+02	6.009E+02	6.124E+02	6.033E+02	6.010E+02
	Mean	6.028E+02	1.431E+03	1.381E+03	6.730E+02	3.806E+03	6.494E+02	6.191E+02	6.055E+02	7.203E+02	6.415E+02	6.047E+02
	Std	7.343E-01	9.189E+02	9.280E+02	6.950E+01	3.439E+03	7.445E+01	1.698E+01	4.241E+00	1.157E+02	4.837E+01	4.487E+00
	Rank	1	10	9	7	11	6	4	3	8	5	2
F7	Best	7.012E+02	7.166E+02	7.396E+02	7.060E+02	1.366E+03	7.108E+02	7.054E+02	7.031E+02	7.227E+02	7.153E+02	7.061E+02
	Mean	7.025E+02	1.499E+03	1.751E+03	7.406E+02	2.668E+04	8.244E+02	7.755E+02	7.171E+02	8.909E+02	8.532E+02	7.347E+02
	Std	9.887E-01	9.159E+02	1.341E+03	3.969E+01	5.500E+04	1.810E+02	6.786E+01	1.329E+01	2.375E+02	2.421E+02	2.044E+01
	Rank	1	9	10	4	11	6	5	2	8	7	3
F8	Best	8.033E+02	8.525E+02	8.971E+02	8.166E+02	1.486E+03	8.402E+02	8.138E+02	8.381E+02	8.249E+02	8.295E+02	8.066E+02
	Mean	8.158E+02	6.655E+03	3.571E+03	1.257E+03	2.824E+04	1.356E+03	8.820E+02	1.012E+03	9.714E+02	1.033E+03	8.232E+02
	Std	8.708E+00	1.746E+04	3.657E+03	5.063E+02	6.417E+04	1.190E+03	1.396E+02	2.175E+02	1.282E+02	2.941E+02	2.073E+01
	Rank	1	10	9	7	11	8	3	5	4	6	2
F9	Best	9.044E+02	1.187E+03	1.047E+03	9.162E+02	1.418E+03	9.163E+02	9.029E+02	9.058E+02	9.405E+02	9.064E+02	9.106E+02
	Mean	9.159E+02	8.479E+03	4.589E+03	9.914E+02	1.967E+04	1.030E+03	9.433E+02	9.479E+02	1.285E+03	9.916E+02	9.584E+02
	Std	7.852E+00	1.240E+04	3.470E+03	1.127E+02	3.601E+04	2.221E+02	5.671E+01	5.316E+01	4.281E+02	1.531E+02	4.625E+01
	Rank	1	10	9	5	11	7	2	3	8	6	4
F10	Best	1.003E+03	1.395E+03	1.232E+03	1.033E+03	1.196E+03	1.047E+03	1.005E+03	1.018E+03	1.012E+03	1.060E+03	1.011E+03
	Mean	1.006E+03	1.386E+04	4.228E+03	1.412E+03	2.001E+04	2.095E+03	1.060E+03	1.088E+03	1.125E+03	1.619E+03	1.085E+03
	Std	2.547E+00	2.393E+04	3.480E+03	3.267E+02	3.549E+04	1.199E+03	5.749E+01	5.300E+01	8.620E+01	5.607E+02	6.194E+01
	Rank	1	10	9	6	11	8	2	4	5	7	3
F11	Best	1.242E+03	1.692E+03	3.750E+03	1.384E+03	1.015E+04	1.977E+03	1.172E+03	1.257E+03	2.297E+03	1.947E+03	1.500E+03
	Mean	1.497E+03	3.220E+04	8.584E+04	4.837E+03	3.933E+05	1.192E+04	1.768E+03	2.289E+03	2.211E+04	7.670E+03	3.592E+03
	Std	1.429E+02	5.848E+04	1.606E+05	3.327E+03	7.153E+05	1.163E+04	6.768E+02	9.355E+02	3.191E+04	9.219E+03	1.910E+03
	Rank	1	9	10	5	11	7	2	3	8	6	4
F12	Best	1.417E+03	2.399E+03	9.658E+03	1.857E+03	1.142E+04	1.728E+03	3.155E+03	1.513E+03	2.225E+03	1.461E+03	1.707E+03
	Mean	1.770E+03	2.170E+05	2.380E+05	1.257E+04	1.609E+05	5.853E+03	1.208E+04	3.442E+03	1.508E+04	5.564E+03	2.976E+03
	Std	1.925E+02	3.127E+05	4.990E+05	1.133E+04	1.527E+05	6.059E+03	1.127E+04	1.608E+03	1.500E+04	6.123E+03	1.455E+03
	Rank	1	10	11	7	9	5	6	3	8	4	2
F13	Best	1.327E+03	1.561E+03	2.260E+03	1.324E+03	1.706E+04	1.315E+03	1.410E+03	1.325E+03	1.503E+03	1.385E+03	1.593E+03
	Mean	1.360E+03	1.209E+04	7.346E+04	1.927E+03	4.047E+05	2.213E+03	2.138E+03	1.487E+03	3.849E+03	3.223E+03	2.237E+03
	Std	2.292E+01	1.704E+04	1.129E+05	1.098E+03	5.836E+05	6.857E+02	1.225E+03	1.522E+02	2.210E+03	1.932E+03	6.129E+02
	Rank	1	9	10	3	11	5	4	2	8	7	6
F14	Best	1.451E+03	3.215E+03	2.426E+03	1.712E+03	3.273E+04	2.444E+03	1.548E+03	2.076E+03	2.066E+03	1.689E+03	1.624E+03
	Mean	1.516E+03	6.374E+04	5.309E+04	7.210E+03	5.910E+05	2.133E+04	2.561E+03	4.848E+03	1.811E+04	3.777E+03	2.287E+03
	Std	3.862E+01	1.293E+05	6.741E+04	9.879E+03	7.380E+05	4.049E+04	1.086E+03	2.589E+03	1.592E+04	1.986E+03	5.255E+02
	Rank	1	10	9	6	11	8	3	5	7	4	2
F15	Best	2.034E+03	1.899E+03	6.437E+03	1.597E+03	4.058E+03	1.727E+03	1.642E+03	1.677E+03	2.122E+03	1.707E+03	1.557E+03
	Mean	2.543E+03	3.263E+04	9.545E+04	5.834E+03	3.269E+05	9.868E+03	2.804E+03	3.297E+03	8.797E+03	6.964E+03	1.722E+03
	Std	3.751E+02	5.439E+04	1.181E+05	6.743E+03	4.691E+05	8.610E+03	1.576E+03	1.935E+03	1.158E+04	5.874E+03	2.188E+02
	Rank	2	9	10	5	11	8	3	4	7	6	1
F16	Best	1.748E+03	2.847E+03	3.677E+03	1.736E+03	5.435E+03	1.655E+03	1.686E+03	1.877E+03	1.704E+03	1.791E+03	1.746E+03
	Mean	2.014E+03	5.208E+04	6.810E+04	8.406E+03	1.552E+05	2.373E+03	2.749E+03	3.761E+03	2.919E+03	3.208E+03	2.616E+03
	Std	1.491E+02	6.207E+04	1.462E+05	1.003E+04	2.607E+05	7.304E+02	1.088E+03	1.516E+03	1.397E+03	2.063E+03	6.743E+02
	Rank	1	9	10	8	11	2	4	7	5	6	3
F17	Best	1.911E+03	1.830E+04	2.821E+04	3.761E+03	1.545E+05	2.576E+03	2.035E+03	2.440E+03	2.951E+03	2.729E+03	2.027E+03
	Mean	2.250E+03	7.186E+05	1.123E+06	4.142E+04	1.838E+06	1.129E+04	4.212E+03	1.101E+04	1.585E+04	1.294E+04	3.426E+03
	Std	2.304E+02	8.540E+05	1.195E+06	4.289E+04	2.090E+06	8.890E+03	2.109E+03	7.187E+03	1.661E+04	9.730E+03	1.344E+03
	Rank	1	9	10	8	11	5	3	4	7	6	2
F18	Best	2.879E+03	2.106E+03	5.215E+03	3.302E+03	2.351E+04	2.669E+03	2.180E+03	2.061E+03	4.039E+03	2.233E+03	2.601E+03
	Mean	4.169E+03	1.164E+04	1.031E+05	1.235E+04	7.370E+05	1.290E+04	4.904E+03	4.220E+03	2.350E+04	7.790E+03	5.263E+03
	Std	9.080E+02	1.287E+04	1.171E+05	1.089E+04	9.333E+05	1.637E+04	3.688E+03	3.945E+03	2.396E+04	4.645E+03	2.410E+03
	Rank	1	6	10	7	11	8	3	2	9	5	4
F19	Best	2.172E+03	7.397E+03	3.177E+03	2.208E+03	3.775E+04	2.364E+03	2.051E+03	2.131E+03	4.244E+03	1.937E+03	2.131E+03
	Mean	2.540E+03	1.079E+05	2.246E+04	8.488E+03	3.521E+05	9.682E+03	2.698E+03	3.530E+03	4.549E+04	2.761E+03	2.756E+03
	Std	2.432E+02	1.967E+05	2.124E+04	8.053E+03	4.712E+05	9.742E+03	6.237E+02	1.663E+03	4.125E+04	6.643E+02	5.701E+02
	Rank	1	10	8	6	11	7	2	5	9	4	3
F20	Best	2.045E+03	2.388E+03	1.475E+04	3.991E+03	6.823E+03	4.886E+03	2.255E+03	2.492E+03	2.621E+03	2.297E+03	2.613E+03
	Mean	2.121E+03	1.711E+05	1.545E+05	2.904E+04	2.302E+05	2.715E+04	3.619E+03	8.841E+03	9.247E+03	8.294E+03	4.864E+03
	Std	4.384E+01	2.083E+05	2.167E+05	2.399E+04	3.926E+05	3.686E+04	1.786E+03	5.518E+03	8.859E+03	8.000E+03	1.791E+03
	Rank	1	10	9	8	11	7	2	5	6	4	3
F21	Best	2.145E+03	2.329E+03	2.149E+03	3.218E+03	3.498E+03	2.508E+03	2.147E+03	2.129E+03	2.181E+03	2.278E+03	2.213E+03
	Mean	2.217E+03	1.330E+04	1.552E+04	1.240E+04	6.650E+04	8.335E+03	2.549E+03	2.331E+03	4.908E+03	3.337E+03	2.862E+03
	Std	4.732E+01	1.499E+04	2.289E+04	1.303E+04	1.173E+05	6.663E+03	3.575E+02	1.460E+02	4.385E+03	1.083E+03	6.504E+02
	Rank	1	9	10	8	11	7	3	2	6	5	4
F22	Best	2.216E+03	3.298E+03	2.634E+03	2.218E+03	2.571E+03	2.256E+03	2.205E+03	2.210E+03	2.335E+03	2.282E+03	2.283E+03
	Mean	2.240E+03	2.024E+04	9.975E+03	2.914E+03	1.185E+04	3.883E+03	2.284E+03	2.245E+03	3.400E+03	2.678E+03	2.404E+03
	Std	1.841E+01	2.265E+04	8.655E+03	6.689E+02	1.164E+04	2.360E+03	9.729E+01	4.094E+01	1.119E+03	4.010E+02	1.362E+02
	Rank	1	11	9	6	10	8	3	2	7	5	4
F23	Best	2.341E+03	3.066E+03	3.049E+03	2.498E+03	5.280E+03	2.506E+03	2.435E+03	2.524E+03	2.926E+03	2.508E+03	2.360E+03
	Mean	2.416E+03	1.639E+04	4.706E+04	4.954E+03	1.224E+05	5.176E+03	3.158E+03	3.149E+03	1.788E+04	4.132E+03	2.650E+03
	Std	3.936E+01	1.518E+04	6.558E+04	2.892E+03	1.650E+05	3.254E+03	1.014E+03	6.753E+02	1.979E+04	1.653E+03	3.044E+02
	Rank	1	8	10	6	11	7	4	3	9	5	2
F24	Best	2.418E+03	2.440E+03	2.965E+03	2.480E+03	9.487E+03	2.510E+03	2.449E+03	2.408E+03	2.440E+03	2.500E+03	2.531E+03
	Mean	2.444E+03	7.054E+03	1.790E+04	3.668E+03	1.131E+05	4.784E+03	3.043E+03	2.476E+03	2.894E+03	3.262E+03	3.285E+03
	Std	1.578E+01	7.963E+03	2.746E+04	1.566E+03	1.147E+05	2.467E+03	8.157E+02	6.131E+01	5.484E+02	8.743E+02	6.895E+02
	Rank	1	9	10	7	11	8	4	2	3	5	6
F25	Best	2.513E+03	2.521E+03	2.617E+03	2.514E+03	4.746E+03	2.520E+03	2.518E+03	2.517E+03	2.595E+03	2.604E+03	2.527E+03
	Mean	2.526E+03	6.183E+03	7.150E+03	2.810E+03	2.457E+04	2.855E+03	2.771E+03	2.591E+03	2.980E+03	3.267E+03	2.591E+03
	Std	8.720E+00	3.821E+03	5.552E+03	3.278E+02	2.522E+04	4.370E+02	4.760E+02	7.952E+01	6.060E+02	1.027E+03	7.531E+01
	Rank	1	9	10	5	11	6	4	3	7	8	2
F26	Best	2.691E+03	2.938E+03	2.954E+03	2.699E+03	5.871E+03	2.750E+03	2.743E+03	2.617E+03	2.671E+03	2.716E+03	2.673E+03
	Mean	2.776E+03	8.194E+03	1.090E+04	3.235E+03	1.155E+05	6.298E+03	3.342E+03	2.778E+03	3.137E+03	4.000E+03	2.849E+03
	Std	6.933E+01	6.869E+03	1.348E+04	6.092E+02	1.229E+05	5.321E+03	5.963E+02	1.152E+02	4.494E+02	1.847E+03	1.232E+02
	Rank	1	9	10	5	11	8	6	2	4	7	3
F27	Best	2.712E+03	2.742E+03	2.770E+03	2.763E+03	5.809E+03	2.814E+03	2.714E+03	2.711E+03	3.431E+03	2.782E+03	2.739E+03
	Mean	2.728E+03	4.929E+03	4.429E+03	3.768E+03	1.323E+05	4.512E+03	2.851E+03	2.777E+03	6.957E+03	3.472E+03	2.866E+03
	Std	1.229E+01	2.953E+03	1.925E+03	1.222E+03	2.515E+05	2.984E+03	2.187E+02	6.933E+01	3.885E+03	7.499E+02	1.092E+02
	Rank	1	9	7	6	11	8	3	2	10	5	4
F28	Best	2.817E+03	2.997E+03	3.899E+03	2.832E+03	6.814E+03	3.012E+03	2.857E+03	2.818E+03	3.404E+03	2.925E+03	2.896E+03
	Mean	2.833E+03	1.328E+04	3.584E+04	3.183E+03	1.867E+05	5.694E+03	3.330E+03	2.922E+03	9.254E+03	3.890E+03	3.240E+03
	Std	1.102E+01	1.636E+04	4.707E+04	4.817E+02	1.983E+05	3.261E+03	5.722E+02	8.202E+01	9.166E+03	1.070E+03	2.495E+02
	Rank	1	9	10	3	11	7	5	2	8	6	4
F29	Best	2.918E+03	3.406E+03	3.428E+03	3.108E+03	4.855E+03	3.012E+03	2.931E+03	2.968E+03	2.991E+03	2.928E+03	2.935E+03
	Mean	2.937E+03	1.282E+04	1.013E+04	5.377E+03	1.494E+05	3.649E+03	3.017E+03	3.398E+03	3.919E+03	3.229E+03	3.041E+03
	Std	1.465E+01	1.494E+04	6.730E+03	2.764E+03	1.622E+05	5.064E+02	8.956E+01	5.042E+02	1.320E+03	3.318E+02	8.102E+01
	Rank	1	10	9	8	11	6	2	5	7	4	3
F30	Best	3.065E+03	3.105E+03	3.060E+03	3.677E+03	4.919E+03	3.204E+03	3.026E+03	3.036E+03	3.138E+03	3.088E+03	3.052E+03
	Mean	3.145E+03	1.926E+04	1.194E+04	1.088E+04	5.506E+04	6.073E+03	3.318E+03	3.261E+03	4.397E+03	4.126E+03	3.364E+03
	Std	5.116E+01	3.064E+04	1.146E+04	1.083E+04	7.552E+04	5.250E+03	4.679E+02	2.173E+02	1.100E+03	7.870E+02	2.318E+02
	Rank	1	10	9	8	11	7	3	2	6	5	4

**Table A7:** Statistical results of MSSBOA and the comparison algorithms on CEC2017 (D = 50).

Func	Index	MSSBOA	SBOA	GWO	COA	SCA	SMA	RIME	QIO	MRFO	GKSO	LSHADE
F1	Best	1.000E+02	1.342E+02	1.114E+02	1.203E+02	1.379E+02	1.211E+02	1.019E+02	1.000E+02	1.043E+02	1.021E+02	1.001E+02
	Mean	1.001E+02	9.218E+02	6.561E+02	2.759E+02	4.836E+02	2.486E+02	1.156E+02	1.001E+02	1.800E+02	1.490E+02	1.003E+02
	Std	2.373E-02	1.243E+03	9.273E+02	1.571E+02	4.846E+02	1.503E+02	1.477E+01	4.977E-02	1.038E+02	5.223E+01	1.395E-01
	Rank	1	11	10	8	9	7	4	2	6	5	3
F3	Best	3.001E+02	4.805E+02	3.948E+02	3.112E+02	5.718E+02	3.116E+02	3.031E+02	3.002E+02	3.251E+02	3.071E+02	3.001E+02
	Mean	3.002E+02	2.894E+03	1.610E+03	4.647E+02	9.834E+03	4.142E+02	3.170E+02	3.006E+02	6.049E+02	3.683E+02	3.002E+02
	Std	7.062E-02	2.947E+03	1.704E+03	1.749E+02	2.122E+04	1.527E+02	1.504E+01	2.440E-01	3.244E+02	4.957E+01	8.264E-02
	Rank	1	10	9	7	11	6	4	3	8	5	2
F4	Best	4.147E+02	7.282E+02	1.480E+03	4.344E+02	2.209E+03	4.743E+02	4.226E+02	4.840E+02	5.308E+02	7.160E+02	4.151E+02
	Mean	4.364E+02	2.683E+04	1.092E+04	1.429E+03	1.120E+05	1.501E+03	5.830E+02	8.420E+02	1.732E+03	2.950E+03	5.029E+02
	Std	1.316E+01	4.297E+04	7.616E+03	1.149E+03	1.560E+05	1.584E+03	1.859E+02	3.982E+02	1.078E+03	2.989E+03	8.378E+01
	Rank	1	10	9	5	11	6	3	4	7	8	2
F5	Best	5.207E+02	8.328E+02	1.490E+03	5.994E+02	2.727E+03	7.958E+02	5.502E+02	5.152E+02	8.289E+02	6.588E+02	5.213E+02
	Mean	5.521E+02	9.753E+04	1.183E+04	3.895E+03	4.997E+04	6.364E+03	8.706E+02	5.941E+02	4.392E+03	1.660E+03	5.939E+02
	Std	2.402E+01	2.713E+05	1.934E+04	4.891E+03	4.911E+04	5.802E+03	3.421E+02	7.040E+01	4.092E+03	1.038E+03	5.896E+01
	Rank	1	11	9	6	10	8	4	3	7	5	2
F6	Best	6.086E+02	2.620E+03	1.780E+03	7.182E+02	1.324E+04	6.479E+02	6.196E+02	6.254E+02	7.018E+02	6.542E+02	6.603E+02
	Mean	6.414E+02	2.108E+04	2.319E+04	1.568E+03	1.979E+05	1.220E+03	7.660E+02	8.405E+02	4.195E+03	1.290E+03	8.984E+02
	Std	1.381E+01	1.938E+04	3.023E+04	1.931E+03	2.392E+05	6.658E+02	1.512E+02	1.721E+02	4.755E+03	8.367E+02	2.275E+02
	Rank	1	9	10	7	11	5	2	3	8	6	4
F7	Best	7.137E+02	8.397E+02	3.688E+03	7.511E+02	2.108E+04	1.531E+03	7.181E+02	7.944E+02	1.105E+03	7.930E+02	9.286E+02
	Mean	7.342E+02	2.241E+04	9.600E+04	1.321E+03	2.702E+05	1.493E+04	8.494E+02	1.184E+03	1.007E+04	1.248E+03	1.386E+03
	Std	1.436E+01	4.877E+04	1.525E+05	6.425E+02	3.513E+05	1.644E+04	1.963E+02	3.016E+02	1.042E+04	4.132E+02	5.218E+02
	Rank	1	9	10	5	11	8	2	3	7	4	6
F8	Best	8.078E+02	3.105E+03	1.886E+03	9.818E+02	5.419E+03	9.507E+02	8.097E+02	8.162E+02	1.051E+03	8.416E+02	8.346E+02
	Mean	8.231E+02	4.681E+04	3.145E+04	3.009E+03	6.932E+05	2.497E+03	9.209E+02	9.387E+02	5.406E+03	1.488E+03	9.428E+02
	Std	1.093E+01	6.710E+04	5.305E+04	2.104E+03	1.922E+06	1.593E+03	1.085E+02	9.283E+01	1.158E+04	9.996E+02	9.180E+01
	Rank	1	10	9	7	11	6	2	3	8	5	4
F9	Best	9.548E+02	1.431E+03	1.745E+03	1.021E+03	4.053E+03	1.159E+03	1.133E+03	9.281E+02	1.046E+03	9.534E+02	9.168E+02
	Mean	1.048E+03	4.563E+04	1.633E+04	2.891E+03	1.535E+05	4.040E+03	2.736E+03	1.061E+03	3.246E+03	2.589E+03	9.879E+02
	Std	5.856E+01	5.147E+04	3.761E+04	2.966E+03	4.048E+05	3.303E+03	2.352E+03	1.466E+02	2.360E+03	1.752E+03	9.428E+01
	Rank	2	10	9	6	11	8	5	3	7	4	1
F10	Best	1.006E+03	1.354E+03	1.385E+03	1.024E+03	2.917E+03	1.051E+03	1.043E+03	1.009E+03	1.061E+03	1.088E+03	1.002E+03
	Mean	1.026E+03	9.588E+03	6.972E+03	1.544E+03	6.707E+04	1.783E+03	1.226E+03	1.092E+03	1.736E+03	2.028E+03	1.010E+03
	Std	1.005E+01	9.421E+03	5.736E+03	4.543E+02	1.075E+05	9.950E+02	2.408E+02	1.007E+02	7.097E+02	9.849E+02	6.277E+00
	Rank	2	10	9	5	11	7	4	3	6	8	1
F11	Best	6.520E+03	5.943E+04	6.438E+04	4.787E+03	6.518E+05	3.801E+04	6.365E+03	1.036E+04	6.084E+03	1.728E+04	1.821E+04
	Mean	1.871E+04	8.342E+05	8.034E+05	3.005E+04	1.108E+07	3.577E+05	6.456E+04	3.100E+04	2.763E+05	1.321E+05	7.773E+04
	Std	1.050E+04	1.121E+06	1.231E+06	3.584E+04	1.836E+07	4.147E+05	6.504E+04	2.170E+04	3.355E+05	1.645E+05	5.509E+04
	Rank	1	10	9	2	11	8	4	3	7	6	5
F12	Best	1.455E+04	1.594E+04	5.831E+04	3.619E+04	1.917E+05	4.842E+03	5.019E+03	4.888E+03	3.764E+04	1.241E+04	4.541E+03
	Mean	3.624E+04	8.017E+05	2.035E+06	4.280E+05	6.192E+06	5.873E+04	3.085E+04	2.192E+04	1.836E+05	1.296E+05	1.779E+04
	Std	1.760E+04	2.162E+06	2.990E+06	4.034E+05	7.920E+06	6.630E+04	2.617E+04	1.387E+04	1.151E+05	1.727E+05	1.211E+04
	Rank	4	9	10	8	11	5	3	2	7	6	1
F13	Best	4.087E+03	1.149E+05	1.305E+05	3.692E+04	6.974E+05	1.786E+04	1.932E+04	2.872E+04	6.110E+03	1.321E+04	1.881E+04
	Mean	9.945E+03	1.850E+06	2.197E+06	5.291E+05	2.745E+07	1.850E+05	8.500E+04	9.944E+04	1.028E+05	1.120E+05	6.634E+04
	Std	3.044E+03	2.558E+06	2.630E+06	4.527E+05	7.397E+07	2.031E+05	4.199E+04	7.629E+04	1.241E+05	8.024E+04	3.853E+04
	Rank	1	9	10	8	11	7	3	4	5	6	2
F14	Best	1.010E+04	7.971E+04	4.212E+04	3.076E+04	2.563E+05	7.832E+04	1.440E+04	4.123E+03	7.230E+03	9.238E+03	1.622E+04
	Mean	2.687E+04	3.149E+06	1.056E+06	5.000E+05	6.462E+07	1.331E+06	2.171E+05	1.951E+04	2.835E+05	1.042E+05	5.523E+04
	Std	1.033E+04	4.813E+06	1.807E+06	7.269E+05	9.993E+07	1.602E+06	3.031E+05	1.612E+04	2.447E+05	9.621E+04	4.385E+04
	Rank	2	10	8	7	11	9	5	1	6	4	3
F15	Best	3.076E+03	2.752E+04	9.549E+03	4.723E+03	1.816E+04	4.312E+03	4.775E+03	5.391E+03	3.784E+04	3.504E+03	2.199E+03
	Mean	5.584E+03	1.013E+06	2.888E+05	4.546E+04	1.467E+06	2.356E+04	1.570E+04	3.602E+04	2.779E+05	1.125E+04	7.057E+03
	Std	1.915E+03	2.539E+06	6.216E+05	8.403E+04	3.172E+06	2.109E+04	1.098E+04	2.600E+04	3.492E+05	8.404E+03	3.921E+03
	Rank	1	10	9	7	11	5	4	6	8	3	2
F16	Best	5.891E+03	6.522E+04	9.279E+04	5.140E+03	6.639E+04	8.032E+04	3.053E+03	6.967E+03	4.168E+04	2.422E+04	5.024E+03
	Mean	1.307E+04	5.498E+06	2.154E+06	8.586E+04	3.505E+06	7.112E+05	1.918E+04	4.132E+04	3.077E+05	1.670E+05	1.772E+04
	Std	4.504E+03	1.083E+07	4.496E+06	1.101E+05	6.700E+06	6.958E+05	1.359E+04	6.887E+04	3.452E+05	1.409E+05	7.497E+03
	Rank	1	11	9	5	10	8	3	4	7	6	2
F17	Best	1.190E+04	4.443E+04	2.355E+05	5.187E+03	6.394E+05	6.022E+04	7.847E+03	1.110E+04	1.630E+04	4.677E+03	9.541E+03
	Mean	2.830E+04	9.160E+05	3.683E+06	4.782E+04	2.948E+07	7.903E+05	4.844E+04	5.891E+04	1.840E+05	9.304E+04	3.445E+04
	Std	1.049E+04	1.072E+06	5.335E+06	3.721E+04	6.876E+07	1.396E+06	4.168E+04	5.289E+04	1.868E+05	1.296E+05	2.678E+04
	Rank	1	9	10	3	11	8	4	5	7	6	2
F18	Best	2.970E+03	2.938E+04	5.365E+04	7.725E+03	6.546E+05	1.405E+04	2.419E+03	5.831E+03	4.631E+04	1.385E+04	4.685E+03
	Mean	5.949E+03	7.179E+05	2.674E+06	1.033E+05	1.475E+07	2.272E+05	1.737E+04	2.290E+04	5.477E+05	7.364E+04	1.988E+04
	Std	1.609E+03	1.206E+06	4.994E+06	1.165E+05	2.099E+07	3.976E+05	1.494E+04	1.260E+04	5.749E+05	4.818E+04	1.167E+04
	Rank	1	9	10	6	11	7	2	4	8	5	3
F19	Best	4.264E+03	2.031E+05	6.629E+05	3.777E+03	3.890E+05	1.998E+04	5.658E+03	7.791E+03	2.546E+04	6.150E+03	7.192E+03
	Mean	8.949E+03	3.916E+06	1.535E+07	9.008E+04	2.347E+07	3.099E+05	3.657E+04	3.867E+04	2.837E+05	3.272E+04	3.565E+04
	Std	2.766E+03	5.406E+06	3.369E+07	1.138E+05	3.622E+07	3.619E+05	3.080E+04	2.279E+04	3.519E+05	3.287E+04	4.095E+04
	Rank	1	9	10	6	11	8	4	5	7	2	3
F20	Best	6.599E+03	6.307E+04	2.734E+05	1.592E+04	1.569E+05	1.753E+04	2.583E+03	7.331E+03	1.419E+04	4.894E+03	8.776E+03
	Mean	1.203E+04	2.062E+06	5.943E+06	1.296E+05	5.200E+06	2.561E+05	1.646E+04	4.308E+04	1.032E+05	7.890E+04	4.163E+04
	Std	3.315E+03	6.268E+06	1.162E+07	1.449E+05	6.390E+06	3.130E+05	1.504E+04	4.120E+04	1.279E+05	6.263E+04	3.253E+04
	Rank	1	9	11	7	10	8	2	4	6	5	3
F21	Best	3.971E+03	2.609E+04	3.230E+04	5.161E+03	1.699E+05	1.548E+04	4.320E+03	3.362E+03	8.785E+03	3.710E+03	3.113E+03
	Mean	6.807E+03	3.939E+05	5.199E+05	5.451E+04	2.418E+06	6.516E+04	9.334E+03	1.598E+04	7.254E+04	2.045E+04	6.623E+03
	Std	1.849E+03	5.145E+05	5.518E+05	1.167E+05	2.193E+06	4.663E+04	4.217E+03	1.095E+04	7.290E+04	1.416E+04	2.661E+03
	Rank	2	9	10	6	11	7	3	4	8	5	1
F22	Best	2.405E+03	3.854E+03	3.242E+03	2.892E+03	4.682E+03	2.386E+03	2.333E+03	2.216E+03	3.046E+03	2.460E+03	2.255E+03
	Mean	2.932E+03	2.285E+05	3.069E+04	7.715E+03	1.861E+05	5.388E+03	3.256E+03	2.349E+03	1.697E+04	6.955E+03	2.891E+03
	Std	2.907E+02	4.278E+05	3.658E+04	6.152E+03	2.331E+05	3.099E+03	8.003E+02	1.089E+02	1.826E+04	3.282E+03	6.148E+02
	Rank	3	11	9	7	10	5	4	1	8	6	2
F23	Best	2.455E+03	3.136E+03	3.065E+03	3.029E+03	7.566E+03	5.899E+03	2.352E+03	2.400E+03	2.455E+03	2.494E+03	2.428E+03
	Mean	2.848E+03	5.098E+04	2.900E+04	2.309E+04	3.158E+05	2.876E+04	3.473E+03	2.948E+03	7.472E+03	3.485E+03	3.526E+03
	Std	2.687E+02	8.821E+04	3.415E+04	1.631E+04	4.229E+05	3.040E+04	9.429E+02	6.251E+02	5.335E+03	8.761E+02	8.567E+02
	Rank	1	10	9	7	11	8	3	2	6	4	5
F24	Best	2.810E+03	8.708E+03	1.463E+04	4.192E+03	1.456E+04	7.442E+03	2.664E+03	2.827E+03	3.194E+03	6.238E+03	4.436E+03
	Mean	3.821E+03	1.334E+05	2.398E+05	2.722E+04	5.384E+05	7.450E+04	5.918E+03	5.320E+03	2.137E+04	3.458E+04	1.163E+04
	Std	7.477E+02	2.297E+05	2.902E+05	2.422E+04	7.867E+05	7.490E+04	3.842E+03	2.833E+03	2.620E+04	2.806E+04	9.980E+03
	Rank	1	9	10	6	11	8	3	2	5	7	4
F25	Best	2.803E+03	5.736E+03	1.031E+04	3.427E+03	3.175E+04	4.063E+03	3.053E+03	3.032E+03	7.465E+03	2.796E+03	4.009E+03
	Mean	3.244E+03	5.180E+05	3.102E+05	1.916E+04	6.806E+05	2.341E+04	8.257E+03	6.829E+03	2.896E+04	7.661E+03	1.174E+04
	Std	3.459E+02	9.976E+05	6.072E+05	1.846E+04	1.017E+06	1.868E+04	8.970E+03	2.985E+03	3.434E+04	5.235E+03	6.590E+03
	Rank	1	10	9	6	11	7	4	2	8	3	5
F26	Best	2.749E+03	1.262E+04	4.527E+03	1.978E+04	1.412E+05	9.407E+03	4.127E+03	3.060E+03	4.318E+03	4.339E+03	3.131E+03
	Mean	3.264E+03	9.526E+05	1.148E+05	1.016E+05	1.599E+06	8.485E+04	1.288E+04	5.136E+03	5.652E+04	2.265E+04	6.689E+03
	Std	2.389E+02	1.074E+06	1.337E+05	1.009E+05	2.322E+06	9.060E+04	1.020E+04	2.381E+03	8.611E+04	1.570E+04	3.194E+03
	Rank	1	10	9	8	11	7	4	2	6	5	3
F27	Best	3.086E+03	1.065E+04	1.176E+04	2.816E+03	1.029E+04	5.152E+03	2.994E+03	2.739E+03	3.557E+03	3.757E+03	2.795E+03
	Mean	3.721E+03	3.443E+05	1.699E+05	5.865E+03	3.527E+05	2.292E+04	6.092E+03	3.469E+03	1.565E+04	1.284E+04	3.159E+03
	Std	3.193E+02	8.226E+05	2.892E+05	2.933E+03	3.668E+05	2.868E+04	2.635E+03	6.413E+02	1.307E+04	9.790E+03	2.821E+02
	Rank	3	10	9	4	11	8	5	2	7	6	1
F28	Best	3.111E+03	7.596E+03	1.090E+04	2.999E+03	2.721E+04	3.893E+03	3.848E+03	2.922E+03	6.098E+03	4.407E+03	8.949E+03
	Mean	3.458E+03	1.662E+05	2.837E+05	6.565E+03	6.165E+05	2.588E+04	9.817E+03	3.863E+03	2.580E+04	1.326E+04	3.040E+04
	Std	3.995E+02	1.955E+05	5.825E+05	3.067E+03	7.867E+05	3.079E+04	9.429E+03	8.566E+02	3.506E+04	8.534E+03	2.381E+04
	Rank	1	9	10	3	11	7	4	2	6	5	8
F29	Best	3.221E+03	4.983E+03	1.065E+04	3.412E+03	4.455E+04	5.006E+03	3.740E+03	3.093E+03	4.946E+03	3.373E+03	3.764E+03
	Mean	3.723E+03	2.393E+05	2.878E+05	1.427E+04	1.196E+06	2.597E+04	7.334E+03	4.008E+03	3.422E+04	1.157E+04	1.049E+04
	Std	3.836E+02	5.140E+05	5.000E+05	1.279E+04	1.320E+06	2.193E+04	2.905E+03	7.237E+02	3.609E+04	7.064E+03	6.279E+03
	Rank	1	9	10	6	11	7	3	2	8	5	4
F30	Best	3.177E+03	8.325E+03	3.315E+04	4.407E+03	1.965E+04	3.253E+03	3.195E+03	3.176E+03	1.147E+04	4.253E+03	3.523E+03
	Mean	3.339E+03	2.673E+05	3.880E+05	1.203E+04	1.225E+06	1.492E+04	4.635E+03	4.041E+03	5.592E+04	1.014E+04	7.369E+03
	Std	9.607E+01	7.481E+05	6.016E+05	6.379E+03	1.364E+06	1.631E+04	1.997E+03	7.910E+02	7.516E+04	5.879E+03	3.487E+03
	Rank	1	9	10	6	11	7	3	2	8	5	4

**Table A8:** Statistical results of MSSBOA and the comparison algorithms on CEC2017 (D = 100).

Func	Index	MSSBOA	SBOA	GWO	COA	SCA	SMA	RIME	QIO	MRFO	GKSO	LSHADE
F1	Best	1.005E+02	6.971E+02	3.123E+02	1.277E+02	4.113E+03	3.492E+02	1.338E+02	1.011E+02	2.067E+02	1.164E+02	1.005E+02
	Mean	1.013E+02	2.142E+04	5.820E+03	3.757E+02	8.005E+04	3.990E+03	3.976E+02	1.034E+02	1.108E+03	2.473E+02	1.013E+02
	Std	5.750E-01	3.306E+04	5.616E+03	3.289E+02	1.332E+05	6.448E+03	2.710E+02	1.209E+00	9.869E+02	1.128E+02	3.584E-01
	Rank	1	10	9	5	11	8	6	3	7	4	2
F3	Best	3.000E+02	3.978E+02	3.487E+02	3.051E+02	6.091E+02	3.275E+02	3.133E+02	3.005E+02	3.024E+02	3.055E+02	3.001E+02
	Mean	3.001E+02	1.763E+03	1.186E+03	3.522E+02	5.822E+03	8.423E+02	3.878E+02	3.009E+02	3.316E+02	3.793E+02	3.004E+02
	Std	5.320E-02	1.705E+03	8.822E+02	5.099E+01	1.087E+04	7.372E+02	7.573E+01	3.384E-01	3.461E+01	9.756E+01	1.326E-01
	Rank	1	10	9	5	11	8	7	3	4	6	2
F4	Best	2.198E+03	4.435E+04	3.810E+04	3.923E+03	1.079E+05	1.199E+04	3.071E+03	2.050E+03	2.886E+04	8.507E+03	2.166E+03
	Mean	6.604E+03	2.102E+06	1.180E+06	3.870E+04	9.633E+06	1.016E+05	2.147E+04	2.792E+04	4.894E+05	8.113E+04	9.601E+03
	Std	2.700E+03	3.804E+06	1.419E+06	3.834E+04	1.586E+07	1.311E+05	1.682E+04	2.243E+04	4.839E+05	8.086E+04	6.726E+03
	Rank	1	10	9	5	11	7	3	4	8	6	2
F5	Best	8.966E+02	6.703E+04	1.650E+04	1.155E+04	2.832E+05	2.465E+03	9.754E+02	1.297E+03	4.511E+03	2.358E+03	8.760E+02
	Mean	1.631E+03	8.592E+05	3.832E+05	1.188E+05	5.381E+06	5.811E+04	3.667E+03	6.999E+03	8.855E+04	2.840E+04	2.241E+03
	Std	5.615E+02	1.161E+06	4.868E+05	1.172E+05	6.790E+06	5.613E+04	3.052E+03	8.079E+03	1.031E+05	2.787E+04	8.629E+02
	Rank	1	10	9	8	11	6	3	4	7	5	2
F6	Best	1.193E+03	9.812E+03	4.153E+03	2.351E+03	3.747E+05	1.731E+03	1.521E+03	1.199E+03	3.517E+03	2.966E+03	1.324E+03
	Mean	2.298E+03	6.934E+05	1.262E+05	1.390E+04	2.292E+06	2.072E+04	1.600E+04	6.390E+03	9.043E+04	2.334E+04	5.432E+03
	Std	5.923E+02	1.141E+06	9.734E+04	1.725E+04	2.816E+06	2.331E+04	1.239E+04	4.637E+03	9.768E+04	2.933E+04	3.931E+03
	Rank	1	10	9	4	11	6	5	3	8	7	2
F7	Best	1.344E+03	1.306E+04	3.550E+04	7.674E+03	6.199E+04	1.074E+04	1.325E+03	1.184E+03	2.871E+03	6.097E+03	1.026E+03
	Mean	2.147E+03	6.602E+05	1.212E+06	5.466E+04	1.360E+06	8.650E+04	5.548E+03	4.418E+03	2.908E+04	2.751E+04	2.882E+03
	Std	6.234E+02	1.456E+06	2.589E+06	3.524E+04	1.968E+06	8.064E+04	6.533E+03	4.106E+03	3.411E+04	2.279E+04	1.667E+03
	Rank	1	9	10	7	11	8	4	3	6	5	2
F8	Best	1.962E+03	3.829E+04	3.010E+04	7.959E+03	7.211E+04	2.250E+03	1.014E+03	2.284E+03	6.279E+03	4.531E+03	4.459E+03
	Mean	3.859E+03	7.025E+05	1.236E+06	4.315E+04	2.185E+06	4.232E+04	5.480E+03	8.325E+03	5.336E+04	2.394E+04	2.793E+04
	Std	1.192E+03	7.310E+05	2.157E+06	3.196E+04	3.276E+06	5.947E+04	1.023E+04	4.338E+03	5.470E+04	1.951E+04	2.621E+04
	Rank	1	9	10	7	11	6	2	3	8	4	5
F9	Best	2.984E+03	2.397E+04	1.043E+04	2.357E+03	4.418E+04	2.133E+03	4.682E+03	4.203E+03	6.876E+03	2.166E+03	1.486E+03
	Mean	6.111E+03	4.678E+05	2.713E+05	2.927E+04	2.541E+06	4.084E+04	2.987E+04	2.383E+04	7.483E+04	1.160E+04	2.963E+03
	Std	2.325E+03	9.812E+05	2.654E+05	2.840E+04	2.671E+06	4.522E+04	2.685E+04	2.830E+04	9.746E+04	1.122E+04	1.060E+03
	Rank	2	10	9	5	11	7	6	4	8	3	1
F10	Best	1.808E+03	1.117E+04	4.079E+03	4.168E+03	3.923E+03	3.377E+03	1.643E+03	1.151E+03	2.374E+03	2.856E+03	1.175E+03
	Mean	2.887E+03	5.719E+05	2.325E+05	8.369E+04	1.441E+05	3.022E+04	6.232E+03	1.962E+03	5.683E+04	2.145E+04	1.937E+03
	Std	8.127E+02	1.478E+06	2.513E+05	9.110E+04	2.383E+05	3.912E+04	6.938E+03	8.996E+02	6.239E+04	1.639E+04	6.738E+02
	Rank	3	11	10	8	9	6	4	2	7	5	1
F11	Best	5.313E+05	1.566E+06	7.635E+06	2.032E+06	4.570E+07	2.080E+06	3.626E+05	1.279E+06	8.487E+05	3.261E+05	2.077E+06
	Mean	9.377E+05	1.755E+08	1.346E+08	3.505E+07	3.116E+09	1.858E+07	3.146E+06	5.779E+06	1.918E+07	5.887E+06	8.218E+06
	Std	3.570E+05	4.146E+08	1.462E+08	3.911E+07	3.321E+09	1.932E+07	2.961E+06	4.895E+06	2.678E+07	8.055E+06	7.813E+06
	Rank	1	10	9	8	11	6	2	3	7	4	5
F12	Best	2.048E+06	8.171E+07	7.704E+07	3.628E+06	1.877E+08	7.218E+06	6.621E+05	3.495E+06	9.480E+06	2.036E+06	4.141E+06
	Mean	6.398E+06	1.341E+09	1.197E+09	4.298E+07	1.498E+10	1.127E+08	6.178E+06	2.424E+07	1.479E+08	4.097E+07	1.611E+07
	Std	3.324E+06	1.859E+09	1.286E+09	3.919E+07	4.501E+10	1.065E+08	7.255E+06	2.400E+07	2.267E+08	4.954E+07	1.298E+07
	Rank	2	10	9	6	11	7	1	4	8	5	3
F13	Best	1.390E+06	6.260E+06	6.517E+06	8.156E+06	4.224E+07	4.900E+06	1.040E+06	1.846E+06	6.928E+06	1.257E+06	9.317E+05
	Mean	3.226E+06	2.379E+08	5.020E+08	1.344E+08	1.165E+09	4.238E+07	1.124E+07	8.535E+06	8.773E+07	1.747E+07	1.111E+07
	Std	1.392E+06	2.495E+08	6.020E+08	2.213E+08	1.345E+09	3.627E+07	1.722E+07	7.515E+06	1.833E+08	1.375E+07	9.246E+06
	Rank	1	9	10	8	11	6	4	2	7	5	3
F14	Best	4.981E+06	5.057E+07	3.769E+07	6.642E+06	2.509E+07	2.375E+06	3.582E+06	4.654E+06	5.818E+06	1.610E+06	9.669E+05
	Mean	1.284E+07	7.153E+08	8.654E+08	7.964E+07	4.798E+08	8.691E+07	2.311E+07	2.905E+07	7.252E+07	2.193E+07	4.598E+06
	Std	6.033E+06	1.085E+09	1.656E+09	6.131E+07	6.310E+08	8.855E+07	2.509E+07	2.997E+07	7.996E+07	1.714E+07	3.879E+06
	Rank	2	10	11	7	9	8	4	5	6	3	1
F15	Best	1.510E+06	1.462E+07	2.342E+07	7.076E+06	4.741E+07	5.780E+05	6.581E+05	7.119E+05	2.165E+06	2.383E+05	1.890E+06
	Mean	3.564E+06	4.905E+08	1.181E+09	5.558E+07	1.411E+09	2.751E+07	3.313E+06	7.124E+06	4.761E+07	3.418E+06	1.687E+07
	Std	1.821E+06	8.265E+08	1.995E+09	7.190E+07	2.377E+09	6.062E+07	4.173E+06	1.043E+07	6.381E+07	4.045E+06	1.010E+07
	Rank	3	9	10	8	11	6	1	4	7	2	5
F16	Best	9.173E+06	1.434E+07	1.324E+07	1.383E+07	1.635E+08	1.396E+07	5.244E+06	7.324E+05	1.591E+07	1.454E+06	2.352E+06
	Mean	2.671E+07	1.380E+09	1.889E+08	1.465E+08	2.626E+09	3.737E+08	8.213E+07	4.456E+06	1.218E+08	1.073E+07	2.076E+07
	Std	1.077E+07	2.062E+09	2.484E+08	1.381E+08	3.246E+09	3.813E+08	9.663E+07	4.436E+06	1.286E+08	1.501E+07	1.279E+07
	Rank	4	10	8	7	11	9	5	1	6	2	3
F17	Best	7.563E+06	2.638E+07	1.413E+08	5.278E+06	4.320E+07	2.406E+07	1.892E+06	3.542E+06	1.680E+07	7.761E+06	1.277E+07
	Mean	1.626E+07	1.014E+09	2.152E+09	1.025E+08	2.917E+09	2.550E+08	2.368E+07	2.397E+07	2.642E+08	8.982E+07	6.148E+07
	Std	6.335E+06	1.977E+09	3.771E+09	9.391E+07	5.186E+09	2.040E+08	3.002E+07	1.750E+07	3.514E+08	9.020E+07	4.390E+07
	Rank	1	9	10	6	11	7	2	3	8	5	4
F18	Best	6.876E+05	9.526E+06	3.728E+07	2.945E+06	1.566E+08	4.376E+06	6.062E+05	3.266E+06	7.921E+06	3.790E+05	1.204E+06
	Mean	2.532E+06	6.503E+08	1.521E+09	5.289E+07	8.969E+09	1.102E+08	4.722E+06	2.339E+07	1.247E+08	8.324E+06	5.094E+06
	Std	9.987E+05	7.182E+08	1.199E+09	3.507E+07	1.050E+10	1.612E+08	4.220E+06	2.096E+07	1.251E+08	8.413E+06	3.504E+06
	Rank	1	9	10	6	11	7	2	5	8	4	3
F19	Best	1.429E+06	1.312E+08	1.162E+09	1.821E+06	1.728E+08	6.234E+06	4.669E+05	3.539E+06	7.119E+06	6.078E+06	6.647E+05
	Mean	4.324E+06	4.553E+09	1.170E+10	6.466E+07	1.684E+10	8.753E+07	4.530E+06	1.517E+07	7.138E+07	8.116E+07	8.669E+06
	Std	1.741E+06	9.206E+09	1.639E+10	7.479E+07	3.047E+10	8.606E+07	1.226E+07	1.334E+07	8.347E+07	8.552E+07	8.630E+06
	Rank	1	9	10	5	11	8	2	4	6	7	3
F20	Best	1.164E+06	3.153E+06	9.015E+06	3.195E+06	8.563E+07	4.464E+06	2.019E+06	6.341E+05	8.627E+06	7.544E+05	1.407E+06
	Mean	4.106E+06	1.407E+08	6.514E+08	7.635E+07	1.673E+09	1.512E+08	1.424E+07	1.064E+07	7.479E+07	1.804E+07	6.615E+06
	Std	1.375E+06	4.292E+08	9.589E+08	1.823E+08	2.098E+09	2.185E+08	1.365E+07	8.896E+06	7.945E+07	2.441E+07	3.270E+06
	Rank	1	8	10	7	11	9	4	3	6	5	2
F21	Best	1.365E+04	1.775E+05	2.646E+05	4.296E+04	4.565E+06	1.133E+06	4.629E+04	3.699E+04	5.923E+05	3.646E+04	6.268E+04
	Mean	2.979E+04	6.489E+06	3.874E+06	1.114E+06	4.393E+07	2.563E+07	1.859E+05	2.664E+05	8.483E+06	6.244E+05	1.936E+05
	Std	1.382E+04	1.204E+07	3.856E+06	8.304E+05	4.952E+07	2.643E+07	1.525E+05	1.811E+05	9.165E+06	7.589E+05	1.185E+05
	Rank	1	8	7	6	11	10	2	4	9	5	3
F22	Best	5.613E+04	5.211E+05	2.106E+05	2.083E+05	6.530E+06	1.316E+05	1.213E+05	7.182E+04	8.003E+05	3.628E+04	9.405E+04
	Mean	1.194E+05	2.739E+07	2.368E+07	3.787E+06	1.147E+08	2.473E+06	1.305E+06	3.310E+05	9.897E+06	5.174E+05	4.050E+05
	Std	4.447E+04	4.352E+07	2.166E+07	4.728E+06	1.669E+08	3.895E+06	1.175E+06	2.159E+05	1.001E+07	4.766E+05	2.409E+05
	Rank	1	10	9	7	11	6	5	2	8	4	3
F23	Best	3.607E+04	3.720E+05	1.089E+06	8.309E+04	2.995E+06	8.309E+04	5.116E+04	5.727E+04	5.324E+05	1.355E+04	3.987E+05
	Mean	7.444E+04	1.937E+07	6.189E+07	9.444E+05	1.058E+08	1.495E+06	2.132E+05	2.766E+05	3.962E+06	2.104E+05	1.564E+06
	Std	2.643E+04	3.259E+07	7.701E+07	1.267E+06	2.171E+08	1.442E+06	1.445E+05	2.578E+05	5.062E+06	1.882E+05	1.219E+06
	Rank	1	9	10	5	11	6	3	4	8	2	7
F24	Best	1.095E+05	5.181E+05	4.455E+06	2.028E+05	1.733E+06	4.926E+05	2.475E+05	6.177E+04	2.152E+05	2.832E+05	1.470E+05
	Mean	2.394E+05	3.242E+07	3.995E+07	1.449E+06	1.042E+08	6.178E+06	1.208E+06	5.099E+05	1.759E+06	3.279E+06	6.088E+05
	Std	8.414E+04	4.600E+07	5.651E+07	1.196E+06	2.248E+08	7.451E+06	1.063E+06	3.076E+05	1.687E+06	3.413E+06	3.759E+05
	Rank	1	9	10	5	11	8	4	2	6	7	3
F25	Best	3.505E+04	2.605E+05	6.344E+05	7.598E+04	2.186E+06	1.352E+05	3.414E+04	2.683E+04	1.903E+04	1.006E+05	6.865E+04
	Mean	7.608E+04	2.526E+07	2.388E+07	2.599E+06	9.772E+07	2.432E+06	1.826E+05	1.037E+05	7.210E+05	1.833E+06	5.375E+05
	Std	2.603E+04	5.110E+07	4.004E+07	2.949E+06	1.885E+08	3.292E+06	2.211E+05	7.819E+04	7.369E+05	3.977E+06	4.324E+05
	Rank	1	10	9	8	11	7	3	2	5	6	4
F26	Best	5.676E+04	1.547E+05	1.435E+05	7.657E+04	1.098E+06	7.152E+04	1.389E+04	4.568E+04	6.128E+05	3.880E+05	2.303E+04
	Mean	1.659E+05	9.606E+06	1.529E+07	7.354E+05	6.505E+07	5.077E+05	5.959E+04	1.652E+05	4.283E+06	5.479E+06	3.500E+05
	Std	9.318E+04	1.681E+07	1.779E+07	7.080E+05	1.675E+08	4.441E+05	7.157E+04	1.315E+05	5.478E+06	6.235E+06	2.214E+05
	Rank	3	9	10	6	11	5	1	2	7	8	4
F27	Best	7.859E+04	7.065E+04	5.360E+05	4.118E+05	5.279E+06	1.118E+05	3.438E+04	6.071E+03	6.635E+04	1.286E+04	2.359E+04
	Mean	2.065E+05	4.938E+06	1.897E+07	4.092E+06	9.514E+07	1.358E+06	1.397E+05	4.567E+04	4.880E+05	1.938E+05	8.572E+04
	Std	7.089E+04	5.785E+06	3.379E+07	4.429E+06	1.353E+08	1.998E+06	1.391E+05	4.793E+04	5.657E+05	2.038E+05	6.344E+04
	Rank	5	9	10	8	11	7	3	1	6	4	2
F28	Best	6.835E+05	1.088E+06	7.844E+05	1.691E+05	5.678E+06	5.314E+05	2.717E+04	5.572E+04	2.244E+04	1.738E+05	1.943E+05
	Mean	1.235E+06	3.083E+07	3.547E+07	4.588E+06	4.459E+08	6.542E+06	9.477E+05	3.269E+05	9.311E+05	4.798E+06	1.369E+06
	Std	4.764E+05	2.760E+07	7.563E+07	5.281E+06	6.462E+08	5.414E+06	8.248E+05	2.101E+05	1.084E+06	6.826E+06	1.025E+06
	Rank	4	9	10	6	11	8	3	1	2	7	5
F29	Best	6.351E+04	9.415E+04	2.923E+05	7.486E+04	2.419E+06	1.121E+05	2.325E+04	9.153E+03	3.058E+05	1.517E+05	4.215E+04
	Mean	1.775E+05	9.199E+06	8.961E+06	7.868E+05	9.431E+07	1.993E+06	2.191E+05	4.234E+04	3.171E+06	9.176E+05	1.669E+05
	Std	7.041E+04	1.555E+07	1.044E+07	1.183E+06	1.085E+08	2.158E+06	1.359E+05	3.529E+04	4.316E+06	1.036E+06	9.498E+04
	Rank	3	10	9	5	11	7	4	1	8	6	2
F30	Best	1.636E+05	2.636E+05	1.398E+06	8.778E+04	8.142E+05	9.665E+04	2.208E+04	2.403E+04	1.845E+05	1.577E+05	8.651E+04
	Mean	3.761E+05	1.235E+07	2.700E+07	8.088E+05	1.171E+07	2.944E+06	1.179E+05	1.361E+05	8.140E+05	1.781E+06	4.023E+05
	Std	1.389E+05	1.620E+07	4.203E+07	5.808E+05	1.335E+07	4.590E+06	9.999E+04	8.918E+04	4.808E+05	2.130E+06	3.183E+05
	Rank	3	10	11	5	9	8	1	2	6	7	4

## References

[1] Jin B., Vai M.I. (2015). An Adaptive Ultrasonic Backscattered Signal Processing Technique for Accurate Object Localization Based on the Instantaneous Energy Density Level. J. Med. Imaging Health Inform..

[2] Slowik A., Kwasnicka H. (2018). Nature Inspired Methods and Their Industry Applications-Swarm Intelligence Algorithms. IEEE Trans. Ind. Inform..

[3] Tang A., Zhou H., Han T., Xie L. (2021). A Modified Manta Ray Foraging Optimization for Global Optimization Problems. IEEE Access.

[4] Jin B., Gonçalves N., Cruz L., Medvedev I., Yu Y., Wang J. (2024). Simulated Multimodal Deep Facial Diagnosis. Expert Syst. Appl..

[5] Tang A.D., Tang S.Q., Han T., Zhou H., Xie L. (2021). A Modified Slime Mould Algorithm for Global Optimization. Comput. Intell. Neurosci..

[6] Dai M., Tang D., Giret A., Salido M.A. (2019). Multi-Objective Optimization for Energy-Efficient Flexible Job Shop Scheduling Problem with Transportation Constraints. Robot. Comput. Integr. Manuf..

[7] Tang A., Zhou H., Han T., Xie L. (2021). A Chaos Sparrow Search Algorithm with Logarithmic Spiral and Adaptive Step for Engineering Problems. Comput. Model. Eng. Sci..

[8] Shen X., Du S.C., Sun Y.N., Sun P.Z.H., Law R., Wu E.Q. (2023). Advance Scheduling for Chronic Care Under Online or Offline Revisit Uncertainty. IEEE Trans. Autom. Sci. Eng..

[9] Cao B., Fan S., Zhao J., Tian S., Zheng Z., Yan Y., Yang P. (2021). Large-Scale Many-Objective Deployment Optimization of Edge Servers. IEEE Trans. Intell. Transp. Syst..

[10] Zeng N., Wang Z., Liu W., Zhang H., Hone K., Liu X. (2022). A Dynamic Neighborhood-Based Switching Particle Swarm Optimization Algorithm. IEEE Trans. Cybern..

[11] Wang C., Koh J.M., Yu T., Xie N.G., Cheong K.H. (2020). Material and Shape Optimization of Bi-Directional Functionally Graded Plates by GIGA and an Improved Multi-Objective Particle Swarm Optimization Algorithm. Comput. Methods Appl. Mech. Eng..

[12] Zhao W., Du C., Jiang S. (2018). An Adaptive Multiscale Approach for Identifying Multiple Flaws Based on XFEM and a Discrete Artificial Fish Swarm Algorithm. Comput. Methods Appl. Mech. Eng..

[13] Tang A.D., Han T., Zhou H., Xie L. (2021). An Improved Equilibrium Optimizer with Application in Unmanned Aerial Vehicle Path Planning. Sensors.

[14] Hu G., Huang F.Y., Shu B., Wei G. (2025). MAHACO: Multi-Algorithm Hybrid Ant Colony Optimizer for 3D Path Planning of a Group of UAVs. Inf. Sci..

[15] Sowmya R., Premkumar M., Jangir P. (2024). Newton-Raphson-Based Optimizer: A New Population-Based Metaheuristic Algorithm for Continuous Optimization Problems. Eng. Appl. Artif. Intell..

[16] Houssein E.H., Saad M.R., Çelik E., Hu G., Ali A.A., Shaban H. (2024). An Enhanced Sea-Horse Optimizer for Solving Global Problems and Cluster Head Selection in Wireless Sensor Networks. Clust. Comput. J. Netw. Softw. Tools Appl..

[17] Abualigah L., Habash M., Hanandeh E.S., Hussein A.M.A., Shinwan M.A., Zitar R.A., Jia H. (2023). Improved Reptile Search Algorithm by Salp Swarm Algorithm for Medical Image Segmentation. J. Bionic Eng..

[18] Hu G., Zheng Y.X., Houssein E.H., Wei G. (2024). GSRPSO: A Multi-Strategy Integrated Particle Swarm Algorithm for Multi-Threshold Segmentation of Real Cervical Cancer Images. Swarm Evol. Comput..

[19] Seyyedabbasi A., Hu G., Shehadeh H.A., Wang X.P., Canatalay P.J. (2025). V-Shaped and S-Shaped Binary Artificial Protozoa Optimizer (APO) Algorithm for Wrapper Feature Selection on Biological Data. Clust. Comput. J. Netw. Softw. Tools Appl..

[20] Jia H., Sun K., Li Y., Cao N. (2022). Improved Marine Predators Algorithm for Feature Selection and SVM Optimization. KSII Trans. Internet Inf. Syst..

[21] Holland J.H. (1992). Genetic Algorithms. Sci. Am..

[22] Beyer H.-G., Schwefel H.-P. (2002). Evolution Strategies—A Comprehensive Introduction. Nat. Comput..

[23] Opara K.R., Arabas J. (2019). Differential Evolution: A Survey of Theoretical Analyses. Swarm Evol. Comput..

[24] Hansen N., Ostermeier A. (2001). Completely Derandomized Self-Adaptation in Evolution Strategies. Evol. Comput..

[25] Lian J., Hui G. (2024). Human Evolutionary Optimization Algorithm. Expert Syst. Appl..

[26] Dong Y.C., Zhang S.H., Zhang H.L., Zhou X.J., Jiang J.D. (2025). Chaotic Evolution Optimization: A Novel Metaheuristic Algorithm Inspired by Chaotic Dynamics. Chaos Solitons Fractals.

[27] Mirjalili S., Mirjalili S.M., Hatamlou A. (2016). Multi-Verse Optimizer: A Nature-Inspired Algorithm for Global Optimization. Neural Comput. Appl..

[28] Hashim F.A., Mostafa R.R., Hussien A.G., Mirjalili S., Sallam K.M. (2023). Fick’s Law Algorithm: A Physical Law-Based Algorithm for Numerical Optimization. Knowl.-Based Syst..

[29] Deng L., Liu S. (2023). Snow Ablation Optimizer: A Novel Metaheuristic Technique for Numerical Optimization and Engineering Design. Expert Syst. Appl..

[30] Yuan C., Zhao D., Heidari A.A., Liu L., Chen Y., Chen H. (2024). Polar Lights Optimizer: Algorithm and Applications in Image Segmentation and Feature Selection. Neurocomputing.

[31] Goodarzimehr V., Shojaee S., Hamzehei-Javaran S., Talatahari S. (2022). Special Relativity Search: A Novel Metaheuristic Method Based on Special Relativity Physics. Knowl.-Based Syst..

[32] He J.H., Zhao S.J., Ding J.Y., Wang Y.M. (2025). Mirage Search Optimization: Application to Path Planning and Engineering Design Problems. Adv. Eng. Softw..

[33] Abdel-Basset M., Mohamed R., Azeem S.A.A., Jameel M., Abouhawwash M. (2023). Kepler Optimization Algorithm: A New Metaheuristic Algorithm Inspired by Kepler’s Laws of Planetary Motion. Knowl.-Based Syst..

[34] Mirjalili S. (2016). SCA: A Sine Cosine Algorithm for Solving Optimization Problems. Knowl.-Based Syst..

[35] Abualigah L., Diabat A., Mirjalili S., Abd Elaziz M., Gandomi A.H. (2021). The Arithmetic Optimization Algorithm. Comput. Methods Appl. Mech. Eng..

[36] Beltran L.A., Navarro M.A., Oliva D., Campos-Peña D., Ramos-Frutos J., Zapotecas-Martínez S. (2024). Quasi-Random Fractal Search (QRFS): A Dynamic Metaheuristic with Sigmoid Population Decrement for Global Optimization. Expert Syst. Appl..

[37] Cheng J., De Waele W. (2024). Weighted Average Algorithm: A Novel Meta-Heuristic Optimization Algorithm Based on the Weighted Average Position Concept. Knowl.-Based Syst..

[38] Zhao S.J., Zhang T.R., Cai L., Yang R.H. (2024). Triangulation Topology Aggregation Optimizer: A Novel Mathematics-Based Meta-Heuristic Algorithm for Continuous Optimization and Engineering Applications. Expert Syst. Appl..

[39] Luan T.M., Khatir S., Tran M.T., De Baets B., Cuong-Le T. (2024). Exponential-Trigonometric Optimization Algorithm for Solving Complicated Engineering Problems. Comput. Methods Appl. Mech. Eng..

[40] Huang W., Xu J. (2023). Particle Swarm Optimization. Springer Tracts in Civil Engineering.

[41] Dorigo M., Di Caro G. Ant Colony Optimization: A New Meta-Heuristic. Proceedings of the 1999 Congress on Evolutionary Computation, CEC 1999.

[42] Mirjalili S., Mirjalili S.M., Lewis A. (2014). Grey Wolf Optimizer. Adv. Eng. Softw..

[43] Xiao Y.N., Cui H., Abu Khurma R., Castillo P.A. (2025). Artificial Lemming Algorithm: A Novel Bionic Meta-Heuristic Technique for Solving Real-World Engineering Optimization Problems. Artif. Intell. Rev..

[44] Jia H., Rao H., Wen C., Mirjalili S. (2023). Crayfish Optimization Algorithm. Artif. Intell. Rev..

[45] Agushaka J.O., Ezugwu A.E., Abualigah L. (2022). Dwarf Mongoose Optimization Algorithm. Comput. Methods Appl. Mech. Eng..

[46] Hu G., Cheng M., Houssein E.H., Hussien A.G., Abualigah L. (2024). SDO: A Novel Sled Dog-Inspired Optimizer for Solving Engineering Problems. Adv. Eng. Inform..

[47] Li S., Chen H., Wang M., Heidari A.A., Mirjalili S. (2020). Slime Mould Algorithm: A New Method for Stochastic Optimization. Future Gener. Comput. Syst..

[48] Wang W.C., Tian W.C., Xu D.M., Zang H.F. (2024). Arctic Puffin Optimization: A Bio-Inspired Metaheuristic Algorithm for Solving Engineering Design Optimization. Adv. Eng. Softw..

[49] Hu G., Guo Y., Wei G., Abualigah L. (2023). Genghis Khan Shark Optimizer: A Novel Nature-Inspired Algorithm for Engineering Optimization. Adv. Eng. Inform..

[50] Ezugwu A.E., Agushaka J.O., Abualigah L., Mirjalili S., Gandomi A.H. (2022). Prairie Dog Optimization Algorithm. Neural Comput. Appl..

[51] Fu Y.F., Liu D., Chen J.D., He L. (2024). Secretary Bird Optimization Algorithm: A New Metaheuristic for Solving Global Optimization Problems. Artif. Intell. Rev..

[52] Wolpert D.H., Macready W.G. (1997). No Free Lunch Theorems for Optimization. IEEE Trans. Evol. Comput..

[53] Qin S., Liu J., Bai X., Hu G. (2024). A Multi-Strategy Improvement Secretary Bird Optimization Algorithm for Engineering Optimization Problems. Biomimetics.

[54] Wang X., Wei P., Li Y. (2025). Enhanced Secretary Bird Optimization Algorithm with Multi-Strategy Fusion and Cauchy–Gaussian Crossover. Sci. Rep..

[55] Mai X., Zhong Y., Li L. (2025). The Crossover Strategy Integrated Secretary Bird Optimization Algorithm and Its Application in Engineering Design Problems. Electron. Res. Arch..

[56] Seyyedabbasi A. (2026). Multi-Strategy Variable Secretary Bird Optimization Algorithm (MSVSBOA) for Global Optimization and UAV 3D Path Planning. Symmetry.

[57] Safari A., Varaee H. (2023). Opposition-Based Ideal Gas Molecular Movement Algorithm with Cauchy Mutation, Velocity Clamping, and Mirror Operator. Expert Syst..

[58] Yang X.-S., Deb S. Cuckoo Search via Levy Flights. Proceedings of the World Congress on Nature & Biologically Inspired Computing (NaBIC).

[59] Hua L.K., Wang Y. (1981). Applications of Number Theory to Numerical Analysis.

[60] Zhang X., Wang S., Peng H., Li Y., Qi J., Kan Z., Meng H. (2024). A Modified Sand Cat Swarm Optimization Algorithm Based on Multi-Strategy Fusion and Its Application in Engineering Problems. Mathematics.

[61] Tizhoosh H.R. Opposition-Based Learning: A New Scheme for Machine Intelligence. Proceedings of the International Conference on Computational Intelligence for Modelling, Control and Automation (CIMCA).

[62] Rahnamayan S., Tizhoosh H.R., Salama M.M.A. Quasi-Oppositional Differential Evolution. Proceedings of the IEEE Congress on Evolutionary Computation (CEC).

[63] Long W., Jiao J., Wu T., Xu M., Tang M. (2024). An Improved Sand Cat Swarm Optimization with Lens Opposition-Based Learning. Sci. Rep..

[64] Yao X., Liu Y., Lin G. (1999). Evolutionary Programming Made Faster. IEEE Trans. Evol. Comput..

[65] Awad N.H., Ali M.Z., Liang J.J., Qu B.Y., Suganthan P.N. (2016). Problem Definitions and Evaluation Criteria for the CEC 2017 Special Session and Competition on Single Objective Bound Constrained Real-Parameter Numerical Optimization.

[66] Zhao W., Zhang Z., Wang L. (2020). Manta Ray Foraging Optimization: An Effective Bio-Inspired Optimizer for Engineering Applications. Eng. Appl. Artif. Intell..

[67] Su H., Zhao D., Heidari A.A., Liu L., Zhang X., Mafarja M., Chen H. (2023). RIME: A Physics-Based Optimization. Neurocomputing.

[68] Zhao W., Wang L., Zhang Z., Mirjalili S., Khodadadi N., Ge Q. (2023). Quadratic Interpolation Optimization (QIO): A New Optimization Algorithm Based on Generalized Quadratic Interpolation and Its Applications to Real-World Engineering Problems. Comput. Methods Appl. Mech. Eng..

[69] Tanabe R., Fukunaga A.S. Improving the Search Performance of SHADE Using Linear Population Size Reduction. Proceedings of the 2014 IEEE Congress on Evolutionary Computation, CEC 2014, Beijing, China.

[70] Cohen-Or D., Sorkine O., Gal R., Leyvand T., Xu Y.-Q. (2006). Color Harmonization. ACM Trans. Graph..

[71] Hu G., Pan Z., Zhang M., Chen D., Yang W., Chen J. (2014). An Interactive Method for Generating Harmonious Color Schemes. Color Res. Appl..

[72] Tokumaru M., Muranaka N., Imanishi S. Color Design Support System Considering Color Harmony. Proceedings of the 2002 IEEE International Conference on Fuzzy Systems (FUZZ-IEEE).

[73] Ngo D.C.L., Teo L.S., Byrne J.G. (2003). Modelling Interface Aesthetics. Inf. Sci..

[74] O’Donovan P., Agarwala A., Hertzmann A. (2014). Learning Layouts for Single-Page Graphic Designs. IEEE Trans. Vis. Comput. Graph..

[75] Lai C.-Y., Chen P.-H., Shih S.-W., Liu Y., Hong J.-S. (2010). Computational Models and Experimental Investigations of Effects of Balance and Symmetry on the Aesthetics of Text-Overlaid Images. Int. J. Hum.-Comput. Stud..

[76] Deng L., Zhou F., Zhang Z. (2022). Interactive Genetic Color Matching Design of Cultural and Creative Products Considering Color Image and Visual Aesthetics. Heliyon.

[77] Jiang Z., Xia J., Xu Y., Zhang Y. (2025). Cultural Heritage Color Regeneration: Interactive Genetic Algorithm Optimization Based on Color Network and Harmony Models. Appl. Sci..

